# Genome-wide transcriptional profiling of wheat infected with *Fusarium graminearum*

**DOI:** 10.1016/j.gdata.2015.06.020

**Published:** 2015-06-23

**Authors:** Ayumi Kosaka, Tomohiro Ban, Alagu Manickavelu

**Affiliations:** Plant Genetic Resource Division, Kihara Institute for Biological Research, Yokohama City University, Maioka 641-12, Totsuka, Yokohama 244-0813, Japan

**Keywords:** Common wheat, *Fusarium graminearum*, Microarray

## Abstract

Fusarium head blight (FHB) is a destructive disease in wheat caused by *Fusarium graminearum* (*F.g*). It infects during the flowering stage favored by warm and highly humid climates. In order to understand possible wheat defense mechanism, gene expression analysis in response to *F.g* was undertaken in three genotypes of wheat, Japanese landrace cultivar Nobeokabouzu (highly resistant), Chinese cv. Sumai 3 (resistant) and Australian cv. Gamenya (susceptible). For microarray analysis, 3 and 7 days after inoculation (dai) samples were used in Agilent wheat custom array 4x38k. At 3 dai, the highest number of genes was up-regulated in Nobeokabouzu followed by Sumai 3 and minimum expression in Gamenya. Whereas at 7 dai, Sumai 3 expressed more genes compared to others. Further narrowing down by excluding commonly expressed genes in three genotypes and grouping according to the gene function has identified differentially high expression of genes involved in detoxification process such as multidrug resistant protein, multidrug resistance-associated protein, UDP-glycosyltransferase and ABC transporters in Nobeokabouzu at 3 dai. However in Sumai 3 many defense-related genes such as peroxidase, proteases and genes involved in plant cell wall defense at 7 dai were identified. These findings showed the difference of molecular defense mechanism among the cultivars in response to the pathogen. The complete data was accessed in NCBI GEO database with accession number GSE59721.

**Table:** 

Specifications	
Subject area	Biology
More specific subject area	Plant-pathogen interaction
Organism	*Triticum aestivum* L. (common wheat) and *Fusarium graminearum* (fungus)
Tissue	Wheat-fungus inoculated florets
Time points	3 and 7 days after inoculation (3 and 7 dai)
Array type	Agilent Wheat custom array 4x38k
Data format	Normalized data
Sample source location	Kihara Institute for Biological Research, Yokohama City University, Japan
Data accessibility	Available in GEO database with accession number GSE59721 (wheat)

## 1 Direct link to deposited data

http://www.ncbi.nlm.nih.gov/geo/query/acc.cgi?acc=GSE59721.

## 2 Experimental design, materials and methods

### 2.1 Materials

The experiment was carried out by selecting three wheat genotypes that differ with regard to their disease response against *Fusarium graminearum* (*F. g*) (Nobeokabouzu, Sumai 3 and Gamenya were selected as highly resistant, resistant and susceptible cultivars respectively). The plant materials were grown in glass house condition. At early anthesis time, florets of each spike were inoculated with *F. g* strain ‘H-3’ by pipetting 10 μl of the fungal suspension (1 × 10^5^ macroconidia ml^− 1^). Mock samples were prepared by inoculating 10 μl of distilled water. In order to develop conducing environment for disease development, the inoculated spikes were covered with a plastic bag for 72 h. Temperature and moisture content in the glass house were maintained at 25 °C and 50% respectively. At 3 and 7 days after inoculation (dai), six spikes per genotype/treatment/time point were sampled for RNA extraction. Three biological replications were done for each sample.

### 2.2 Microarray experiment

Total RNA was extracted by using Nucleo Spin RNA plant kit (Macherey-Nagel, Germany) then converted to cRNA and labelled using Low Input Quick Amp Labeling kit (Agilent Technologies) and fluorophore cy3-CTP. Agilent wheat custom array 4x38k (G2514F) was used to measure the gene expression changes among three different genotypes with and without (mock) FHB infection at 3 and 7 dai. In total, 12 samples were hybridized and biologically replicated three times (12 samples × 3 replication = 36 samples). Gene intensities were extracted from the scanned images, and the data were analyzed using Gene spring 12.6 software (Agilent Technologies).

### 2.3 Data analyses

**Fig. 1:**
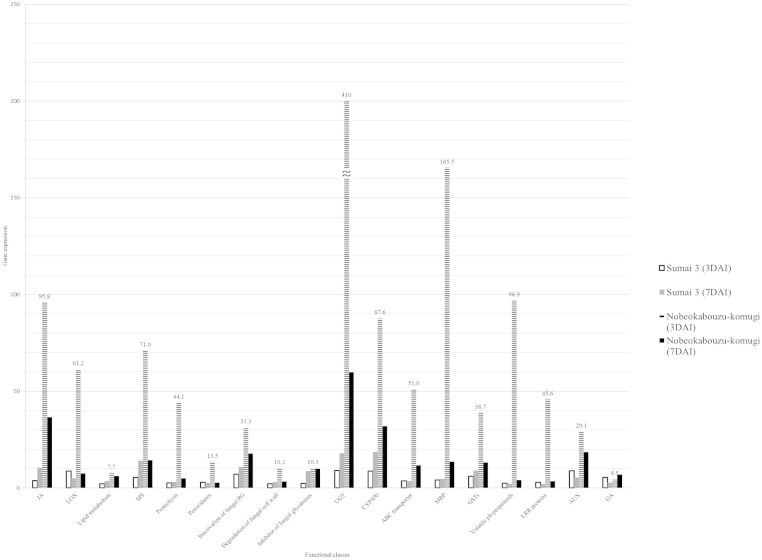
Response of functional gene classes in wheat genotypes. Genes were selected based on their role in resistance and compared in three genotypes in two time points. JA: jasmonate acid related genes, LOX: lipoxygenase genes, SPI: serine protease inhibitor, UGT: UDP-glucosyltransferase, MRP: multidrug resistant protein and GSTs: glutathione-S transferase genes, LRR proteins: leucine rich repeat proteins, AUX: auxin, GA: gibberellic acid, dai: days after inoculation.

**Fig. 2:**
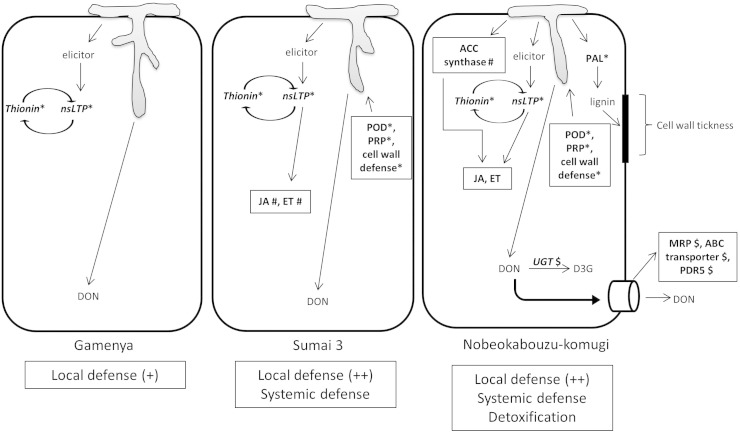
Disease reaction model of three genotypes of wheat. *Local defense; ^#^Systemic defense; ^$^Detoxification; nsLTP: non-specific lipid transporter protein; POD: peroxidase; PRP: pathogenesis related proteins; JA: jasmonic acid; ET: ethylene; PAL: phenylalanine ammonia lyase; ACC synthase: 1-aminocyclopropane-a-carboxylate synthase; MRP: multidrug resistance-associated protein; PDR5: pleiotropic drug resistance protein 5; UGT: UDP-glycosyltransferase; DON: deoxynivalenol; D3G: deoxynivalenol-3-glycoside.

Genome wide gene expression analyses of three genotypes were carried out in a systematic manner. After normalization and statistical analysis, the data were grouped by Venn diagram to categorize the up-regulated genes. The groups were made into three categories, a) common *F. g* responsive genes in wheat genotypes, b) genotypic-specific *F. g* responsive genes for susceptible, resistant and highly resistant wheat genotypes in specific time point (Table 1, Supplementary Table 1) and c) FHB resistance-related genes was picked out by selective comparison of resistant and highly resistant genotypes. Further the expressed genes were functionally assigned to 11 different classes based on previous patho-transcriptomic studies [1], [2], [3], [4]: (1) JA- and ET-related genes; (2) cysteine-rich antimicrobial peptides (AMPs) including serine-protease inhibitors; (3) jasmonate-regulated proteins (JRP); (4) GDSL-lipases; (5) proteolysis including serine proteases; (6) peroxidases (POD); (7) genes related to cell wall defense, such as polygalacturonase inhibiting proteins, xylanase inhibitors and glucan endo-1,3-beta-glucosidase precursors; (8) secondary metabolism and detoxification involved genes; (9) miscellaneous defense-related genes, for example disease resistance-responsive family protein, NBS-LRR disease resistance protein; (10) transcription and signaling related genes and (11) hormone (auxin, gibberellins, abscisic acid and salicylic acid) metabolism related genes. In order to pinpoint difference in molecular mechanism among genotypes, genes were categorized into three functional groups. They were (I) systemic defense-related genes, this includes genes which are known to play important role in plant immunity by eliciting systemic resistance such as, JA & ET related genes, JRP, GDSL-lipase and miscellaneous defense-related genes; (II) local defense-related genes, composed of genes which interact directly with the pathogen avoiding fungal spread such as AMPs, POD, proteolysis and genes related to cell wall defense, and (III) detoxification involved genes, in this group includes secondary metabolism and detoxification process involved genes (Fig. 1). Based on gene expression analysis the disease reaction model of wheat against *F. g* was developed (Fig. 2). Specific genes and their possible molecular mechanism related to disease was explained in Ayumi et al. [5].

The following is the supplementary data related to this article.

Supplementary Table 1 Wheat genotypic specific gene expression against *F.g* at 3 and 7 days after inoculation (dai).

## Figures and Tables

**Table 1:** Number of differentially expressed genes in *F.g-*inoculated wheat at 3 and 7 days after inoculation (dai) in three genotypes.

Genotype	Number of genes			
3 dai		7 dai	
Up	Down	Up	Down
Nobeokabouzu	1228	241	107	662
Sumai 3	347	36	593	45
Gamenya	41	23	37	107
