# Supplementary material for: Genome-wide transcriptional profiling of wheat infected with Fusarium graminearum
Source: Genom Data. 2015 Jun 23;5:260–2. doi: 10.1016/j.gdata.2015.06.020 (PMC4584024; doi:10.1016/j.gdata.2015.06.020)
Supplement: Supplementary Table 1 — Wheat genotypic specific gene expression against F.g at 3 and 7 days after inoculation (dai). [file mmc1.pdf]

Supplementary Table 1. Genotype specific 3 and 7 days after inoculation (dai) up-regulated genes

Gamenya 3 dai specific up-regulated genes			
**Gene classes**	**Fold Change**	**Probe ID**	**Annotation**
**Cysteine-rich Antimicrobial peptides (AMPs)**			
Protease inhibitor	10,4	27963	LOC_Os04g44470.1|11974.m09406|protein Alpha-amylase/subtilisin inhibitor, putative, expressed
Non-specific lipid-transfer protein	57,7	2380	LOC_Os06g31070.1|11976.m07649|protein Protease inhibitor/seed storage/LTP family protein
Non-specific lipid-transfer protein	41,9	11751	LOC_Os06g31070.1|11976.m07649|protein Protease inhibitor/seed storage/LTP family protein
Thionin	189,2	23363	LOC_Os06g32550.1|11976.m07793|protein Plant thionin family protein
Thionin	154,7	14475	LOC_Os06g32550.1|11976.m07793|protein Plant thionin family protein
Thionin	95,3	2756	LOC_Os06g32550.1|11976.m07793|protein Plant thionin family protein
**Miscellaneous defense related genes**			
	2,0	1665	LOC_Os07g49470.1|11977.m09155|protein Serine/threonine-protein kinase RLCKVII, putative
**Transcription and signalling**			
	4,1	25648	LOC_Os09g34160.1|11979.m06465|protein NB-ARC domain containing protein, expressed
	2,8	17932	LOC_Os05g34270.1|11975.m07658|protein Protein kinase domain containing protein, expressed
	2,8	36551	LOC_Os01g02350.1|11971.m06877|protein receptor-like kinase ARK1AS, putative, expressed
	2,6	18154	LOC_Os05g33570.2|11975.m27587|protein Pyruvate, phosphate dikinase, chloroplast precursor
LRR proteins	2,9	11540	LOC_Os04g55420.1|11974.m10434|protein Leucine Rich Repeat family protein, expressed
LRR proteins	2,3	26426	LOC_Os04g58700.1|11974.m35524|protein leucine-rich repeat transmembrane protein kinase
LRR proteins	2,2	28581	LOC_Os10g33140.1|11980.m06146|protein Leucine Rich Repeat family protein, expressed
LRR proteins	2,1	28923	LOC_Os07g38800.1|11977.m08125|protein lectin receptor kinase 7, putative, expressed
LRR proteins	4,5	17148	LOC_Os04g51580.1|11974.m10054|protein Leucine Rich Repeat family protein, expressed
**Uncategoriaed**			
	2,0	447	LOC_Os02g07760.1|11972.m06122|protein Succinate semialdehyde dehydrogenase, mitochondrial precursor
	15,6	24059	LOC_Os02g51550.1|11972.m10188|protein expressed protein
	2,5	5475	LOC_Os02g52610.1|11972.m10292|protein Galactoside 2-alpha-L-fucosyltransferase, putative, expressed
	4,0	15083	LOC_Os03g10110.1|11973.m06465|protein Cupin family protein, expressed
	4,0	34024	LOC_Os03g11300.1|11973.m06581|protein expressed protein
	2,1	19270	LOC_Os03g55280.1|11973.m10443|protein aspartate-semialdehyde dehydrogenase family protein, expressed
	2,2	16810	LOC_Os04g02140.1|11974.m05530|protein transposon protein, putative, CACTA, En/Spm sub-class
	2,2	21239	LOC_Os08g31410.1|11978.m07139|protein Sulfate transporter 1.2, putative, expressed
	2,4	30462	LOC_Os09g32976.1|11979.m06389|protein 60S ribosomal protein L7a, putative, expressed
	2,4	13619	LOC_Os12g02570.1|11982.m04258|protein expressed protein
**Unknown**			
	76,4	1515	
	40,5	12457	
	38,8	32668	
	24,4	18536	
	24,2	17603	
	23,0	30187	
	22,7	19439	
	14,3	28964	
	10,0	7093	
	2,9	30387	
	2,8	35241	
	2,8	34817	
	2,3	27410	
	2,0	25781	
	2,0	24218	

Gamenya 7 dai specific up-regulated genes

Gene classes	Fold Change	Probe ID	Annotation
**JA and ET related genes**			
Lipid metabolism	2,7	14806	LOC_Os08g37250.1|11978.m07711|protein Patatin-like phospholipase family protein, expressed
**Cysteine-rich Antimicrobial peptides (AMPs)**			
Thaumatin	5,1	18177	LOC_Os12g43440.1|11982.m08117|protein Thaumatin-like protein precursor, putative, expressed
**Proteolysis**			
	2,4	26783	LOC_Os11g08100.1|11981.m05006|protein Eukaryotic aspartyl protease family protein, expressed
**Peroxidase**			
	9,1	3455	LOC_Os07g48010.1|11977.m09016|protein Peroxidase 2 precursor, putative, expressed
	3,8	32219	LOC_Os07g47990.1|11977.m09014|protein Peroxidase 2 precursor, putative, expressed
	2,7	3083	LOC_Os07g48050.1|11977.m09020|protein Peroxidase 2 precursor, putative, expressed
**Genes related to cell wall defense**			
Degradation of fungal cell wall	9,2	28540	LOC_Os01g71350.1|11971.m13178|protein Glucan endo-1,3-beta-glucosidase GII precursor
**Secondary metabolism and detoxification**			
Glutathione S-transferases	2,5	18255	LOC_Os02g21460.1|11972.m07386|protein glutathione S-transferase, putative, expressed
**Transcription and signalling**			
	2,5	9639	LOC_Os03g43480.1|11973.m09394|protein heterotrimeric G protein protein gamma subunit
	2,3	14184	LOC_Os10g09620.1|11980.m04304|protein Protein kinase domain containing protein, expressed
	2,3	10179	LOC_Os04g51050.1|11974.m10002|protein wall-associated kinase 1, putative, expressed
	2,3	29469	LOC_Os03g17700.1|11973.m07195|protein Mitogen-activated protein kinase homolog 6, putative
	2,1	16335	LOC_Os05g33570.2|11975.m27587|protein Pyruvate, phosphate dikinase, chloroplast precursor
**Regulation**			
	2,1	10916	LOC_Os06g14490.2|11976.m32040|protein calmodulin-binding heat-shock protein, putative, expressed
**Uncategorized**			
	2,1	36125	LOC_Os01g65900.1|11971.m12698|protein GRAS family transcription factor containing protein, expressed
	2,5	7683	LOC_Os01g72570.2|11971.m43381|protein expressed protein
	2,1	24252	LOC_Os02g09490.1|11972.m06246|protein Cinnamyl-alcohol dehydrogenase, putative, expressed
	2,5	33883	LOC_Os02g11830.1|11972.m06432|protein Coatomer beta subunit, putative, expressed
	3,3	22648	LOC_Os02g47090.1|11972.m09744|protein POT family protein, expressed
	2,1	18509	LOC_Os02g56040.1|11972.m10628|protein DnaJ C terminal region family protein, expressed
	3,1	25149	LOC_Os03g07590.1|11973.m06271|protein expressed protein
	5,1	34373	LOC_Os03g46070.1|11973.m09627|protein P21 protein, putative, expressed
	2,3	2635	LOC_Os03g55320.1|11973.m10447|protein protein phosphatase 2C family protein, putative, expressed
	2,4	23228	LOC_Os04g47190.2|11974.m35184|protein Branched-chain-amino-acid aminotransferase 5, chloroplast precursor
	2,1	29618	LOC_Os05g10810.1|11975.m05551|protein MBOAT family protein, expressed
	2,9	10348	LOC_Os06g37364.2|11976.m08270|protein Ent-kaurene oxidase, putative, expressed
	3,4	37824	LOC_Os07g37320.1|11977.m07982|protein Glucose transporter, putative, expressed
	2,3	36137	LOC_Os08g38440.1|11978.m07828|protein C2 domain containing protein, expressed
	2,0	20156	LOC_Os09g37320.1|11979.m06679|protein retrotransposon protein, putative, unclassified
**Unknown**	13,4	33816	
	4,9	8581	
	4,8	36260	
	2,8	23062	
	2,6	6354	
	2,4	4639	
	2,3	15697	

	2,1	1205	

Sumai 3 3 dai specific up-regulated genes			
**Gene classes**	**Fold Change**	**Probe ID**	**Annotation**
**JA and ET related genes**			
Jasmonic acid	3,2	25295	LOC_Os06g11240.1|11976.m05841|protein 12-oxophytodienoate reductase 2, putative, expressed
Jasmonic acid	3,2	31310	LOC_Os07g36830.1|11977.m07933|protein F-box domain containing protein
Lipid metabolic process	2,5	10676	LOC_Os01g19240.1|11971.m08461|protein Integral membrane protein DUF6 containing protein
**Cysteine-rich Antimicrobial peptides (AMPs)**			
Thionin	12,6	30746	LOC_Os04g11130.1|11974.m06360|protein Gamma-thionins family protein, expressed
Thionin	6,8	18008	LOC_Os04g11130.1|11974.m06360|protein Gamma-thionins family protein, expressed
Thionin	3,6	14550	LOC_Os04g11130.1|11974.m06360|protein Gamma-thionins family protein, expressed
Thionin	2,3	26440	LOC_Os03g60840.1|11973.m10972|protein Bowman-Birk serine protease inhibitor family protein, expressed
Thionin	2,3	12683	LOC_Os03g03810.1|11973.m05913|protein Gamma-thionin homolog PPT precursor, putative, expressed
Thionin	2,1	30628	LOC_Os03g03810.1|11973.m05913|protein Gamma-thionin homolog PPT precursor, putative, expressed
**GDSL-lipases**			
	2,3	12415	LOC_Os05g44200.1|11975.m08546|protein GDSL-like Lipase/Acylhydrolase family protein, expressed
	2,4	27606	LOC_Os02g15230.1|11972.m77786|protein GDSL-like Lipase/Acylhydrolase family protein, expressed
	2,3	495	LOC_Os01g52770.1|11971.m11450|protein GDSL-like Lipase/Acylhydrolase family protein, expressed
	12,4	28107	LOC_Os01g46210.1|11971.m10819|protein GDSL-like Lipase/Acylhydrolase family protein
**Proteolysis**			
	3,4	6901	LOC_Os11g08200.1|11981.m05016|protein Aspartic proteinase Asp1 precursor, putative, expressed
	2,8	15627	LOC_Os12g07780.2|11982.m04765|protein Eukaryotic aspartyl protease family protein, expressed
	5,9	19839	LOC_Os09g28830.1|11979.m06034|protein Serine carboxypeptidase family protein
	16,8	33929	LOC_Os07g48650.1|11977.m09076|protein Subtilisin N-terminal Region family protein, expressed
	2,9	8022	LOC_Os07g24050.1|11977.m06683|protein carboxyl-terminal proteinase, putative
	2,9	28676	LOC_Os05g27500.1|11975.m07038|protein carboxyl-terminal peptidase, putative
	2,2	21876	LOC_Os04g58560.2|11974.m79009|protein Peptidase family C54 containing protein, expressed
	2,3	27919	LOC_Os03g31630.1|11973.m08413|protein Subtilase family protein
	11,5	32416	LOC_Os02g17090.1|11972.m06952|protein Subtilisin N-terminal Region family protein, expressed
	2,1	19403	LOC_Os01g24600.1|11971.m08927|protein Papain family cysteine protease containing protein, expressed
**Peroxidases**			
	3,2	25890	LOC_Os07g48050.1|11977.m09020|protein Peroxidase 2 precursor, putative, expressed
	2,4	25882	LOC_Os01g15830.1|11971.m08181|protein Peroxidase 72 precursor, putative, expressed
**Genes related to cell wall defense**			
Inactivation of fungal polygalacturonase			
	2,1	36826	LOC_Os03g03350.1|11973.m05870|protein polygalacturonase, putative, expressed
	2,0	25154	LOC_Os01g07790.1|11971.m07406|protein Polygalacturonase family protein, expressed
Inhibitor of fungal glycanases			
	2,3	12283	LOC_Os10g21110.1|11980.m05130|protein 1,4-beta-xylanase, putative, expressed
	3,7	28231	LOC_Os04g49730.1|11974.m09906|protein pectinesterase inhibitor domain containing protein
	6,7	32935	LOC_Os02g46290.1|11972.m09666|protein pectinesterase inhibitor domain containing protein, expressed
	2,7	33761	LOC_Os02g33130.1|11972.m08406|protein pectinesterase inhibitor domain containing protein, expressed
	3,2	5190	LOC_Os01g21630.1|11971.m08647|protein Pectinesterase, putative, expressed
	3,8	2099	LOC_Os01g02070.1|11971.m06849|protein pectinesterase inhibitor domain containing protein
	3,7	16372	LOC_Os01g02070.1|11971.m06849|protein pectinesterase inhibitor domain containing protein
	2,1	19409	LOC_Os01g02070.1|11971.m06849|protein pectinesterase inhibitor domain containing protein
Degradation of fungal cell wall			
	5,0	25695	LOC_Os05g33140.1|11975.m07546|protein Basic endochitinase 1 precursor, putative, expressed

**Secondary metabolism and detoxification**			
n UDP-glucoronosyl and UDP-glucosyl transferase family	3,7	264	LOC_Os11g25730.1|11981.m06494|protein UDP-glucoronosyl and UDP-glucosyl transferase family protein, expressed
n UDP-glucoronosyl and UDP-glucosyl transferase family	2,8	5148	LOC_Os07g43710.1|11977.m08596|protein glycosyltransferase 5, putative, expressed
n UDP-glucoronosyl and UDP-glucosyl transferase family	3,1	19010	LOC_Os03g14010.3|11973.m34818|protein Glycosyl hydrolase family 10 protein, expressed
ABC transporter	4,2	7284	LOC_Os11g37700.1|11981.m07615|protein ABC transporter, putative, expressed
ABC transporter	2,2	632	LOC_Os08g29570.1|11978.m06961|protein PDR-type ABC transporter 1, putative, expressed
Cytochrome P450s	6,8	8555	LOC_Os02g09290.1|11972.m06226|protein Cytochrome P450 71D10, putative, expressed
MRP	8,3	7020	LOC_Os01g50160.1|11971.m11200|protein multidrug resistance protein 1 homolog, putative, expressed
Volatile phenylpropanoids	2,2	24118	LOC_Os02g41650.2|11972.m33782|protein Phenylalanine ammonia-lyase, putative, expressed
Flavonoids	2,3	7692	LOC_Os11g32650.1|11981.m07166|protein Chalcone synthase, putative, expressed
	2,1	29125	LOC_Os11g32650.1|11981.m07166|protein Chalcone synthase, putative, expressed
**Transcription and signalling**			
	6,1	28159	LOC_Os03g50550.1|11973.m10046|protein Protein kinase domain containing protein
	6,2	9653	LOC_Os03g51990.1|11973.m10178|protein ACT domain containing protein, expressed
	8,8	7445	LOC_Os01g63680.1|11971.m12483|protein Myb-like DNA-binding domain containing protein, expressed
	3,3	11029	LOC_Os01g33350.1|11971.m09649|protein zinc-binding family protein, putative
	2,5	8534	LOC_Os01g06590.3|11971.m42872|protein Zinc finger, C3HC4 type family protein, expressed
	2,1	6527	LOC_Os12g02220.1|11982.m04223|protein Zinc finger, C3HC4 type family protein
	2,0	23366	LOC_Os11g36480.1|11981.m07500|protein Zinc knuckle family protein, expressed
	11,1	6844	LOC_Os02g34320.1|11972.m08521|protein Helix-loop-helix DNA-binding domain containing protein
LRR-proteins	2,7	4163	LOC_Os05g01370.1|11975.m04676|protein Leucine Rich Repeat family protein, expressed
Regulation			
	2,1	10303	LOC_Os01g38980.1|11971.m10178|protein calmodulin-binding family protein, putative, expressed
Hormone			
Auxine	2,3	25067	LOC_Os06g48950.1|11976.m09421|protein Auxin response factor family protein
Gibbellerin	3,8	20017	LOC_Os03g55290.1|11973.m10444|protein Gibberellin-regulated protein 2 precursor, putative, expressed
Abscisic acid	2,6	1378	LOC_Os02g50140.1|11972.m10048|protein ABA-induced protein, putative
Abscisic acid	2,3	8536	LOC_Os01g64000.1|11971.m12515|protein ABA response element binding factor, putative, expressed
**Uncaracterized**	2,7	17874	LOC_Os12g42130.1|11982.m07992|protein TRANSPARENT TESTA 12 protein, putative, expressed
	3,0	26435	LOC_Os12g40760.1|11982.m07859|protein expressed protein
	3,5	5397	LOC_Os12g29370.1|11982.m06751|protein ZmEBE-1 protein, putative
	6,7	30828	LOC_Os12g07140.2|11982.m04703|protein expressed protein
	2,2	28294	LOC_Os11g33120.1|11981.m07213|protein respiratory burst oxidase protein D, putative, expressed
	2,4	22921	LOC_Os11g32080.1|11981.m07110|protein LGC1, putative
	7,1	9598	LOC_Os11g32070.1|11981.m07109|protein LGC1, putative
	2,1	9617	LOC_Os11g32070.1|11981.m07109|protein LGC1, putative
	2,0	33925	LOC_Os11g32070.1|11981.m07109|protein LGC1, putative
	5,0	1504	LOC_Os11g31540.1|11981.m07057|protein BRASSINOSTEROID INSENSITIVE 1-associated receptor kinase 1 precursor
	2,2	34236	LOC_Os11g29190.1|11981.m06830|protein 40S ribosomal protein S5, putative, expressed
	3,5	28119	LOC_Os11g24610.1|11981.m06387|protein 60S ribosomal protein L38, putative, expressed
	2,6	31323	LOC_Os11g17930.1|11981.m05871|protein expressed protein
	10,7	10106	LOC_Os11g13770.1|11981.m05516|protein hypothetical protein
	3,1	5567	LOC_Os11g13720.1|11981.m05511|protein expressed protein
	2,3	15180	LOC_Os11g05730.1|11981.m04769|protein Histone H3, putative, expressed
	2,6	8703	LOC_Os10g39410.1|11980.m06709|protein Histone H4, putative, expressed
	10,7	22147	LOC_Os10g20560.1|11980.m05077|protein hypothetical protein
	3,3	16979	LOC_Os10g20550.1|11980.m05076|protein expressed protein
	4,1	1826	LOC_Os10g17680.1|11980.m04853|protein Profilin A, putative, expressed

4,9	16543	LOC_Os10g14020.1|11980.m04652|protein expressed protein
2,0	15529	LOC_Os10g05990.2|11980.m21829|protein proline-rich protein, putative, expressed
4,7	32630	LOC_Os10g05970.1|11980.m03997|protein proline-rich protein, putative, expressed
4,0	24805	LOC_Os10g05970.1|11980.m03997|protein proline-rich protein, putative, expressed
2,0	32028	LOC_Os10g05970.1|11980.m03997|protein proline-rich protein, putative, expressed
2,0	20860	LOC_Os10g05970.1|11980.m03997|protein proline-rich protein, putative, expressed
2,0	22735	LOC_Os10g05970.1|11980.m03997|protein proline-rich protein, putative, expressed
3,7	24304	LOC_Os09g39410.2|11979.m22201|protein Male sterility protein, expressed
10,7	14083	LOC_Os09g39400.1|11979.m06878|protein Histidine-containing phosphotransfer protein 1, putative, expressed
2,9	2225	LOC_Os09g39400.1|11979.m06878|protein Histidine-containing phosphotransfer protein 1, putative, expressed
3,5	9080	LOC_Os09g37180.1|11979.m06666|protein Transferase family protein, expressed
2,4	26837	LOC_Os09g33940.1|11979.m06443|protein expressed protein
3,2	27352	LOC_Os09g26550.1|11979.m05807|protein PDZ domain family protein, expressed
2,2	9133	LOC_Os09g25850.1|11979.m05737|protein Gl1 protein, putative, expressed
3,2	17475	LOC_Os09g25330.1|11979.m05685|protein NPH3 family protein, expressed
2,1	2768	LOC_Os09g24230.1|11979.m05579|protein haloacid dehalogenase-like hydrolase family protein, putative, expressed
2,8	24070	LOC_Os09g12050.1|11979.m04467|protein transposon protein, putative, CACTA, En/Spm sub-class
2,6	472	LOC_Os09g11790.1|11979.m04441|protein hypothetical protein
6,7	25457	LOC_Os08g44360.1|11978.m08409|protein Male sterility protein, expressed
2,6	15403	LOC_Os08g44350.1|11978.m08408|protein Hpt domain containing protein, expressed
2,2	31141	LOC_Os08g43470.1|11978.m08321|protein ER lumen protein retaining receptor containing protein, expressed
2,4	26429	LOC_Os08g40830.2|11978.m61965|protein Pumilio-family RNA binding repeat containing protein, expressed
2,4	30781	LOC_Os08g39370.1|11978.m07918|protein sodium/dicarboxylate cotransporter, putative, expressed
14,1	29924	LOC_Os08g38730.1|11978.m07857|protein expressed protein
2,1	19031	LOC_Os08g38300.1|11978.m07815|protein Histone H2B, putative, expressed
21959	4,8	LOC_Os08g19420.1|11978.m06012|protein O-methyltransferase family protein, expressed
23121	3,5	LOC_Os08g19420.1|11978.m06012|protein O-methyltransferase family protein, expressed
3,0	34974	LOC_Os08g07720.1|11978.m04910|protein Transferase family protein, expressed
5,4	11820	LOC_Os08g04740.1|11978.m04617|protein expressed protein
12,7	17860	LOC_Os08g04710.1|11978.m04614|protein hypothetical protein
26,4	5091	LOC_Os08g04540.1|11978.m04597|protein Pyridoxal-dependent decarboxylase conserved domain containing protein
2,1	36681	LOC_Os08g04430.1|11978.m26430|protein expressed protein
2,1	22409	LOC_Os08g02230.1|11978.m04367|protein plant-specific FAD-dependent oxidoreductase family protein, expressed
3,2	9347	LOC_Os07g42490.1|11977.m08481|protein Sucrose synthase 3, putative, expressed
2,3	35015	LOC_Os07g42490.1|11977.m08481|protein Sucrose synthase 3, putative, expressed
2,0	3183	LOC_Os07g40730.1|11977.m29002|protein Helicase conserved C-terminal domain containing protein, expressed
2,8	29838	LOC_Os07g39320.1|11977.m08174|protein Homeobox domain containing protein, expressed
3,2	31313	LOC_Os07g34520.2|11977.m29201|protein Isocitrate lyase, putative, expressed
5,3	5347	LOC_Os07g32380.1|11977.m07503|protein Protein phosphatase 2C containing protein, expressed
34,5	15603	LOC_Os07g24000.1|11977.m06678|protein plasma membrane associated protein, putative, expressed
2,1	25001	LOC_Os07g24000.1|11977.m06678|protein plasma membrane associated protein, putative, expressed
2,2	23954	LOC_Os06g49660.1|11976.m09490|protein Transferase family protein, expressed
3,5	79	LOC_Os06g43880.1|11976.m08918|protein homogentisic acid geranylgeranyl transferase, putative, expressed
4,3	17488	LOC_Os06g42620.1|11976.m08793|protein hypothetical protein
3,0	12202	LOC_Os06g41510.1|11976.m08683|protein D-mannose binding lectin family protein
10,8	27624	LOC_Os06g40050.1|11976.m08542|protein Brittle-1 protein, chloroplast precursor, putative, expressed
3,9	404	LOC_Os06g37020.1|11976.m08237|protein expressed protein
3,3	18981	LOC_Os06g36970.1|11976.m08232|protein transposon protein, putative, unclassified
3,6	13516	LOC_Os06g36180.1|11976.m08155|protein amino acid transporter, putative, expressed

4,1	31384	LOC_Os06g30370.1|11976.m07580|protein MOTHER of FT and TF1 protein, putative, expressed
3,5	33835	LOC_Os06g21910.1|11976.m06847|protein Late embryogenesis abundant group 1 family protein, expressed
2,6	10098	LOC_Os06g21910.1|11976.m06847|protein Late embryogenesis abundant group 1 family protein, expressed
9,9	8040	LOC_Os06g17480.1|11976.m06460|protein Histone-like transcription factor and archaeal histone family protein, expressed
5,5	24511	LOC_Os06g11400.1|11976.m05857|protein expressed protein
4,5	17250	LOC_Os06g11400.1|11976.m05857|protein expressed protein
2,1	17661	LOC_Os06g10750.1|11976.m05792|protein nodulin MtN21 family protein, putative, expressed
2,4	1818	LOC_Os06g10650.1|11976.m05783|protein tyrosine specific protein phosphatase family protein, putative, expressed
12,3	15994	LOC_Os06g10600.1|11976.m05778|protein START domain containing protein, expressed
2,1	27550	LOC_Os06g04390.1|11976.m05168|protein expressed protein
2,8	35333	LOC_Os06g04220.1|11976.m05151|protein expressed protein
3,2	14283	LOC_Os06g04030.1|11976.m05134|protein Histone H3, putative, expressed
2,2	16253	LOC_Os06g02470.2|11976.m04980|protein ozone-responsive stress-related protein, putative, expressed
3,9	26895	LOC_Os06g02470.1|11976.m31956|protein ozone-responsive stress-related protein, putative, expressed
7,1	330	LOC_Os05g49240.1|11975.m08994|protein RADIALIS, putative, expressed
6,1	35303	LOC_Os05g47820.1|11975.m08854|protein expressed protein
2,0	30823	LOC_Os05g39530.1|11975.m08131|protein ribonuclease P family protein, putative, expressed
9,2	15885	LOC_Os05g38400.3|11975.m27870|protein AFG3-like protein 2, putative, expressed
2,1	12657	LOC_Os05g33840.1|11975.m07616|protein 1-deoxy-D-xylulose-5-phosphate synthase, chloroplast, putative, expressed
3,0	23024	LOC_Os05g32880.1|11975.m07520|protein Response regulator receiver domain containing protein
3,2	5258	LOC_Os05g30750.4|11975.m27578|protein C2 domain-containing protein, putative, expressed
2,5	14439	LOC_Os05g28210.1|11975.m07108|protein Embryonic abundant protein 1, putative
2,7	35614	LOC_Os05g14170.1|11975.m05829|protein PQQ enzyme repeat-containing protein, putative, expressed
2,1	4406	LOC_Os04g58830.1|11974.m10763|protein Ribosome biogenesis regulatory protein containing protein, expressed
2,2	18564	LOC_Os04g58820.1|11974.m10762|protein uncharacterized plant-specific domain TIGR01568 family protein, expressed
2,9	1081	LOC_Os04g57550.1|11974.m10640|protein amine oxidase, flavin-containing family protein, expressed
8,7	32694	LOC_Os04g56700.1|11974.m10557|protein Naringenin,2-oxoglutarate 3-dioxygenase, putative
2,6	2313	LOC_Os04g54830.1|11974.m10375|protein expressed protein
2,3	3038	LOC_Os04g48400.1|11974.m09776|protein HOTHEAD protein precursor, putative, expressed
2,0	17253	LOC_Os04g43540.1|11974.m09314|protein 60 ribosomal protein L14, putative, expressed
5,6	32764	LOC_Os04g13480.1|11974.m06587|protein Response regulator receiver domain containing protein, expressed
4,9	2927	LOC_Os03g63870.2|11973.m35586|protein expressed protein
6,1	32884	LOC_Os03g63390.1|11973.m11207|protein Chemocyanin precursor, putative, expressed
6,0	37501	LOC_Os03g63390.1|11973.m11207|protein Chemocyanin precursor, putative, expressed
2,2	8412	LOC_Os03g62790.1|11973.m35525|protein Pirin, putative, expressed
3,1	27160	LOC_Os03g59440.1|11973.m10842|protein Dirigent-like protein, expressed
2,4	21702	LOC_Os03g52594.3|11973.m35626|protein NOT2/NOT3/NOT5 family protein, expressed
2,2	13804	LOC_Os03g52594.3|11973.m35626|protein NOT2/NOT3/NOT5 family protein, expressed
2,4	4391	LOC_Os03g51920.1|11973.m10171|protein expressed protein
13,2	22458	LOC_Os03g48780.1|11973.m09883|protein Oxalate oxidase 2 precursor, putative, expressed
2,1	7310	LOC_Os03g44150.1|11973.m09454|protein ornithine aminotransferase, putative, expressed
2,1	12883	LOC_Os03g38020.1|11973.m08904|protein Mps one binder kinase activator-like 1A, putative, expressed
3,1	2473	LOC_Os03g27580.1|11973.m08085|protein hypothetical protein
34,4	34221	LOC_Os03g25350.1|11973.m07879|protein expressed protein
12,3	9414	LOC_Os03g25350.1|11973.m07879|protein expressed protein
8,4	22260	LOC_Os03g25350.1|11973.m07879|protein expressed protein
6,5	12082	LOC_Os03g25350.1|11973.m07879|protein expressed protein
5,9	18178	LOC_Os03g25350.1|11973.m07879|protein expressed protein
5,7	23030	LOC_Os03g25350.1|11973.m07879|protein expressed protein

4,6	22144	LOC_Os03g25350.1|11973.m07879|protein expressed protein
4,2	32896	LOC_Os03g25350.1|11973.m07879|protein expressed protein
2,8	28236	LOC_Os03g25350.1|11973.m07879|protein expressed protein
4,3	6722	LOC_Os03g22470.1|11973.m07649|protein Desiccation-related protein PCC13-62 precursor, putative, expressed
4,2	18135	LOC_Os03g22470.1|11973.m07649|protein Desiccation-related protein PCC13-62 precursor, putative, expressed
2,5	17609	LOC_Os03g22360.1|11973.m07638|protein expressed protein
3,3	8233	LOC_Os03g21260.1|11973.m07532|protein 2,3-bisphosphoglycerate-independent phosphoglycerate mutase, putative
2,1	7566	LOC_Os03g20120.1|11973.m07424|protein galactinol synthase 3, putative, expressed
6,0	22208	LOC_Os03g16940.1|11973.m07126|protein glyoxalase family protein, expressed
2,3	8973	LOC_Os03g15120.1|11973.m06949|protein Imidazole glycerol phosphate synthase hisHF, chloroplast precursor
3,4	23438	LOC_Os03g15080.1|11973.m78851|protein expressed protein
3,1	21260	LOC_Os03g11170.1|11973.m06568|protein Cystatin, putative
3,2	21678	LOC_Os03g05530.1|11973.m06076|protein Integral membrane protein DUF6 containing protein, expressed
3,2	16806	LOC_Os03g04020.1|11973.m05934|protein Rare lipoprotein A like double-psi beta-barrel containing protein, expressed
2,1	12481	LOC_Os03g01270.1|11973.m05676|protein Beta-expansin 1a precursor, putative, expressed
2,2	6451	LOC_Os02g55390.1|11972.m10565|protein 60S ribosomal protein L39, putative
2,1	33084	LOC_Os02g52560.1|11972.m10287|protein Xyloglucan fucosyltransferase family protein, expressed
2,1	16034	LOC_Os02g51500.2|11972.m34032|protein NTGP5, putative, expressed
2,9	9103	LOC_Os02g49920.1|11972.m10026|protein very-long-chain fatty acid condensing enzyme, putative
3,8	5293	LOC_Os02g38840.2|11972.m77828|protein Glucose-6-phosphate 1-dehydrogenase, cytoplasmic isoform, putative, expressed
9,1	22376	LOC_Os02g37700.1|11972.m08858|protein Lecithin:cholesterol acyltransferase family protein, expressed
2,1	26995	LOC_Os02g36950.2|11972.m08782|protein expressed protein
3,4	21774	LOC_Os02g22780.1|11972.m07515|protein T-complex protein 1, delta subunit, putative, expressed
32,3	949	LOC_Os02g15730.1|11972.m06816|protein Chemocyanin precursor, putative, expressed
4,8	30412	LOC_Os02g13540.1|11972.m06600|protein hypothetical protein
5,5	11750	LOC_Os02g13450.1|11972.m06591|protein hypothetical protein
11,6	31180	LOC_Os02g07440.1|11972.m06091|protein hypothetical protein
2,4	22518	LOC_Os02g07440.1|11972.m06091|protein hypothetical protein
2,3	36974	LOC_Os02g04610.1|11972.m05810|protein expressed protein
2,5	6916	LOC_Os02g04490.1|11972.m05798|protein p300/CBP acetyltransferase-related protein 2, putative, expressed
2,9	7088	LOC_Os02g01590.1|11972.m05509|protein Beta-fructofuranosidase 1 precursor, putative, expressed
15,7	13698	LOC_Os01g70850.1|11971.m13130|protein Esterase PIR7B, putative, expressed
3,2	21386	LOC_Os01g68710.1|11971.m12973|protein FK506-binding protein 2-1 precursor, putative, expressed
9,3	36667	LOC_Os01g58660.1|11971.m12008|protein expressed protein
4,5	19857	LOC_Os01g57890.2|11971.m42734|protein START domain containing protein, expressed
5,2	5516	LOC_Os01g57270.1|11971.m11880|protein rust resistance protein, putative, expressed
7,6	35245	LOC_Os01g57250.1|11971.m11878|protein expressed protein
3,3	26322	LOC_Os01g57240.1|11971.m11877|protein Protein ULTRAPETALA2, putative, expressed
2,7	645	LOC_Os01g54870.1|11971.m11648|protein 60S ribosomal protein L18a, putative, expressed
9,5	28612	LOC_Os01g54430.1|11971.m11607|protein Plastocyanin-like domain containing protein
4,8	6203	LOC_Os01g54430.1|11971.m11607|protein Plastocyanin-like domain containing protein
9,2	15003	LOC_Os01g47410.1|11971.m10936|protein Aspartic proteinase oryzasin-1 precursor, putative, expressed
2,5	18183	LOC_Os01g47340.1|11971.m10929|protein NifU-like N-terminal domain containing protein, mitochondrial precursor
7,1	36276	LOC_Os01g46270.1|11971.m10825|protein wax synthase isoform 2, putative, expressed
6,6	33140	LOC_Os01g44260.1|11971.m10683|protein Dihydroflavonol-4-reductase, putative, expressed
4,6	14480	LOC_Os01g44260.1|11971.m10683|protein Dihydroflavonol-4-reductase, putative, expressed
2,4	11294	LOC_Os01g43980.1|11971.m10657|protein AT hook motif-containing protein, putative
2,1	35074	LOC_Os01g43020.1|11971.m10565|protein CTP synthase, putative, expressed
2,3	31179	LOC_Os01g38670.1|11971.m10148|protein Sugar carrier protein C, putative, expressed

	9,4	3683	LOC_Os01g28230.1|11971.m09267|protein hypothetical protein
	4,7	29951	LOC_Os01g26804.1|11971.m09114|protein ZmEBE-1 protein, putative, expressed
	2,6	17936	LOC_Os01g26804.1|11971.m09114|protein ZmEBE-1 protein, putative, expressed
	3,8	24081	LOC_Os01g24460.1|11971.m08914|protein Histone-like transcription factor and archaeal histone family protein, expressed
	20,8	12122	LOC_Os01g23580.1|11971.m08831|protein Pyrophosphate-energized vacuolar membrane proton pump, putative, expressed
	2,0	35953	LOC_Os01g18220.1|11971.m08364|protein expressed protein
	5,4	2277	LOC_Os01g16714.1|11971.m08267|protein Flavin-binding monooxygenase-like family protein, expressed
	2,2	20331	LOC_Os01g04814.4|11971.m42853|protein Vacuolar protein sorting-associated protein VPS4, putative, expressed
**Unknown**	24,3	31711	
	12,7	33242	
	11,4	20002	
	10,8	3050	
	10,8	21971	
	10,5	33852	
	10,2	21918	
	8,7	26107	
	8,7	21857	
	8,0	15371	
	7,9	15537	
	7,8	28993	
	7,7	31983	
	7,6	10397	
	7,6	2735	
	7,5	7081	
	7,4	23914	
	6,9	29799	
	6,8	30859	
	6,6	1517	
	6,3	8081	
	6,1	6505	
	5,9	360	
	5,5	26787	
	5,3	2641	
	5,2	13701	
	5,2	28995	
	4,9	8241	
	4,9	18515	
	4,9	18954	
	4,8	7174	
	4,8	26160	
	4,7	17006	
	4,7	3299	
	4,6	1904	
	4,5	21593	
	4,4	28712	
	4,3	2417	
	4,2	20287	
	4,1	5133	

4,0	9065
4,0	26229
3,8	26796
3,8	5487
3,7	18735
3,7	33981
3,6	7805
3,6	21927
3,5	20814
3,5	26305
3,4	265
3,4	10221
3,4	1686
3,2	4365
3,2	19103
3,2	5082
3,1	19086
3,1	37326
3,1	17389
3,1	5712
3,1	15300
3,1	11245
3,0	6296
3,0	22113
2,9	1645
2,9	22399
2,9	11792
2,9	32866
2,8	16937
2,8	4563
2,7	11696
2,7	11676
2,7	34503
2,7	26908
2,6	15800
2,6	14787
2,6	11415
2,6	35770
2,6	20115
2,6	2038
2,5	623
2,5	14142
2,5	21194
2,5	12834
2,4	18703
2,4	6858
2,4	25279
2,4	6989
2,3	31362

Gene classes	Fold Change	Probe ID	Annotation
	2,3	23534	
	2,3	34093	
	2,3	18349	
	2,3	33126	
	2,3	28401	
	2,2	13274	
	2,2	33624	
	2,2	13366	
	2,2	4846	
	2,2	22888	
	2,1	6914	
	2,1	21909	
	2,1	19141	
	2,1	28786	
	2,1	29660	
	2,1	37610	
	2,0	7808	
	2,0	23271	
	2,0	9144	
	2,0	5071	
	2,0	25465	
	2,0	13982	
	2,0	15713	

Sumai 3 7 dai specific up-regulated genes			
**Gene classes**	**Fold Change**	**Probe ID**	**Annotation**
**JA and ET related genes**			
Fatty acid metabolism & derivatives/Lipid metabolism	2,6	16083	LOC_Os05g49900.1|11975.m09060|protein Fatty acid elongase, putative, expressed
	2,0	8506	LOC_Os03g43880.1|11973.m09429|protein Patatin-like phospholipase family protein, expressed
	2,4	6824	LOC_Os02g48350.1|11972.m09869|protein Diacylglycerol acyltransferase family protein, expressed
	2,2	8259	LOC_Os01g46240.1|11971.m10822|protein lipase class 3 family protein, putative, expressed
Ethylen biosynthesis	2,2	31295	LOC_Os12g16650.1|11982.m05623|protein cyclopropane fatty acid synthase, putative, expressed
	2,1	31128	LOC_Os05g05680.1|11975.m05095|protein 1-aminocyclopropane-1-carboxylate oxidase, putative, expressed
**Cysteine-rich Antimicrobial peptides (AMPs)**			
Jasmonate-regulated proteins (JRP)	10,5	22753	LOC_Os11g39420.1|11981.m07789|protein Jacalin-like lectin domain containing protein
Non-specific lipid transfer protein	6,3	27539	LOC_Os06g31070.1|11976.m07649|protein Protease inhibitor/seed storage/LTP family protein, expressed
	3,9	18237	LOC_Os06g31070.1|11976.m07649|protein Protease inhibitor/seed storage/LTP family protein, expressed
	3,7	16375	LOC_Os06g31070.1|11976.m07649|protein Protease inhibitor/seed storage/LTP family protein, expressed
	3,6	37734	LOC_Os06g31070.1|11976.m07649|protein Protease inhibitor/seed storage/LTP family protein, expressed
	2,8	8345	LOC_Os06g31070.1|11976.m07649|protein Protease inhibitor/seed storage/LTP family protein, expressed
	2,7	17008	LOC_Os06g31070.1|11976.m07649|protein Protease inhibitor/seed storage/LTP family protein, expressed
	2,5	9839	LOC_Os06g31070.1|11976.m07649|protein Protease inhibitor/seed storage/LTP family protein, expressed
	3,5	26101	LOC_Os12g16880.1|11982.m05646|protein Protease inhibitor/seed storage/LTP family protein, expressed
	2,4	14455	LOC_Os06g31070.1|11976.m07649|protein Protease inhibitor/seed storage/LTP family protein, expressed
	2,2	31962	LOC_Os06g31070.1|11976.m07649|protein Protease inhibitor/seed storage/LTP family protein, expressed
	2,1	1078	LOC_Os06g31070.1|11976.m07649|protein Protease inhibitor/seed storage/LTP family protein, expressed
Serine protease inhibitor	2,9	11070	LOC_Os03g60840.1|11973.m10972|protein Bowman-Birk serine protease inhibitor family protein, expressed
	3,0	34570	LOC_Os01g03380.1|11971.m06977|protein Bowman-Birk type bran trypsin inhibitor precursor, putative
	2,6	23915	LOC_Os01g03380.1|11971.m06977|protein Bowman-Birk type bran trypsin inhibitor precursor, putative

Thionin	9,3	11237	LOC_Os03g03810.1|11973.m05913|protein Gamma-thionin homolog PPT precursor, putative, expressed
	6,2	4345	LOC_Os03g03810.1|11973.m05913|protein Gamma-thionin homolog PPT precursor, putative, expressed
	58,9	8436	LOC_Os02g41910.1|11972.m09228|protein Gamma-thionins family protein, expressed
	16,7	31818	LOC_Os06g32550.1|11976.m07793|protein Plant thionin family protein
**GDSL-lpases**			
	3,1	3521	LOC_Os10g33690.1|11980.m06197|protein GDSL-like Lipase/Acylhydrolase family protein, expressed
	3,4	23861	LOC_Os06g05550.1|11976.m05282|protein GDSL-like Lipase/Acylhydrolase family protein, expressed
**Proteolysis**			
	2,9	32253	LOC_Os11g24389.1|11981.m61768|protein Serine carboxypeptidase family protein, expressed
	9,4	5250	LOC_Os01g61690.1|11971.m12294|protein Serine carboxypeptidase II precursor, putative, expressed
	2,0	22875	LOC_Os09g21370.1|11979.m05344|protein Papain family cysteine protease containing protein, expressed
	2,0	21199	LOC_Os09g27930.1|11979.m05944|protein UBiQuitin family member, putative, expressed
	2,4	22983	LOC_Os06g46770.1|11976.m09203|protein polyubiquitin containing 7 ubiquitin monomers, putative, expressed
	2,0	14810	LOC_Os01g67950.1|11971.m12899|protein ubiquitin family protein, putative, expressed
	3,6	2991	LOC_Os01g72210.1|11971.m13256|protein early-responsive to dehydration protein, putative, expressed
**Peroxidase**			
	4,9	37131	LOC_Os10g02070.1|11980.m03630|protein Peroxidase N precursor, putative, expressed
	2,0	8763	LOC_Os07g01410.1|11977.m04621|protein Peroxidase family protein, expressed
	2,6	1474	LOC_Os06g29470.1|11976.m07491|protein Peroxidase 1 precursor, putative, expressed
	3,6	29271	LOC_Os01g73220.1|11971.m13351|protein Peroxidase 12 precursor, putative, expressed
	3,9	32745	LOC_Os01g22370.2|11971.m42991|protein Peroxidase family protein, expressed
**Genes related to cell wall defense**			
Inactivation of fungal polygalacturonase	6,5	17676	LOC_Os03g59330.1|11973.m10831|protein Polygalacturonase family protein, expressed
Inhibition of fungal glycases	4,8	14000	LOC_Os02g54190.1|11972.m10448|protein Pectinesterase, putative, expressed
Degradation of fungal cell walls	2,3	16583	LOC_Os10g17650.1|11980.m04850|protein Glycosyl hydrolase family 1 protein, expressed
	3,4	34987	LOC_Os09g33680.2|11979.m22014|protein Glycosyl hydrolase family 1 protein, expressed
	2,3	6881	LOC_Os09g33680.1|11979.m06417|protein Glycosyl hydrolase family 1 protein, expressed
	2,2	15615	LOC_Os07g46280.3|11977.m29291|protein Glycosyl hydrolase family 1 protein, expressed
	2,1	9014	LOC_Os05g30350.2|11975.m27797|protein Glycosyl hydrolase family 1 protein, expressed
**Secondary metabolism & detoxification**			
Cytochrome P450s	4,3	15876	LOC_Os10g08319.1|11980.m04183|protein Cytochrome P450 family protein, expressed
	2,5	5435	LOC_Os09g28390.1|11979.m05990|protein Cytochrome P450 family protein, expressed
	2,5	8592	LOC_Os08g01490.1|11978.m04297|protein Cytochrome P450 71C4, putative, expressed
	2,8	17133	LOC_Os04g48460.1|11974.m09782|protein Cytochrome P450 family protein, expressed
UDP-glycosyltransferase family			
	2,3	2354	LOC_Os05g45110.1|11975.m08636|protein UDP-glucoronosyl and UDP-glucosyl transferase family protein
Glutathione S-transferase	2,2	4156	LOC_Os03g57200.1|11973.m10639|protein glutathione S-transferase GSTU1, putative, expressed
	2,2	24907	LOC_Os01g72140.1|11971.m13250|protein Glutathione S-transferase, N-terminal domain containing protein
Volatile phenylpropanoids	4,4	2139	LOC_Os05g35290.1|11975.m07759|protein Phenylalanine ammonia-lyase, putative, expressed
	2,4	2172	LOC_Os04g43800.1|11974.m09339|protein Phenylalanine ammonia-lyase, putative, expressed
	2,2	21183	LOC_Os04g43800.1|11974.m09339|protein Phenylalanine ammonia-lyase, putative, expressed
**Miscellaneous defense related genes**			
	16,5	4055	LOC_Os11g14900.3|11981.m28772|protein Thiol protease SEN102 precursor, putative, expressed
	2,2	15605	LOC_Os11g11780.1|11981.m05370|protein serine/threonine protein kinase, putative, expressed
	2,1	15735	LOC_Os05g01270.1|11975.m04668|protein peptidyl-prolyl cis-trans isomerase, cyclophilin-type family protein
	2,2	804	LOC_Os03g21620.1|11973.m07566|protein Serine/threonine-protein kinase AtPK19, putative, expressed
	2,1	2829	LOC_Os03g21620.1|11973.m07566|protein Serine/threonine-protein kinase AtPK19, putative, expressed
**Transcription & signalling**			
	2,2	1283	LOC_Os12g04240.1|11982.m04418|protein EF hand family protein, expressed

	2,6	5932	LOC_Os10g09620.1|11980.m04304|protein Protein kinase domain containing protein, expressed
	2,1	28002	LOC_Os09g03620.1|11979.m03732|protein Protein kinase domain containing protein, expressed
	2,9	35136	LOC_Os08g10080.1|11978.m05142|protein NAC-domain containing protein 21/22, putative, expressed
	2,5	11531	LOC_Os06g51260.1|11976.m09649|protein myb-like DNA-binding domain, SHAQKYF class family protein
	3,4	3530	LOC_Os06g03970.1|11976.m05128|protein Leucine Rich Repeat family protein, expressed
	2,4	20145	LOC_Os05g37060.1|11975.m07887|protein Myb-like DNA-binding domain containing protein, expressed
	3,7	16706	LOC_Os04g42620.1|11974.m09229|protein Leucine Rich Repeat family protein, expressed
	2,2	26080	LOC_Os04g35100.1|11974.m08601|protein MAP3K-like protein kinase, putative, expressed
	2,5	26121	LOC_Os03g57900.1|11973.m10702|protein AN1-like Zinc finger family protein, expressed
	3,0	37483	LOC_Os02g10920.1|11972.m33608|protein Zinc finger protein ZF2, putative, expressed
	2,9	14739	LOC_Os02g10920.1|11972.m33608|protein Zinc finger protein ZF2, putative, expressed
	2,2	9724	LOC_Os01g54350.1|11971.m11599|protein protein kinase, putative, expressed
**Hormone metabolism**			
Auxine	2,4	9564	LOC_Os08g35190.1|11978.m07508|protein Dormancy/auxin associated protein, expressed
Auxine	2,4	27592	LOC_Os01g58860.1|11971.m12027|protein Auxin Efflux Carrier family protein, expressed
**Un categaorized**			
	2,2	18212	LOC_Os12g43840.1|11982.m08156|protein TPR Domain containing protein, expressed
	3,2	2081	LOC_Os12g38140.1|11982.m07600|protein expressed protein
	3,0	27831	LOC_Os12g38140.1|11982.m07600|protein expressed protein
	2,0	33410	LOC_Os12g38140.1|11982.m07600|protein expressed protein
	2,2	13896	LOC_Os12g32200.1|11982.m07026|protein expressed protein
	2,2	19750	LOC_Os12g32190.1|11982.m07025|protein expressed protein
	6,0	16226	LOC_Os12g27830.1|11982.m06599|protein oxidoreductase, short chain dehydrogenase/reductase family protein
	2,0	25550	LOC_Os12g27830.1|11982.m06599|protein oxidoreductase, short chain dehydrogenase/reductase family protein
	2,3	33133	LOC_Os12g27440.1|11982.m06561|protein expressed protein
	21,6	21966	LOC_Os12g25090.2|11982.m56494|protein expressed protein
	4,1	35124	LOC_Os12g25090.2|11982.m56494|protein expressed protein
	5,4	11075	LOC_Os12g24330.1|11982.m06267|protein expressed protein
	4,0	15610	LOC_Os12g16890.1|11982.m05647|protein Prolamin PPROL 17 precursor, putative, expressed
	4,0	19387	LOC_Os12g16890.1|11982.m05647|protein Prolamin PPROL 17 precursor, putative, expressed
	3,9	14927	LOC_Os12g16890.1|11982.m05647|protein Prolamin PPROL 17 precursor, putative, expressed
	3,5	15049	LOC_Os12g16890.1|11982.m05647|protein Prolamin PPROL 17 precursor, putative, expressed
	2,6	14795	LOC_Os12g16890.1|11982.m05647|protein Prolamin PPROL 17 precursor, putative, expressed
	5,4	29004	LOC_Os12g05330.1|11982.m04526|protein expressed protein
	2,5	29939	LOC_Os12g03050.1|11982.m04303|protein No apical meristem protein, expressed
	2,0	14037	LOC_Os12g01530.1|11982.m04157|protein Ferritin 1, chloroplast precursor, putative, expressed
	2,2	8357	LOC_Os11g44750.1|11981.m08308|protein retrotransposon protein, putative, unclassified
	2,1	10315	LOC_Os11g41600.2|11981.m28854|protein expressed protein
	2,1	194	LOC_Os11g37340.1|11981.m07581|protein F-box domain containing protein
	66,6	28925	LOC_Os11g37270.1|11981.m07574|protein expressed protein
	40,4	36747	LOC_Os11g37270.1|11981.m07574|protein expressed protein
	7,0	28451	LOC_Os11g37270.1|11981.m07574|protein expressed protein
	3,9	14291	LOC_Os11g20090.1|11981.m06084|protein O-methyltransferase family protein, expressed
	2,3	31183	LOC_Os11g17930.1|11981.m05871|protein expressed protein
	2,2	27511	LOC_Os11g10590.1|11981.m05254|protein hypothetical protein
	3,3	31548	LOC_Os11g10510.1|11981.m05246|protein Alcohol dehydrogenase 2, putative, expressed
	5,2	567	LOC_Os11g05562.2|11981.m61755|protein 40S ribosomal protein S25, putative, expressed
	2,6	14555	LOC_Os10g41020.1|11980.m06867|protein oxidoreductase, 2OG-Fe oxygenase family protein, expressed
	2,5	26057	LOC_Os10g40600.1|11980.m06828|protein Nitrate/chlorate transporter, putative, expressed

3,1	27060	LOC_Os10g39780.1|11980.m06749|protein protein phosphatase 2C homolog, putative, expressed
2,3	19281	LOC_Os10g39780.1|11980.m06749|protein protein phosphatase 2C homolog, putative, expressed
3,7	13862	LOC_Os10g35050.1|11980.m06328|protein Aquaporin TIP3.1, putative, expressed
3,6	17419	LOC_Os10g26730.1|11980.m05612|protein exonuclease family protein, putative, expressed
2,2	21818	LOC_Os10g22950.1|11980.m05301|protein anther ethylene-upregulated protein ER1, putative, expressed
2,3	11308	LOC_Os10g22570.1|11980.m05264|protein Cellulase containing protein, expressed
3,6	10145	LOC_Os10g09850.1|11980.m04325|protein 39 kDa EF-Hand containing protein, putative, expressed
2,4	34781	LOC_Os10g07229.1|11980.m04111|protein Alcohol dehydrogenase 2, putative, expressed
2,4	27222	LOC_Os10g04110.1|11980.m03828|protein NB-ARC domain containing protein, expressed
3,1	11514	LOC_Os10g02840.1|11980.m03701|protein O-methyltransferase family protein, expressed
2,4	992	LOC_Os09g37600.1|11979.m06707|protein LysM domain containing protein, expressed
2,1	936	LOC_Os09g33450.2|11979.m50233|protein DHHC zinc finger domain containing protein, expressed
6,7	31266	LOC_Os09g32670.1|11979.m06354|protein NAD dependent epimerase/dehydratase family protein, expressed
7,3	18401	LOC_Os09g32260.1|11979.m06315|protein No apical meristem protein, expressed
3,3	9028	LOC_Os09g32260.1|11979.m06315|protein No apical meristem protein, expressed
2,2	5063	LOC_Os09g31200.1|11979.m06235|protein Multiple stress-responsive zinc-finger protein ISAP1
5,6	26454	LOC_Os09g26380.1|11979.m05790|protein Alanine aminotransferase 2, putative, expressed
2,2	29685	LOC_Os09g25460.1|11979.m05698|protein Transferase family protein, expressed
13,7	16092	LOC_Os09g20440.1|11979.m05251|protein succinate dehydrogenase and fumarate reductase iron-sulfur protein
2,0	8171	LOC_Os09g20440.1|11979.m05251|protein succinate dehydrogenase and fumarate reductase iron-sulfur protein
2,7	17472	LOC_Os09g20000.1|11979.m05207|protein heavy-metal-associated domain-containing protein
2,1	30850	LOC_Os09g06464.1|11979.m03916|protein CCT motif family protein, expressed
3,9	25029	LOC_Os09g04310.1|11979.m03800|protein expressed protein
2,9	10687	LOC_Os09g04050.1|11979.m03774|protein NAD dependent epimerase/dehydratase family protein, expressed
3,5	5677	LOC_Os08g45110.1|11978.m08481|protein AP2 domain containing protein, expressed
2,2	1539	LOC_Os08g39720.1|11978.m07952|protein retrotransposon protein, putative, unclassified
2,2	28928	LOC_Os08g39150.1|11978.m07897|protein expressed protein
3,3	33363	LOC_Os08g36440.1|11978.m26709|protein HVA22 protein, putative, expressed
2,6	19468	LOC_Os08g32120.1|11978.m07206|protein expressed protein
2,5	21986	LOC_Os08g31170.1|11978.m07115|protein C1-like domain containing protein
6,1	25088	LOC_Os08g30020.3|11978.m26679|protein expressed protein
8,4	35574	LOC_Os08g08980.1|11978.m05033|protein Germin-like protein subfamily 1 member 7 precursor
8,9	12927	LOC_Os08g05970.1|11978.m04738|protein expressed protein
2,3	23148	LOC_Os08g01390.1|11978.m04288|protein Phosphatidylinositol-4-phosphate 5-Kinase family protein
2,3	9208	LOC_Os07g45350.5|11977.m29011|protein expressed protein
10,4	25182	LOC_Os07g44430.2|11977.m29285|protein peroxiredoxin, putative, expressed
8,1	23615	LOC_Os07g44430.2|11977.m29285|protein peroxiredoxin, putative, expressed
2,1	3991	LOC_Os07g43970.1|11977.m08620|protein calmodulin-binding family protein, putative, expressed
2,0	10771	LOC_Os07g41150.1|11977.m08351|protein glycerophosphoryl diester phosphodiesterase family protein
4,7	10277	LOC_Os07g40974.1|11977.m08336|protein NAD dependent epimerase/dehydratase family protein, expressed
2,5	12991	LOC_Os07g40860.1|11977.m08326|protein expressed protein
3,0	26974	LOC_Os07g39350.1|11977.m08177|protein Sugar transporter family protein, expressed
2,2	35665	LOC_Os07g39350.1|11977.m08177|protein Sugar transporter family protein, expressed
2,3	31900	LOC_Os07g38840.2|11977.m08129|protein pre-mRNA processing protein PRP39, putative, expressed
2,4	28319	LOC_Os07g38120.1|11977.m08061|protein Calcium-dependent protein kinase, isoform AK1, putative, expressed
3,2	33850	LOC_Os07g35940.1|11977.m07847|protein Beta-amylase, putative
2,7	4827	LOC_Os07g35940.1|11977.m07847|protein Beta-amylase, putative
7,6	28399	LOC_Os07g34570.2|11977.m28985|protein Thiazole biosynthetic enzyme 1-1, chloroplast precursor
11,2	19267	LOC_Os07g34570.1|11977.m07714|protein Thiazole biosynthetic enzyme 1-1, chloroplast precursor

2,1	12292	LOC_Os07g32570.1|11977.m07520|protein 5'-adenylylsulfate reductase 2, chloroplast precursor
2,6	21445	LOC_Os07g20340.2|11977.m29133|protein heavy metal-associated domain containing protein, expressed
2,3	2629	LOC_Os07g20340.2|11977.m29133|protein heavy metal-associated domain containing protein, expressed
5,7	18736	LOC_Os07g12080.1|11977.m05661|protein Trypsin/factor XIIA inhibitor precursor, putative, expressed
4,3	18049	LOC_Os07g12080.1|11977.m05661|protein Trypsin/factor XIIA inhibitor precursor, putative, expressed
2,9	36205	LOC_Os07g12080.1|11977.m05661|protein Trypsin/factor XIIA inhibitor precursor, putative, expressed
2,8	1863	LOC_Os07g12080.1|11977.m05661|protein Trypsin/factor XIIA inhibitor precursor, putative, expressed
2,4	26942	LOC_Os07g12080.1|11977.m05661|protein Trypsin/factor XIIA inhibitor precursor, putative, expressed
2,4	37079	LOC_Os07g12080.1|11977.m05661|protein Trypsin/factor XIIA inhibitor precursor, putative, expressed
2,4	13474	LOC_Os07g12080.1|11977.m05661|protein Trypsin/factor XIIA inhibitor precursor, putative, expressed
2,2	13020	LOC_Os07g12080.1|11977.m05661|protein Trypsin/factor XIIA inhibitor precursor, putative, expressed
2,1	35787	LOC_Os07g12080.1|11977.m05661|protein Trypsin/factor XIIA inhibitor precursor, putative, expressed
2,0	14254	LOC_Os07g12080.1|11977.m05661|protein Trypsin/factor XIIA inhibitor precursor, putative, expressed
6,2	7333	LOC_Os07g11510.1|11977.m05605|protein Seed allergenic protein RA5 precursor, putative, expressed
2,6	31256	LOC_Os07g11360.1|11977.m05591|protein Seed allergenic protein RA17 precursor, putative, expressed
2,5	11973	LOC_Os07g11330.1|11977.m05588|protein Seed allergenic protein RA14 precursor, putative, expressed
2,1	9049	LOC_Os07g11330.1|11977.m05588|protein Seed allergenic protein RA14 precursor, putative, expressed
2,1	2567	LOC_Os07g10890.1|11977.m29100|protein HALF-1 transcription factor, putative, expressed
2,1	32206	LOC_Os07g07470.1|11977.m05206|protein 2-oxoisovalerate dehydrogenase beta subunit, mitochondrial precursor
3,5	21666	LOC_Os07g07410.1|11977.m05200|protein oxidoreductase, 2OG-Fe oxygenase family protein, expressed
2,3	14590	LOC_Os06g51390.2|11976.m32211|protein expressed protein
2,9	11793	LOC_Os06g51084.4|11976.m32274|protein 1,4-alpha-glucan branching enzyme, chloroplast precursor
2,8	19630	LOC_Os06g51084.4|11976.m32274|protein 1,4-alpha-glucan branching enzyme, chloroplast precursor
2,1	21038	LOC_Os06g46950.1|11976.m09221|protein EF-hand Ca2+-binding protein CCD1, putative, expressed
3,3	16752	LOC_Os06g46900.1|11976.m09216|protein phosphosulfolactate synthase-related protein, putative, expressed
2,0	3393	LOC_Os06g45330.1|11976.m09060|protein Endonuclease V, putative, expressed
25,2	11608	LOC_Os06g44144.1|11976.m31814|protein expressed protein
6,1	24833	LOC_Os06g44144.1|11976.m31814|protein expressed protein
3,3	3649	LOC_Os06g44144.1|11976.m31814|protein expressed protein
6,3	2786	LOC_Os06g41810.1|11976.m08713|protein NAD dependent epimerase/dehydratase family protein, expressed
5,1	28508	LOC_Os06g36920.1|11976.m08227|protein thromboxane-A synthase, putative, expressed
2,2	10640	LOC_Os06g36560.2|11976.m32098|protein Inositol oxygenase, putative, expressed
2,1	11949	LOC_Os06g35900.1|11976.m08127|protein BES1/BZR1 homolog protein 4, putative, expressed
2,2	36242	LOC_Os06g22390.1|11976.m06893|protein expressed protein
4,6	6309	LOC_Os06g21910.1|11976.m06847|protein Late embryogenesis abundant group 1 family protein, expressed
8,9	35414	LOC_Os06g15420.1|11976.m06255|protein Asparagine synthetase, putative, expressed
7,6	17062	LOC_Os06g12370.1|11976.m05954|protein Cell division protein ftsH homolog, putative, expressed
2,0	32471	LOC_Os06g11310.1|11976.m05848|protein Plastocyanin-like domain containing protein, expressed
10,7	21698	LOC_Os05g50110.1|11975.m09080|protein Oleosin family protein, expressed
29,0	35488	LOC_Os05g49440.2|11975.m09014|protein secreted protein, putative, expressed
2,7	33548	LOC_Os05g48030.2|11975.m27955|protein Anti-silencing protein 1, putative, expressed
8,8	23747	LOC_Os05g46480.1|11975.m08771|protein Late embryogenesis abundant protein, expressed
6,1	31495	LOC_Os05g46480.1|11975.m08771|protein Late embryogenesis abundant protein, expressed
2,5	3011	LOC_Os05g45450.1|11975.m08670|protein nuclear protein, putative, expressed
2,2	3070	LOC_Os05g44340.1|11975.m08560|protein Heat shock protein 101, putative, expressed
2,0	20843	LOC_Os05g43590.1|11975.m08486|protein Serpin family protein
2,1	18792	LOC_Os05g43370.1|11975.m08465|protein tRNA pseudouridine synthase D family protein, expressed
5,5	29907	LOC_Os05g41970.1|11975.m08371|protein 19 kDa globulin precursor, putative, expressed
3,9	29119	LOC_Os05g41970.1|11975.m08371|protein 19 kDa globulin precursor, putative, expressed

2,9	13047	LOC_Os05g41970.1|11975.m08371|protein 19 kDa globulin precursor, putative, expressed
2,7	25057	LOC_Os05g41970.1|11975.m08371|protein 19 kDa globulin precursor, putative, expressed
2,7	30500	LOC_Os05g41970.1|11975.m08371|protein 19 kDa globulin precursor, putative, expressed
2,1	15167	LOC_Os05g41000.1|11975.m08276|protein Endonuclease/Exonuclease/phosphatase family protein, expressed
10,1	16102	LOC_Os05g39690.1|11975.m08147|protein Aldose reductase, putative, expressed
3,4	17553	LOC_Os05g39250.1|11975.m08104|protein expressed protein
2,3	31717	LOC_Os05g38940.1|11975.m08073|protein expressed protein
2,1	17698	LOC_Os05g36270.1|11975.m07858|protein Fructose-1,6-bisphosphatase, cytosolic, putative, expressed
6,1	10059	LOC_Os05g31020.1|11975.m07337|protein Eukaryotic peptide chain release factor subunit 1-2
2,4	30577	LOC_Os05g29810.1|11975.m07218|protein AP2 domain containing protein, expressed
2,8	20362	LOC_Os05g07650.1|11975.m05284|protein Endomembrane protein 70 containing protein, expressed
10,0	8483	LOC_Os05g06310.1|11975.m05158|protein 60S ribosomal protein L18, putative, expressed
4,7	12003	LOC_Os05g03130.1|11975.m04847|protein expressed protein
2,1	5845	LOC_Os04g58710.1|11974.m10751|protein AMP-binding enzyme family protein, expressed
2,4	16699	LOC_Os04g57200.1|11974.m35503|protein heavy metal-associated domain containing protein, expressed
2,1	25987	LOC_Os04g55650.2|11974.m78923|protein Low-temperature-induced cysteine proteinase precursor
2,5	923	LOC_Os04g54280.1|11974.m10320|protein retrotransposon protein, putative, Ty1-copia subclass
2,1	28371	LOC_Os04g50120.1|11974.m09944|protein expressed protein
2,3	21573	LOC_Os04g48270.2|11974.m78983|protein F-box family protein, putative, expressed
3,3	33471	LOC_Os04g46200.1|11974.m09571|protein Oleosin 16 kDa, putative, expressed
3,3	13024	LOC_Os04g44980.1|11974.m09452|protein oxidoreductase, short chain dehydrogenase/reductase family protein
2,1	7640	LOC_Os04g44950.1|11974.m09449|protein oxidoreductase, short chain dehydrogenase/reductase family protein
2,2	10549	LOC_Os04g44670.1|11974.m09426|protein AP2 domain containing protein
2,1	15435	LOC_Os04g44510.1|11974.m09410|protein GRAM domain-containing protein, putative, expressed
2,8	8957	LOC_Os04g44470.1|11974.m09406|protein Alpha-amylase/subtilisin inhibitor, putative, expressed
2,8	15952	LOC_Os04g44470.1|11974.m09406|protein Alpha-amylase/subtilisin inhibitor, putative, expressed
11,6	2998	LOC_Os04g44120.1|11974.m09370|protein expressed protein
4,2	36847	LOC_Os04g43200.1|11974.m09284|protein caleosin 2, putative, expressed
3,5	33173	LOC_Os04g43200.1|11974.m09284|protein caleosin 2, putative, expressed
7,4	30282	LOC_Os04g43170.1|11974.m09281|protein Caleosin related protein, expressed
2,1	31923	LOC_Os04g42930.1|11974.m09258|protein Glutaredoxin, putative, expressed
2,0	25694	LOC_Os04g40910.1|11974.m09071|protein F-box domain containing protein, expressed
2,0	17226	LOC_Os04g40540.2|11974.m78963|protein Protein-L-isoaspartate O-methyltransferase, putative, expressed
4,3	24100	LOC_Os04g39700.1|11974.m08956|protein 60S ribosomal protein L6, putative, expressed
2,1	3667	LOC_Os04g37760.1|11974.m08765|protein expressed protein
2,8	18329	LOC_Os04g37460.1|11974.m08735|protein Glutamate decarboxylase, putative, expressed
2,3	14820	LOC_Os04g36058.2|11974.m35574|protein expressed protein
2,3	22075	LOC_Os04g35840.1|11974.m08673|protein T-complex protein 11 containing protein, expressed
2,3	25746	LOC_Os04g35280.1|11974.m08618|protein Neutral/alkaline invertase, putative, expressed
4,0	7777	LOC_Os04g33720.1|11974.m08467|protein Beta-fructofuranosidase, insoluble isoenzyme 3 precursor
3,3	31951	LOC_Os04g32070.1|11974.m08305|protein oxidoreductase, short chain dehydrogenase/reductase family protein
3,0	21485	LOC_Os04g01740.1|11974.m05490|protein Heat shock protein 82, putative, expressed
2,2	19473	LOC_Os03g61730.1|11973.m11054|protein DnaJ domain containing protein, expressed
2,6	26171	LOC_Os03g59300.2|11973.m34946|protein expressed protein
2,5	22429	LOC_Os03g59300.2|11973.m34946|protein expressed protein
2,8	19532	LOC_Os03g58170.2|11973.m34944|protein Stem-specific protein TSJT1, putative, expressed
90,3	14852	LOC_Os03g57960.1|11973.m10708|protein Cupin family protein, expressed
31,2	8668	LOC_Os03g57960.1|11973.m10708|protein Cupin family protein, expressed
2,2	2929	LOC_Os03g56250.1|11973.m10534|protein wound and phytochrome signaling involved receptor like kinase

2,3	29781	LOC_Os03g55180.1|11973.m10433|protein expressed protein
3,1	10566	LOC_Os03g53900.1|11973.m78892|protein universal stress protein family protein, expressed
4,2	19348	LOC_Os03g53400.1|11973.m10310|protein Uncharacterized protein family UPF0005 containing protein
4,0	12333	LOC_Os03g53310.1|11973.m10303|protein emp24/gp25L/p24 family protein, expressed
4,9	24848	LOC_Os03g51470.1|11973.m10125|protein expressed protein
4,0	34501	LOC_Os03g51470.1|11973.m10125|protein expressed protein
2,1	19829	LOC_Os03g51430.1|11973.m10122|protein CRAL/TRIO domain containing protein, expressed
2,4	26785	LOC_Os03g50490.1|11973.m10040|protein Glutamine synthetase root isozyme 2, putative, expressed
2,4	37486	LOC_Os03g50490.1|11973.m10040|protein Glutamine synthetase root isozyme 2, putative, expressed
2,3	22079	LOC_Os03g50160.1|11973.m10011|protein Chemocyanin precursor, putative, expressed
50,4	36194	LOC_Os03g46100.1|11973.m09630|protein Cupin family protein, expressed
38,8	28125	LOC_Os03g46100.1|11973.m09630|protein Cupin family protein, expressed
35,7	77	LOC_Os03g46100.1|11973.m09630|protein Cupin family protein, expressed
32,9	37190	LOC_Os03g46100.1|11973.m09630|protein Cupin family protein, expressed
28,0	3941	LOC_Os03g46100.1|11973.m09630|protein Cupin family protein, expressed
2,0	18219	LOC_Os03g43720.5|11973.m35324|protein major facilitator superfamily protein, expressed
5,1	33039	LOC_Os03g41438.1|11973.m09209|protein Protein Z, putative, expressed
5,0	23737	LOC_Os03g41438.1|11973.m09209|protein Protein Z, putative, expressed
4,8	8860	LOC_Os03g41438.1|11973.m09209|protein Protein Z, putative, expressed
6,1	2668	LOC_Os03g41419.1|11973.m09208|protein Serpin family protein, expressed
5,8	2452	LOC_Os03g41419.1|11973.m09208|protein Serpin family protein, expressed
4,9	12692	LOC_Os03g41419.1|11973.m09208|protein Serpin family protein, expressed
4,3	1323	LOC_Os03g41419.1|11973.m09208|protein Serpin family protein, expressed
2,1	34758	LOC_Os03g40830.1|11973.m09156|protein cucumisin-like serine protease, putative, expressed
2,2	8940	LOC_Os03g40194.1|11973.m09103|protein DSBA-like thioredoxin domain containing protein, expressed
5,2	36296	LOC_Os03g26490.1|11973.m07985|protein expressed protein
2,0	2547	LOC_Os03g22620.1|11973.m07663|protein Terpene synthase family, metal binding domain containing protein
2,0	35960	LOC_Os03g21470.1|11973.m07551|protein 60S ribosomal protein L10, putative, expressed
4,9	29348	LOC_Os03g20680.1|11973.m07475|protein Late embryogenesis abundant protein, expressed
3,9	28646	LOC_Os03g20680.1|11973.m07475|protein Late embryogenesis abundant protein, expressed
3,5	9635	LOC_Os03g20680.1|11973.m07475|protein Late embryogenesis abundant protein, expressed
7,0	31641	LOC_Os03g19290.1|11973.m07346|protein mitochondrial import inner membrane translocase subunit Tim17/Tim22/Tim23 family protein
2,3	35639	LOC_Os03g19220.1|11973.m07339|protein negatively light-regulated protein, putative, expressed
2,9	27518	LOC_Os03g18454.2|11973.m35570|protein Gamma interferon inducible lysosomal thiol reductase family protein
2,2	12513	LOC_Os03g18454.2|11973.m35570|protein Gamma interferon inducible lysosomal thiol reductase family protein
2,3	16891	LOC_Os03g17870.1|11973.m07212|protein Metallothionein-like protein 1, putative, expressed
2,0	19118	LOC_Os03g17870.1|11973.m07212|protein Metallothionein-like protein 1, putative, expressed
2,6	17584	LOC_Os03g17730.1|11973.m07198|protein prephenate dehydratase family protein, expressed
2,1	12877	LOC_Os03g17470.1|11973.m07174|protein IN2-1 protein, putative, expressed
7,7	36971	LOC_Os03g16040.1|11973.m07041|protein 17.4 kDa class I heat shock protein, putative, expressed
2,1	17772	LOC_Os03g15960.1|11973.m07033|protein 17.4 kDa class I heat shock protein, putative, expressed
3,3	34134	LOC_Os03g15050.4|11973.m35128|protein Phosphoenolpyruvate carboxykinase, putative, expressed
3,3	30444	LOC_Os03g11910.1|11973.m06643|protein dnaK protein, expressed
2,9	2872	LOC_Os03g11910.1|11973.m06643|protein dnaK protein, expressed
6,4	15083	LOC_Os03g10110.1|11973.m06465|protein Cupin family protein, expressed
2,4	17942	LOC_Os03g09250.1|11973.m06430|protein Inositol-3-phosphate synthase, putative, expressed
2,1	36003	LOC_Os03g09250.1|11973.m06430|protein Inositol-3-phosphate synthase, putative, expressed
2,5	12140	LOC_Os03g09020.1|11973.m06408|protein oxidoreductase, zinc-binding dehydrogenase family protein, expressed
2,1	10628	LOC_Os03g08500.1|11973.m06358|protein AP2 domain containing protein, expressed

3,6	33694	LOC_Os03g08060.2|11973.m78809|protein Elongation factor 1-alpha, putative, expressed
2,1	37714	LOC_Os03g08060.2|11973.m78809|protein Elongation factor 1-alpha, putative, expressed
2,2	6200	LOC_Os03g06370.2|11973.m35593|protein expressed protein
17,9	32695	LOC_Os03g06360.1|11973.m06156|protein Seed maturation protein, expressed
2,2	13249	LOC_Os03g06180.1|11973.m06138|protein expressed protein
2,1	19305	LOC_Os03g02840.1|11973.m05823|protein Remorin, C-terminal region family protein, expressed
2,0	17644	LOC_Os02g58480.1|11972.m10869|protein Sucrose synthase 2, putative, expressed
2,6	123	LOC_Os02g58260.1|11972.m10847|protein metallo-beta-lactamase family protein, putative, expressed
2,2	32639	LOC_Os02g55560.1|11972.m10582|protein DNA-binding protein phosphatase 2C, putative, expressed
2,1	25076	LOC_Os02g55560.1|11972.m10582|protein DNA-binding protein phosphatase 2C, putative, expressed
2,7	27983	LOC_Os02g55070.1|11972.m10533|protein hypothetical protein
2,1	5284	LOC_Os02g48010.1|11972.m09835|protein nuclear matrix constituent protein 1, putative, expressed
3,0	12763	LOC_Os02g47950.1|11972.m09829|protein expressed protein
2,6	29956	LOC_Os02g47840.2|11972.m09818|protein expressed protein
2,0	17973	LOC_Os02g47570.1|11972.m09791|protein PAP2 superfamily protein, expressed
2,2	22943	LOC_Os02g47210.1|11972.m09756|protein amino acid permease family protein, putative, expressed
2,1	27379	LOC_Os02g42640.1|11972.m09299|protein expressed protein
2,6	1611	LOC_Os02g40730.1|11972.m09159|protein Ammonium transporter 1, member 2, putative, expressed
2,2	36954	LOC_Os02g39790.1|11972.m09065|protein S-adenosylmethionine decarboxylase proenzyme, putative, expressed
3,5	17272	LOC_Os02g37070.1|11972.m08795|protein hypothetical protein
3,8	22358	LOC_Os02g37040.1|11972.m08792|protein POT family protein, expressed
2,0	17053	LOC_Os02g35690.1|11972.m08657|protein expressed protein
2,2	32423	LOC_Os02g33230.1|11972.m08416|protein expressed protein
2,3	7372	LOC_Os02g32110.1|11972.m08304|protein exostosin family protein, putative, expressed
3,3	3752	LOC_Os02g30470.1|11972.m08190|protein expressed protein
2,2	14798	LOC_Os02g30470.1|11972.m08190|protein expressed protein
2,1	33185	LOC_Os02g30470.1|11972.m08190|protein expressed protein
3712,2	5385	LOC_Os02g30050.1|11972.m08148|protein 60S ribosomal protein L35, putative, expressed
2,0	31730	LOC_Os02g19924.1|11972.m07233|protein tyrosine/nicotianamine aminotransferases family protein, expressed
5,6	16294	LOC_Os02g18640.1|11972.m07107|protein F-box domain containing protein, expressed
2,8	21226	LOC_Os02g15930.1|11972.m06836|protein expressed protein
9,1	4347	LOC_Os02g15250.1|11972.m06768|protein Late embryogenesis abundant protein, expressed
8,8	29571	LOC_Os02g04780.1|11972.m05826|protein expressed protein
2,3	20237	LOC_Os02g04700.2|11972.m77728|protein Aspartyl-tRNA synthetase, putative, expressed
3,1	3240	LOC_Os02g04130.1|11972.m05762|protein expressed protein
2,0	27869	LOC_Os02g03280.2|11972.m33327|protein Bax inhibitor-1, putative, expressed
2,1	31025	LOC_Os02g02930.1|11972.m05643|protein Terpene synthase family, metal binding domain containing protein, expressed
2,3	3750	LOC_Os02g02630.1|11972.m05612|protein hypothetical protein
2,2	22972	LOC_Os02g02400.1|11972.m05589|protein Catalase isozyme A, putative, expressed
2,2	34982	LOC_Os02g02400.1|11972.m05589|protein Catalase isozyme A, putative, expressed
2,0	4995	LOC_Os02g02400.1|11972.m05589|protein Catalase isozyme A, putative, expressed
3,9	34736	LOC_Os02g02190.1|11972.m05569|protein High affinity nitrate transporter, putative, expressed
2,3	32651	LOC_Os02g02120.1|11972.m05562|protein wall-associated kinase 3, putative, expressed
3,3	34487	LOC_Os02g01590.1|11972.m05509|protein Beta-fructofuranosidase 1 precursor, putative, expressed
2,1	37555	LOC_Os01g70860.1|11971.m13131|protein Esterase PIR7A, putative, expressed
2,3	21610	LOC_Os01g70500.1|11971.m13097|protein expressed protein
2,0	8931	LOC_Os01g69040.1|11971.m13005|protein Zinc finger, C3HC4 type family protein, expressed
2,1	1638	LOC_Os01g68770.1|11971.m12979|protein Selenium-binding protein, putative, expressed
2,6	34750	LOC_Os01g68140.1|11971.m12917|protein expressed protein

	2,2	8977	LOC_Os01g67960.1|11971.m12900|protein GPI transamidase component PIG-S, putative, expressed
	2,3	7913	LOC_Os01g62480.1|11971.m12370|protein Multicopper oxidase family protein, expressed
	4,3	25879	LOC_Os01g61610.3|11971.m43253|protein oxidoreductase, 2OG-Fe oxygenase family protein, expressed
	3,6	15332	LOC_Os01g61160.1|11971.m12243|protein laccase, putative, expressed
	2,5	6532	LOC_Os01g59100.1|11971.m42505|protein glucosyltransferase, putative, expressed
	4,5	24677	LOC_Os01g57570.1|11971.m11908|protein NADPH-dependent FMN reductase family protein, expressed
	2,2	37406	LOC_Os01g57570.1|11971.m11908|protein NADPH-dependent FMN reductase family protein, expressed
	3,5	22351	LOC_Os01g57510.1|11971.m11902|protein D-mannose binding lectin family protein, expressed
	2,7	18291	LOC_Os01g56610.1|11971.m11818|protein Homocysteine S-methyltransferase 4, putative, expressed
	2,2	535	LOC_Os01g55650.1|11971.m11722|protein patatin, putative, expressed
	2,0	20764	LOC_Os01g54010.1|11971.m11565|protein Transferrin receptor-like dimerisation domain containing protein, expressed
	2,0	13361	LOC_Os01g52630.1|11971.m11436|protein Regulator of chromosome condensation family protein, expressed
	2,5	7719	LOC_Os01g52500.4|11971.m42715|protein NADP-dependent malic enzyme, putative, expressed
	2,3	18452	LOC_Os01g51010.1|11971.m11282|protein expressed protein
	10,0	12190	LOC_Os01g50700.1|11971.m11251|protein Dehydrin family protein, expressed
	10,6	5787	LOC_Os01g49370.1|11971.m11123|protein expressed protein
	2,3	8675	LOC_Os01g47410.1|11971.m10936|protein Aspartic proteinase oryzasin-1 precursor, putative, expressed
	6,7	6118	LOC_Os01g46600.1|11971.m10856|protein seed maturation protein PM41, putative, expressed
	3,5	27713	LOC_Os01g45660.1|11971.m10767|protein hypothetical protein
	2,4	22035	LOC_Os01g45660.1|11971.m10767|protein hypothetical protein
	2,2	6389	LOC_Os01g41710.2|11971.m42672|protein Chlorophyll a-b binding protein 2, chloroplast precursor, putative, expressed
	4,0	24771	LOC_Os01g40870.1|11971.m10356|protein Aldehyde dehydrogenase, mitochondrial precursor, putative, expressed
	2,6	3275	LOC_Os01g29840.1|11971.m09413|protein no apical meristem, putative, expressed
	12,7	18249	LOC_Os01g19770.1|11971.m08514|protein stress-inducible membrane pore protein, putative, expressed
	2,5	1712	LOC_Os01g12190.1|11971.m07834|protein expressed protein
	4,1	21121	LOC_Os01g08860.1|11971.m07511|protein 17.5 kDa class II heat shock protein, putative, expressed
	2,1	33753	LOC_Os01g07360.1|11971.m07362|protein CENP-E like kinetochore protein, putative, expressed
	2,0	33544	LOC_Os01g07360.1|11971.m07362|protein CENP-E like kinetochore protein, putative, expressed
	6,3	14940	LOC_Os01g04370.1|11971.m07072|protein 16.9 kDa class I heat shock protein, putative, expressed
	5,2	5136	LOC_Os01g04370.1|11971.m07072|protein 16.9 kDa class I heat shock protein, putative, expressed
	4,9	9705	LOC_Os01g04370.1|11971.m07072|protein 16.9 kDa class I heat shock protein, putative, expressed
	4,1	20834	LOC_Os01g04370.1|11971.m07072|protein 16.9 kDa class I heat shock protein, putative, expressed
	11,8	4037	LOC_Os01g02120.1|11971.m06854|protein MOTHER of FT and TF1 protein, putative, expressed
	3,6	37805	LOC_Os01g02120.1|11971.m06854|protein MOTHER of FT and TF1 protein, putative, expressed
Unknown			
	61,2	24448	
	26,4	2443	
	26,3	29579	
	17,8	8939	
	17,1	26630	
	12,6	32399	
	12,5	36616	
	11,3	20290	
	10,3	29422	
	9,9	33421	
	8,9	5996	
	8,8	12748	
	8,4	30187	
	8,1	6388	

8,0	8069
7,9	26310
7,4	18754
7,1	13227
6,9	9803
6,7	1809
6,6	32842
6,5	34091
6,4	28560
6,0	36700
5,9	22762
5,7	19925
5,7	30425
5,6	37262
5,5	11339
5,3	4388
5,2	31239
4,9	774
4,9	1247
4,9	7768
4,8	36670
4,6	4471
4,6	830
4,4	32756
4,4	19639
4,4	2222
4,3	24703
4,2	30410
4,1	33160
4,1	363
4,0	26221
3,9	15237
3,9	35062
3,9	16444
3,8	6395
3,8	13160
3,8	582
3,7	34835
3,7	29574
3,7	36915
3,7	6176
3,6	13171
3,6	26035
3,6	36231
3,6	32983
3,6	36229
3,5	1095
3,5	19449
3,5	11433

3,5	17969
3,4	12898
3,4	15833
3,4	34816
3,4	17140
3,3	20965
3,3	20484
3,2	15226
3,2	12818
3,2	32758
3,2	25673
3,2	23876
3,2	5524
3,1	32603
3,1	29267
3,1	36739
3,1	8826
3,1	6611
3,0	29366
3,0	17883
3,0	9846
3,0	20030
3,0	23163
3,0	21035
3,0	16082
3,0	6077
3,0	14478
3,0	16437
2,9	20628
2,9	20799
2,9	24159
2,9	26186
2,9	33593
2,9	26264
2,8	11254
2,8	13399
2,8	33801
2,8	35214
2,8	26461
2,8	234
2,8	11266
2,8	36856
2,8	20436
2,8	15449
2,7	30549
2,7	11216
2,7	13531
2,7	35728
2,7	9929

2,7	20594
2,7	438
2,7	35204
2,7	15466
2,6	3235
2,6	22606
2,6	11944
2,6	20775
2,6	31497
2,6	11813
2,6	26488
2,6	33805
2,6	2138
2,6	12170
2,6	23162
2,6	23050
2,5	8026
2,5	8475
2,5	11202
2,5	9996
2,5	8869
2,5	34014
2,5	27370
2,5	12727
2,5	30413
2,4	8891
2,4	35051
2,4	27192
2,4	5690
2,4	14234
2,4	11723
2,4	21007
2,3	11586
2,3	12012
2,3	12135
2,3	7201
2,3	22083
2,3	27877
2,3	26249
2,3	24008
2,3	17165
2,3	14993
2,3	21984
2,3	7899
2,3	32728
2,3	32620
2,2	37090
2,2	16561
2,2	674

2,2	16328
2,2	34505
2,2	37766
2,2	36023
2,2	11162
2,2	10390
2,2	27042
2,2	26813
2,2	3149
2,1	20277
2,1	36013
2,1	7319
2,1	10922
2,1	25873
2,1	25781
2,1	10663
2,1	32100
2,1	4878
2,1	33727
2,1	21669
2,1	29091
2,1	9972
2,1	1473
2,1	22016
2,1	32924
2,1	32752
2,1	30926
2,1	24555
2,1	13943
2,1	19979
2,0	24342
2,0	31311
2,0	13609
2,0	17129
2,0	10422
2,0	37244
2,0	30955
2,0	5949
2,0	22959
2,0	4172

**Nobeokabouzu-komugi 3 dai specific up-regulated genes**			
**Gene classes**	**Fold Change**	**Probe ID**	**Annotation**
**JA and ET related genes**			
JA	4,8	33636	LOC_Os03g07530.3|11973.m34796|protein kelch repeat-containing F-box family protein, putative, expressed
	2,9	29393	LOC_Os03g07530.3|11973.m34796|protein kelch repeat-containing F-box family protein, putative, expressed
	2,5	14730	LOC_Os03g07530.3|11973.m34796|protein kelch repeat-containing F-box family protein, putative, expressed
Lipoxygenase	5,5	17913	LOC_Os03g49350.1|11973.m09936|protein Lipoxygenase 3, putative, expressed
	2,2	12436	LOC_Os03g49350.1|11973.m09936|protein Lipoxygenase 3, putative, expressed

Fatty acid metabolism & derivatives/Lipid metabolism	2,2	9816	LOC_Os03g16060.1|11973.m07043|protein lipase, putative, expressed
	4,3	24580	LOC_Os11g43510.1|11981.m08186|protein Lipase family protein, expressed
	6,4	27927	LOC_Os11g09010.1|11981.m05098|protein Lipase family protein, expressed
	2,6	13461	LOC_Os01g14080.1|11971.m08014|protein lipase class 3, putative, expressed
Lipid metabolism/Lipid TAG synthesis	2,4	21980	LOC_Os09g37100.1|11979.m06658|protein Phospholipase D delta, putative, expressed
	3,4	26595	LOC_Os04g54200.1|11974.m10312|protein diacylglycerol kinase, putative, expressed
	2,7	5790	LOC_Os05g49900.1|11975.m09060|protein Fatty acid elongase, putative, expressed
	3,9	17710	LOC_Os05g49290.3|11975.m27982|protein very-long-chain fatty acid condensing enzyme, putative, expressed
	45,8	31129	LOC_Os04g48850.1|11974.m09820|protein 1-aminocyclopropane-1-carboxylate synthase, putative, expressed
Ethylen biosynthesis	5,1	36839	LOC_Os06g11240.1|11976.m05841|protein 12-oxophytodienoate reductase 2, putative, expressed
	6,9	37106	LOC_Os06g11210.1|11976.m05838|protein 12-oxophytodienoate reductase 2, putative, expressed
	2,1	29522	LOC_Os02g54160.1|11972.m10445|protein ethylene response element binding protein, putative, expressed
**Cysteine-rich Antimicrobial peptides (AMPs)**			
Serine protease inhibitor	3,1	23915	LOC_Os01g03380.1|11971.m06977|protein Bowman-Birk type bran trypsin inhibitor precursor, putative
	2,6	34570	LOC_Os01g03380.1|11971.m06977|protein Bowman-Birk type bran trypsin inhibitor precursor, putative
**GDSL-lipase**			
	3,1	26455	LOC_Os06g34070.1|11976.m07943|protein GDSL-like Lipase/Acylhydrolase family protein, expressed
	9,6	13587	LOC_Os06g06290.1|11976.m05355|protein GDSL-like Lipase/Acylhydrolase family protein, expressed
	3,6	9845	LOC_Os06g06290.1|11976.m05355|protein GDSL-like Lipase/Acylhydrolase family protein, expressed
	2,4	13737	LOC_Os01g11650.1|11971.m07781|protein GDSL-like Lipase/Acylhydrolase family protein, expressed
**Proteolysis**			
	4,2	3039	LOC_Os06g24990.1|11976.m07147|protein Xylanase inhibitor protein 1 precursor, putative, expressed
	2,1	34322	LOC_Os04g26834.1|11974.m07808|protein aspartyl protease family protein, putative, expressed
	57,0	8036	LOC_Os02g48870.1|11972.m09921|protein Eukaryotic aspartyl protease family protein, expressed
	5,6	16012	LOC_Os01g56930.1|11971.m11850|protein aspartyl protease family protein, putative, expressed
	4,9	21397	LOC_Os04g37550.1|11974.m08744|protein Eukaryotic aspartyl protease family protein, expressed
	2,9	37583	LOC_Os11g08100.1|11981.m05006|protein Eukaryotic aspartyl protease family protein, expressed
	3,4	31487	LOC_Os08g44640.1|11978.m08436|protein Serine carboxypeptidase family protein, expressed
	2,6	30916	LOC_Os08g44640.1|11978.m08436|protein Serine carboxypeptidase family protein, expressed
	2,5	17725	LOC_Os02g46260.4|11972.m34030|protein Serine carboxypeptidase family protein, expressed
	4,8	5250	LOC_Os01g61690.1|11971.m12294|protein Serine carboxypeptidase II precursor, putative, expressed
	2,8	31017	LOC_Os01g56150.1|11971.m11771|protein Serine carboxypeptidase S28 family protein, expressed
	3,6	17512	LOC_Os04g57440.1|11974.m10629|protein Cysteine protease 1 precursor, putative, expressed
	2,1	22571	LOC_Os04g57440.1|11974.m10629|protein Cysteine protease 1 precursor, putative, expressed
	2,4	16384	LOC_Os05g51630.2|11975.m27653|protein early-responsive to dehydration protein, putative, expressed
	4,7	30712	LOC_Os04g35140.1|11974.m08604|protein Subtilisin N-terminal Region family protein, expressed
	4,5	3368	LOC_Os01g60410.3|11971.m42744|protein Ubiquitin-conjugating enzyme E2-17 kDa, putative, expressed
	2,4	622	LOC_Os01g60410.3|11971.m42744|protein Ubiquitin-conjugating enzyme E2-17 kDa, putative, expressed
	98,3	5056	LOC_Os09g27930.1|11979.m05944|protein UBiQuitin family member, putative, expressed
	3,2	21199	LOC_Os09g27930.1|11979.m05944|protein UBiQuitin family member, putative, expressed
	2,0	37285	LOC_Os09g27930.1|11979.m05944|protein UBiQuitin family member, putative, expressed
	2,2	15245	LOC_Os08g08760.1|11978.m05011|protein Ubiquitin family protein
	2,4	14977	LOC_Os06g46770.1|11976.m09203|protein polyubiquitin containing 7 ubiquitin monomers, putative, expressed
	2,3	7213	LOC_Os06g46770.1|11976.m09203|protein polyubiquitin containing 7 ubiquitin monomers, putative, expressed
	2,2	4496	LOC_Os06g46770.1|11976.m09203|protein polyubiquitin containing 7 ubiquitin monomers, putative, expressed
	2,1	13611	LOC_Os06g46770.1|11976.m09203|protein polyubiquitin containing 7 ubiquitin monomers, putative, expressed
**Peroxidases**			
	94,7	19329	LOC_Os07g48050.1|11977.m09020|protein Peroxidase 2 precursor, putative, expressed
	9,6	14508	LOC_Os07g47990.1|11977.m09014|protein Peroxidase 2 precursor, putative, expressed

	5,7	32745	LOC_Os01g22370.2|11971.m42991|protein Peroxidase family protein, expressed
	2,8	18103	LOC_Os03g17690.1|11973.m07194|protein L-ascorbate peroxidase 1, cytosolic, putative, expressed
	2,6	14428	LOC_Os03g17690.1|11973.m07194|protein L-ascorbate peroxidase 1, cytosolic, putative, expressed
	4,2	32558	LOC_Os01g19020.1|11971.m08439|protein Peroxidase family protein, expressed
**Genes related to cell wall defense**			
Plant cellulose synthesis	2,1	12417	LOC_Os02g49332.3|11972.m34039|protein cellulose synthase-like protein CslE, putative, expressed
Inactivation of fungal polygalacturonase	5,3	12447	LOC_Os11g47350.1|11981.m08515|protein Glycosyl hydrolase family 3 C terminal domain containing protein
	6,3	34987	LOC_Os09g33680.2|11979.m22014|protein Glycosyl hydrolase family 1 protein, expressed
	3,3	9014	LOC_Os05g30350.2|11975.m27797|protein Glycosyl hydrolase family 1 protein, expressed
**Secondary metabolism & detoxification**			
UDP-glycosyltransferase family	2,7	25305	LOC_Os08g31200.1|11978.m07118|protein UDP-glucoronosyl and UDP-glucosyl transferase family protein
UDP-glycosyltransferase family	3,8	8460	LOC_Os06g23560.1|11976.m07008|protein UDP-glucoronosyl and UDP-glucosyl transferase family protein, expressed
UDP-glycosyltransferase family	6,5	23698	LOC_Os05g51670.2|11975.m27994|protein UDP-glucose 4-epimerase GEPI48, putative, expressed
UDP-glycosyltransferase family	7,4	2354	LOC_Os05g45110.1|11975.m08636|protein UDP-glucoronosyl and UDP-glucosyl transferase family protein
UDP-glycosyltransferase family	4,1	550	LOC_Os05g29990.1|11975.m07235|protein UDP-D-glucuronate decarboxylase, putative, expressed
UDP-glycosyltransferase family	5,8	27074	LOC_Os04g52730.1|11974.m10166|protein UDP-glucose 4-epimerase family protein, expressed
UDP-glycosyltransferase family	5,5	17956	LOC_Os04g52730.1|11974.m10166|protein UDP-glucose 4-epimerase family protein, expressed
UDP-glycosyltransferase family	68,3	19091	LOC_Os04g44250.1|11974.m09384|protein UDP-glucoronosyl and UDP-glucosyl transferase family protein, expressed
UDP-glycosyltransferase family	3,2	35963	LOC_Os03g02670.2|11973.m34768|protein UDP-galactose transporter, putative, expressed
UDP-glycosyltransferase family	7,9	2165	LOC_Os01g53350.1|11971.m11503|protein UDP-glucoronosyl and UDP-glucosyl transferase family protein, expressed
UDP-glycosyltransferase family	3,6	4225	LOC_Os03g37010.1|11973.m08814|protein glycosyltransferase, putative, expressed
UDP-glycosyltransferase family	3,1	14884	LOC_Os03g37010.1|11973.m08814|protein glycosyltransferase, putative, expressed
UDP-glycosyltransferase family	5,4	1326	LOC_Os02g41520.1|11972.m09188|protein Glycosyl transferase family 8 protein, expressed
UDP-glycosyltransferase family	2,6	31469	LOC_Os02g41520.1|11972.m09188|protein Glycosyl transferase family 8 protein, expressed
UDP-glycosyltransferase family	2,3	1422	LOC_Os02g41520.1|11972.m09188|protein Glycosyl transferase family 8 protein, expressed
UDP-glycosyltransferase family	2,1	36196	LOC_Os02g03850.2|11972.m05734|protein Glycosyl transferase family, a/b domain containing protein, expressed
UDP-glycosyltransferase family	6,9	24392	LOC_Os01g02920.1|11971.m06931|protein glycosyltransferase, putative, expressed
Glutathione S-transferase	3,4	195	LOC_Os12g10730.2|11982.m05056|protein Glutathione S-transferase, putative, expressed
Glutathione S-transferase	2,1	35431	LOC_Os12g10730.2|11982.m05056|protein Glutathione S-transferase, putative, expressed
Glutathione S-transferase	6,2	8674	LOC_Os12g02960.1|11982.m04294|protein Glutathione S-transferase, C-terminal domain containing protein, expressed
Glutathione S-transferase	2,5	27708	LOC_Os11g37730.1|11981.m07618|protein Glutathione S-transferase, N-terminal domain containing protein, expressed
Glutathione S-transferase	3,2	9219	LOC_Os09g29200.1|11979.m06071|protein Glutathione S-transferase, N-terminal domain containing protein, expressed
Glutathione S-transferase	3,7	11686	LOC_Os07g28480.1|11977.m07120|protein glutathione S-transferase GSTU1, putative, expressed
Glutathione S-transferase	2,7	3141	LOC_Os03g57200.1|11973.m10639|protein glutathione S-transferase GSTU1, putative, expressed
Glutathione S-transferase	2,4	4156	LOC_Os03g57200.1|11973.m10639|protein glutathione S-transferase GSTU1, putative, expressed
Glutathione S-transferase	3,9	19603	LOC_Os02g35590.1|11972.m08647|protein Glutathione S-transferase zeta-class 1, putative, expressed
Glutathione S-transferase	19,8	15597	LOC_Os02g21460.1|11972.m07386|protein glutathione S-transferase, putative, expressed
Glutathione S-transferase	6,9	24907	LOC_Os01g72140.1|11971.m13250|protein Glutathione S-transferase, N-terminal domain containing protein, expressed
Glutathione S-transferase	3,5	6100	LOC_Os01g55830.2|11971.m42727|protein glutathione S-transferase GSTF2, putative, expressed
Glutathione S-transferase	13,8	4024	LOC_Os01g27260.1|11971.m09177|protein glutathione S-transferase GSTF1, putative, expressed
Glutathione S-transferase	9,2	7492	LOC_Os01g25386.1|11971.m09002|protein glutathione-conjugate transporter, putative, expressed
Glutathione S-transferase	2,6	9517	LOC_Os05g35274.5|11975.m64242|protein Galactosyltransferase family protein, expressed
	3,5	14139	LOC_Os04g44870.1|11974.m09443|protein Elicitor-responsive protein 3, putative, expressed
	18,1	25957	LOC_Os04g44240.1|11974.m09383|protein Cytokinin-O-glucosyltransferase 3, putative, expressed
	3,3	3562	LOC_Os03g50860.1|11973.m10071|protein cytokinin receptor CRE1b, putative, expressed
	4,8	14759	LOC_Os02g36840.1|11972.m08771|protein Cytokinin-O-glucosyltransferase 2, putative, expressed
	7,0	6320	LOC_Os05g35200.1|11975.m07750|protein secondary cell wall-related glycosyltransferase family 8, putative, expressed
	3,9	34402	LOC_Os01g65780.3|11971.m42775|protein secondary cell wall-related glycosyltransferase family 8, putative, expressed
	3,1	26682	LOC_Os01g65780.3|11971.m42775|protein secondary cell wall-related glycosyltransferase family 8, putative, expressed

Volatile phenylpropanoids	2,9	37480	LOC_Os02g41650.2|11972.m33782|protein Phenylalanine ammonia-lyase, putative, expressed
Volatile phenylpropanoids	2,1	16168	LOC_Os02g41650.2|11972.m33782|protein Phenylalanine ammonia-lyase, putative, expressed
Volatile phenylpropanoids	2,1	15524	LOC_Os02g41630.3|11972.m33418|protein Phenylalanine ammonia-lyase, putative, expressed
Flavonoids	2,7	33291	LOC_Os02g52840.1|11972.m10314|protein Flavonol synthase/flavanone 3-hydroxylase, putative, expressed
Cytochrome P450s	30,5	16347	LOC_Os12g32850.1|11982.m07084|protein Cytochrome P450 71E1, putative
	17,0	1186	LOC_Os12g05440.1|11982.m04537|protein Cytochrome P450 family protein, expressed
	8,3	32344	LOC_Os09g36080.1|11979.m06558|protein Cytochrome P450 family protein, expressed
	3,2	18104	LOC_Os08g37432.2|11978.m26847|protein MATE efflux family protein, expressed
	2,1	32372	LOC_Os08g03682.1|11978.m04512|protein Cytochrome P450 family protein, expressed
	3,2	10052	LOC_Os07g12830.2|11977.m29108|protein Cytochrome b5-like Heme/Steroid binding domain containing protein, expressed
	6,7	16870	LOC_Os05g41440.2|11975.m27896|protein Cytochrome P450 98A1, putative, expressed
	2,7	11964	LOC_Os05g34770.1|11975.m07707|protein Cytochrome c, putative, expressed
	2,9	27672	LOC_Os04g55960.1|11974.m10487|protein NADPH-cytochrome P450 reductase, putative, expressed
	3,3	34489	LOC_Os04g48460.2|11974.m35189|protein Cytochrome P450 family protein, expressed
	2,4	14062	LOC_Os04g48460.1|11974.m09782|protein Cytochrome P450 family protein, expressed
	2,7	21674	LOC_Os03g12500.1|11973.m06700|protein Cytochrome P450 74A2, putative, expressed
ABC transporter	4,8	28601	LOC_Os05g02890.1|11975.m04824|protein ABC transporter family protein, putative, expressed
	3,0	4900	LOC_Os05g02890.1|11975.m04824|protein ABC transporter family protein, putative, expressed
	6,3	13164	LOC_Os03g32630.1|11973.m08505|protein ABC transporter family protein, expressed
	4,9	21173	LOC_Os03g32630.1|11973.m08505|protein ABC transporter family protein, expressed
	2,4	36034	LOC_Os02g56550.1|11972.m10677|protein ABC transporter family protein, expressed
MDR	5,5	11222	LOC_Os05g10730.1|11975.m05543|protein multidrug resistance associated protein 1, putative, expressed
**Miscellaneous defense related genes**			
	2,9	31026	LOC_Os05g43840.1|11975.m08510|protein CBL-interacting serine/threonine-protein kinase 15, putative, expressed
	3,1	26878	LOC_Os05g04550.1|11975.m04988|protein CBL-interacting serine/threonine-protein kinase 1, putative, expressed
	2,8	34939	LOC_Os05g04550.1|11975.m04988|protein CBL-interacting serine/threonine-protein kinase 1, putative, expressed
	8,7	26226	LOC_Os02g02600.1|11972.m05609|protein serine/threonine-protein kinase Cx32, chloroplast precursor, putative, expressed
	5,2	12289	LOC_Os02g02600.1|11972.m05609|protein serine/threonine-protein kinase Cx32, chloroplast precursor, putative, expressed
	2,1	16414	LOC_Os03g41460.1|11973.m09212|protein Serine/threonine-protein kinase SAPK9, putative, expressed
	4,0	804	LOC_Os03g21620.1|11973.m07566|protein Serine/threonine-protein kinase AtPK19, putative, expressed
	2,8	37771	LOC_Os06g39760.1|11976.m08513|protein Serine-threonine kinase receptor-associated protein, putative, expressed
	2,6	13559	LOC_Os05g06500.1|11975.m05177|protein universal stress protein family protein, expressed
	3,9	24292	LOC_Os02g47650.1|11972.m09799|protein pathogen induced protein 2-4, putative, expressed
	8,9	10647	LOC_Os03g03700.1|11973.m05903|protein MLO protein homolog 1, putative, expressed
**Transcription & signalling**			
	2,0	19814	LOC_Os12g43550.2|11982.m08128|protein Ras-related protein RHN1, putative, expressed
	21,5	29299	LOC_Os12g41530.1|11982.m07934|protein Protein kinase domain containing protein
	17,8	22220	LOC_Os11g17380.1|11981.m05821|protein Protein kinase domain containing protein
	10,9	33107	LOC_Os10g09620.1|11980.m04304|protein Protein kinase domain containing protein, expressed
	4,7	14184	LOC_Os10g09620.1|11980.m04304|protein Protein kinase domain containing protein, expressed
	3,0	26124	LOC_Os09g37230.1|11979.m06670|protein protein kinase family protein, putative, expressed
	4,8	34502	LOC_Os09g03620.1|11979.m03732|protein Protein kinase domain containing protein, expressed
	2,3	22384	LOC_Os09g03620.1|11979.m03732|protein Protein kinase domain containing protein, expressed
	8,5	10651	LOC_Os08g42580.4|11978.m62032|protein Protein kinase domain containing protein, expressed
	5,2	22243	LOC_Os08g02050.1|11978.m26423|protein protein kinase family protein, putative, expressed
	4,5	28029	LOC_Os07g40550.3|11977.m29001|protein protein kinase family protein, putative, expressed
	5,2	10212	LOC_Os07g35660.1|11977.m07822|protein Protein kinase domain containing protein, expressed
	2,7	27321	LOC_Os06g49430.2|11976.m31928|protein Protein kinase domain containing protein, expressed

6,2	31843	LOC_Os06g49430.1|11976.m09467|protein Protein kinase domain containing protein, expressed
3,0	35808	LOC_Os06g45350.4|11976.m32137|protein protein kinase, putative, expressed
18,0	6164	LOC_Os06g18000.1|11976.m06512|protein Protein kinase domain containing protein, expressed
14,8	16497	LOC_Os06g18000.1|11976.m06512|protein Protein kinase domain containing protein, expressed
2,7	25100	LOC_Os06g08280.1|11976.m05549|protein Protein kinase domain containing protein, expressed
3,3	5464	LOC_Os06g07070.1|11976.m05431|protein Protein kinase domain containing protein, expressed
3,6	29048	LOC_Os06g05520.1|11976.m05280|protein Protein kinase domain containing protein, expressed
2,6	25122	LOC_Os05g04520.2|11975.m27688|protein Protein kinase domain containing protein, expressed
2,5	33327	LOC_Os05g04520.2|11975.m27688|protein Protein kinase domain containing protein, expressed
4,4	4501	LOC_Os04g58750.3|11974.m35528|protein Protein kinase APK1B, chloroplast precursor, putative, expressed
8,1	37629	LOC_Os04g58250.1|11974.m10707|protein protein kinase family protein, putative, expressed
2,9	719	LOC_Os04g58250.1|11974.m10707|protein protein kinase family protein, putative, expressed
5,6	30274	LOC_Os04g30030.1|11974.m08109|protein Protein kinase domain containing protein
5,7	23786	LOC_Os04g01874.1|11974.m05503|protein Protein kinase domain containing protein, expressed
5,1	1536	LOC_Os03g43760.1|11973.m09416|protein protein kinase, putative, expressed
2,6	27980	LOC_Os03g17300.1|11973.m07159|protein protein kinase, putative, expressed
2,2	19416	LOC_Os03g13820.1|11973.m06826|protein protein kinase family protein, putative, expressed
3,9	22543	LOC_Os02g57420.1|11972.m10764|protein Protein kinase domain containing protein, expressed
3,4	37814	LOC_Os02g07790.1|11972.m06125|protein Protein kinase domain containing protein, expressed
2,9	15939	LOC_Os01g60280.1|11971.m12158|protein Protein kinase domain containing protein, expressed
3,8	3418	LOC_Os01g54480.2|11971.m43192|protein Protein kinase domain containing protein, expressed
2,2	18704	LOC_Os12g40570.2|11982.m07841|protein WRKY DNA binding domain containing protein, expressed
5,3	14098	LOC_Os11g02480.1|11981.m04447|protein WRKY DNA binding domain containing protein, expressed
3,2	37012	LOC_Os09g16510.1|11979.m04911|protein WRKY DNA binding domain containing protein, expressed
2,5	32571	LOC_Os08g38990.3|11978.m26522|protein WRKY DNA binding domain containing protein, expressed
6,5	10743	LOC_Os06g44010.1|11976.m08929|protein WRKY2 protein, putative, expressed
7,2	27696	LOC_Os04g51560.1|11974.m10052|protein WRKY DNA binding domain containing protein, expressed
4,1	14023	LOC_Os11g38800.2|11981.m28902|protein Zinc finger protein, putative, expressed
3,5	11143	LOC_Os11g38800.2|11981.m28902|protein Zinc finger protein, putative, expressed
3,0	8307	LOC_Os09g26400.1|11979.m05792|protein Zinc finger, C3HC4 type family protein, expressed
2,4	18340	LOC_Os09g26400.1|11979.m05792|protein Zinc finger, C3HC4 type family protein, expressed
5,0	24653	LOC_Os09g25430.1|11979.m05695|protein Zinc finger, C2H2 type family protein, expressed
4,7	33234	LOC_Os09g25420.1|11979.m05694|protein Zinc finger, C2H2 type family protein, expressed
3,2	2789	LOC_Os08g43480.1|11978.m08322|protein Zinc finger, C3HC4 type family protein, expressed
2,1	32962	LOC_Os08g02850.1|11978.m04428|protein zinc finger family protein, putative, expressed
3,3	19638	LOC_Os07g38580.1|11977.m08104|protein zinc finger family protein, putative, expressed
3,1	7662	LOC_Os07g38240.1|11977.m08071|protein Zinc finger protein, putative, expressed
10,8	4855	LOC_Os07g38090.2|11977.m28994|protein Zinc finger C-x8-C-x5-C-x3-H type family protein, expressed
2,7	5376	LOC_Os07g38090.2|11977.m28994|protein Zinc finger C-x8-C-x5-C-x3-H type family protein, expressed
4,9	25966	LOC_Os06g48040.1|11976.m09330|protein Zinc finger, C3HC4 type family protein, expressed
8,2	19646	LOC_Os06g47840.1|11976.m09310|protein Zinc finger, C2H2 type family protein, expressed
6,7	17045	LOC_Os06g47840.1|11976.m09310|protein Zinc finger, C2H2 type family protein, expressed
5,6	128	LOC_Os06g47840.1|11976.m09310|protein Zinc finger, C2H2 type family protein, expressed
2,6	17630	LOC_Os06g47840.1|11976.m09310|protein Zinc finger, C2H2 type family protein, expressed
2,0	2187	LOC_Os06g46366.1|11976.m31777|protein Zinc finger, C3HC4 type family protein, expressed
7,5	35307	LOC_Os06g03580.1|11976.m05090|protein Zinc finger, C3HC4 type family protein, expressed
2,1	33402	LOC_Os05g33000.2|11975.m27813|protein zinc finger family protein, putative, expressed
4,3	17090	LOC_Os05g08610.3|11975.m27724|protein Zinc finger, C3HC4 type family protein, expressed
4,6	3236	LOC_Os05g03760.1|11975.m04910|protein Zinc finger C-x8-C-x5-C-x3-H type family protein, expressed

	2,9	1133	LOC_Os04g51400.2|11974.m35195|protein Zinc finger, C3HC4 type family protein, expressed
	3,6	30596	LOC_Os04g48050.1|11974.m09745|protein zinc finger family protein, putative, expressed
	4,1	4774	LOC_Os03g57500.1|11973.m10668|protein zinc finger family protein, putative, expressed
	3,7	23596	LOC_Os03g57500.1|11973.m10668|protein zinc finger family protein, putative, expressed
	6,5	27544	LOC_Os03g04890.1|11973.m06015|protein zinc finger family protein, putative, expressed
	3,7	7149	LOC_Os03g04890.1|11973.m06015|protein zinc finger family protein, putative, expressed
	2,1	33231	LOC_Os03g02160.2|11973.m78833|protein Zinc finger C-x8-C-x5-C-x3-H type family protein, expressed
	2,2	6953	LOC_Os02g45240.1|11972.m09563|protein zinc finger family protein, putative, expressed
	4,8	1580	LOC_Os01g72480.1|11971.m13283|protein Zinc finger, C3HC4 type family protein, expressed
	2,0	65	LOC_Os01g69040.1|11971.m13005|protein Zinc finger, C3HC4 type family protein, expressed
	7,8	31921	LOC_Os01g67850.1|11971.m12889|protein Zinc finger, C3HC4 type family protein, expressed
	3,0	1399	LOC_Os01g67850.1|11971.m12889|protein Zinc finger, C3HC4 type family protein, expressed
	2,1	31460	LOC_Os01g67850.1|11971.m12889|protein Zinc finger, C3HC4 type family protein, expressed
	2,0	5092	LOC_Os01g67850.1|11971.m12889|protein Zinc finger, C3HC4 type family protein, expressed
	2,1	10270	LOC_Os01g65080.2|11971.m42769|protein zinc finger family protein, putative, expressed
	2,5	37347	LOC_Os01g19800.1|11971.m08517|protein Zinc finger, C3HC4 type family protein, expressed
	3,2	32532	LOC_Os01g08340.1|11971.m07459|protein zinc finger family protein, putative, expressed
	4,7	32146	LOC_Os01g05500.2|11971.m42863|protein zinc finger in N-recognin family protein, expressed
	2,5	2623	LOC_Os06g45540.1|11976.m09082|protein zinc-binding protein, putative, expressed
	3,3	36518	LOC_Os06g41770.1|11976.m08709|protein bZIP transcription factor family protein, expressed
	2,2	2403	LOC_Os01g05500.2|11971.m42863|protein zinc finger in N-recognin family protein, expressed
	2,5	34984	LOC_Os10g38950.1|11980.m06663|protein Mitogen-activated protein kinase homolog 4, putative, expressed
	4,4	15568	LOC_Os06g48590.2|11976.m31922|protein Mitogen-activated protein kinase homolog NTF3, putative, expressed
	3,7	17466	LOC_Os06g48590.2|11976.m31922|protein Mitogen-activated protein kinase homolog NTF3, putative, expressed
LRR-proteins	11,1	34295	LOC_Os10g33140.1|11980.m06146|protein Leucine Rich Repeat family protein, expressed
LRR-proteins	7,7	1469	LOC_Os07g07990.1|11977.m05258|protein Leucine Rich Repeat family protein, expressed
LRR-proteins	2,8	17236	LOC_Os06g07130.1|11976.m05437|protein Leucine Rich Repeat family protein, expressed
LRR-proteins	12,6	29310	LOC_Os05g01444.1|11975.m28002|protein Leucine Rich Repeat family protein, expressed
LRR-proteins	7,2	32921	LOC_Os04g41030.1|11974.m09083|protein Leucine Rich Repeat family protein, expressed
LRR-proteins	3,4	26605	LOC_Os04g41030.1|11974.m09083|protein Leucine Rich Repeat family protein, expressed
LRR-proteins	4,7	32468	LOC_Os04g28210.1|11974.m07938|protein Leucine Rich Repeat family protein, expressed
LRR-proteins	2,8	37548	LOC_Os04g15660.1|11974.m06752|protein Leucine Rich Repeat family protein, expressed
LRR-proteins	2,9	11489	LOC_Os03g04270.1|11973.m05958|protein Leucine Rich Repeat family protein, expressed
LRR-proteins	2,4	24385	LOC_Os03g04270.1|11973.m05958|protein Leucine Rich Repeat family protein, expressed
LRR-proteins	2,9	8564	LOC_Os02g34790.1|11972.m08568|protein Leucine Rich Repeat family protein
LRR-proteins	3,1	22402	LOC_Os01g72990.1|11971.m13330|protein leucine-rich repeat transmembrane protein kinase, putative, expressed
LRR-proteins	2,6	25914	LOC_Os01g72990.1|11971.m13330|protein leucine-rich repeat transmembrane protein kinase, putative, expressed
LRR-proteins	2,8	12326	LOC_Os01g48390.1|11971.m11030|protein leucine-rich repeat transmembrane protein kinase, putative, expressed
	5,4	15122	LOC_Os01g67480.1|11971.m12852|protein Helix-loop-helix DNA-binding domain containing protein, expressed
	3,3	7903	LOC_Os07g43530.1|11977.m08581|protein Helix-loop-helix DNA-binding domain containing protein, expressed
	7,6	34053	LOC_Os09g31000.1|11979.m06218|protein EF hand family protein, expressed
	3,6	6368	LOC_Os09g31000.1|11979.m06218|protein EF hand family protein, expressed
	2,9	33657	LOC_Os02g55880.1|11972.m10612|protein EF hand family protein, expressed
	3,7	31887	LOC_Os09g01960.1|11979.m03570|protein Myb-like DNA-binding domain containing protein, expressed
	2,9	18988	LOC_Os06g45840.1|11976.m09112|protein myb family transcription factor, putative, expressed
	2,8	26268	LOC_Os04g50770.1|11974.m09974|protein Myb-related protein Zm1, putative, expressed
	2,6	1660	LOC_Os04g49450.2|11974.m35436|protein myb-like DNA-binding domain, SHAQKYF class family protein, expressed
	5,7	2230	LOC_Os04g43680.1|11974.m09327|protein Myb-related protein Myb4, putative, expressed
	2,4	9884	LOC_Os01g09280.1|11971.m07553|protein myb-like DNA-binding domain, SHAQKYF class family protein, expressed

	2,1	33478	LOC_Os10g04110.1|11980.m03828|protein NB-ARC domain containing protein, expressed
	6,1	20925	LOC_Os06g46270.1|11976.m09153|protein NAC-domain containing protein 21/22, putative, expressed
	2,6	25065	LOC_Os03g21060.1|11973.m07513|protein NAC domain transcription factor, putative, expressed
	2,2	12131	LOC_Os08g14850.1|11978.m05608|protein NB-ARC domain containing protein, expressed
	7,9	7190	LOC_Os08g10430.1|11978.m05176|protein NB-ARC domain containing protein, expressed
	6,3	16216	LOC_Os02g07430.1|11972.m06090|protein MADS-box protein ZMM17, putative, expressed
	3,1	403	LOC_Os01g66820.1|11971.m12788|protein receptor-like protein kinase, putative
**Hormone metabolism**			
Auxine	2,6	15987	LOC_Os09g29940.2|11979.m06145|protein Auxin-independent growth promoter, putative, expressed
	2,3	12309	LOC_Os06g46410.1|11976.m09167|protein auxin response factor 3, putative, expressed
	4,8	20595	LOC_Os01g60230.3|11971.m97552|protein auxin efflux carrier family protein, putative, expressed
	2,4	26140	LOC_Os01g60230.3|11971.m97552|protein auxin efflux carrier family protein, putative, expressed
	2,6	3176	LOC_Os01g08320.1|11971.m07457|protein Auxin-induced protein 22B, putative, expressed
Gibberellin	11,4	16072	LOC_Os07g39470.1|11977.m08189|protein Chitin-inducible gibberellin-responsive protein 2, putative, expressed
	11,3	5930	LOC_Os07g39470.1|11977.m08189|protein Chitin-inducible gibberellin-responsive protein 2, putative, expressed
	6,2	16631	LOC_Os07g39470.1|11977.m08189|protein Chitin-inducible gibberellin-responsive protein 2, putative, expressed
	4,0	7128	LOC_Os07g36170.2|11977.m29215|protein Chitin-inducible gibberellin-responsive protein 1, putative, expressed
**Un categorized**			
	2,8	32240	LOC_Os12g44380.2|11982.m27001|protein sucrose/H+ symporter family protein, expressed
	3,4	12070	LOC_Os12g43490.1|11982.m08122|protein Alpha-amylase/trypsin inhibitor, putative, expressed
	3,6	26191	LOC_Os12g42980.2|11982.m26986|protein Cysteine synthase, putative, expressed
	2,1	15127	LOC_Os12g42420.1|11982.m08021|protein expressed protein
	3,1	1164	LOC_Os12g40830.2|11982.m26961|protein Pyridoxal kinase, putative, expressed
	4,2	23353	LOC_Os12g39120.1|11982.m07697|protein protein phosphatase 2C family protein, putative, expressed
	10,4	14718	LOC_Os12g38490.1|11982.m07635|protein GRAS family transcription factor containing protein, expressed
	2,3	33410	LOC_Os12g38140.1|11982.m07600|protein expressed protein
	2,7	2427	LOC_Os12g35480.1|11982.m07342|protein expressed protein
	2,8	24355	LOC_Os12g34850.1|11982.m07278|protein Fibronectin type III domain containing protein, expressed
	2,4	18139	LOC_Os12g33100.1|11982.m07108|protein guanylate kinase, putative, expressed
	4,6	10479	LOC_Os12g31880.1|11982.m06996|protein Eukaryotic translation initiation factor 5B, putative, expressed
	2,1	1933	LOC_Os12g31880.1|11982.m06996|protein Eukaryotic translation initiation factor 5B, putative, expressed
	21,3	22549	LOC_Os12g26290.5|11982.m27040|protein alpha-dioxygenase, putative, expressed
	11,4	20832	LOC_Os12g25090.2|11982.m56494|protein expressed protein
	8,5	13186	LOC_Os12g24320.1|11982.m06266|protein Cell Division Protein AAA ATPase family, putative, expressed
	3,9	11500	LOC_Os12g24184.1|11982.m06252|protein Beta-galactosidase precursor, putative, expressed
	2,2	30334	LOC_Os12g13320.1|11982.m05305|protein Argininosuccinate synthase, chloroplast precursor, putative, expressed
	3,2	17954	LOC_Os12g08280.1|11982.m04815|protein expressed protein
	3,2	1811	LOC_Os12g08280.1|11982.m04815|protein expressed protein
	2,6	36846	LOC_Os12g08260.4|11982.m26854|protein 2-oxoisovalerate dehydrogenase alpha subunit, mitochondrial precursor
	2,0	8158	LOC_Os12g08210.1|11982.m04808|protein expressed protein
	4,8	30357	LOC_Os12g05420.1|11982.m04535|protein calmodulin-binding family protein, putative, expressed
	3,8	18894	LOC_Os12g05420.1|11982.m04535|protein calmodulin-binding family protein, putative, expressed
	4,9	11972	LOC_Os12g04440.1|11982.m04438|protein 2-isopropylmalate synthase B, putative, expressed
	5,4	20148	LOC_Os12g04220.1|11982.m04416|protein Calcium-transporting ATPase 4, plasma membrane-type, putative, expressed
	2,1	17092	LOC_Os12g03860.4|11982.m27021|protein major facilitator superfamily antiporter, putative, expressed
	5,7	18586	LOC_Os12g03720.1|11982.m04369|protein SIS domain containing protein, expressed
	2,4	34806	LOC_Os12g03650.1|11982.m04362|protein D-mannose binding lectin family protein
	2,6	5184	LOC_Os12g02260.1|11982.m04227|protein nucleotidyltransferase family protein, putative, expressed
	2,2	30990	LOC_Os12g02170.1|11982.m04219|protein hypothetical protein

2,1	14037	LOC_Os12g01530.1|11982.m04157|protein Ferritin 1, chloroplast precursor, putative, expressed
2,4	16803	LOC_Os12g01030.1|11982.m04108|protein hydrolase, alpha/beta fold family protein
3,7	24188	LOC_Os11g47890.1|11981.m08586|protein GRAS family transcription factor containing protein, expressed
2,2	22139	LOC_Os11g47710.1|11981.m08571|protein Charged multivesicular body protein 2a, putative, expressed
3,7	4061	LOC_Os11g44870.2|11981.m28869|protein expressed protein
2,4	6280	LOC_Os11g37690.1|11981.m07614|protein RabGAP/TBC domain-containing protein, putative, expressed
3,4	7097	LOC_Os11g37640.1|11981.m07609|protein ADP-ribosylation factor-like protein 5, putative, expressed
2,6	3746	LOC_Os11g37060.1|11981.m07554|protein F-box domain containing protein, expressed
2,4	34850	LOC_Os11g34350.1|11981.m07293|protein ATP-binding cassette sub-family E member 1, putative, expressed
12,5	6641	LOC_Os11g33300.1|11981.m07229|protein O-methyltransferase family protein
5,3	1533	LOC_Os11g33240.1|11981.m07223|protein Citrate synthase 4, mitochondrial precursor, putative, expressed
2,4	17521	LOC_Os11g17080.3|11981.m28779|protein Extracellular signal-regulated kinase 1, putative, expressed
4,1	15463	LOC_Os11g14190.2|11981.m28665|protein expressed protein
4,2	37534	LOC_Os11g10980.1|11981.m05291|protein pyruvate kinase family protein, expressed
2,4	11572	LOC_Os11g10980.1|11981.m05291|protein pyruvate kinase family protein, expressed
2,7	37778	LOC_Os11g10480.4|11981.m28756|protein Alcohol dehydrogenase 1, putative, expressed
10,4	13170	LOC_Os11g10480.1|11981.m05243|protein Alcohol dehydrogenase 1, putative, expressed
2,8	932	LOC_Os11g10480.1|11981.m05243|protein Alcohol dehydrogenase 1, putative, expressed
5,2	10347	LOC_Os11g08300.2|11981.m28749|protein aldehyde dehydrogenase family protein, expressed
3,8	24225	LOC_Os11g08300.2|11981.m28749|protein aldehyde dehydrogenase family protein, expressed
3,6	17265	LOC_Os11g08300.2|11981.m28749|protein aldehyde dehydrogenase family protein, expressed
3,3	18027	LOC_Os11g08300.2|11981.m28749|protein aldehyde dehydrogenase family protein, expressed
3,4	32986	LOC_Os11g08300.1|11981.m05026|protein aldehyde dehydrogenase family protein, expressed
3,0	15475	LOC_Os11g07916.1|11981.m61743|protein nitrogen fixation protein, putative, expressed
4,7	24914	LOC_Os11g07440.2|11981.m28745|protein Neutral/alkaline invertase, putative, expressed
3,0	1085	LOC_Os11g06440.1|11981.m04841|protein expressed protein
3,9	24196	LOC_Os11g05880.1|11981.m04784|protein exocyst subunit EXO70 family protein, putative, expressed
2,8	30807	LOC_Os11g04460.1|11981.m04644|protein Calcium-transporting ATPase 4, plasma membrane-type
2,0	15858	LOC_Os11g02230.1|11981.m04422|protein hypothetical protein
6,7	37292	LOC_Os11g02200.1|11981.m04419|protein expressed protein
3,6	33490	LOC_Os11g02200.1|11981.m04419|protein expressed protein
2,4	28796	LOC_Os11g01280.1|11981.m04333|protein expressed protein
3,4	1894	LOC_Os10g42710.6|11980.m21812|protein Poly polymerase catalytic domain containing protein, expressed
2,4	9243	LOC_Os10g42710.1|11980.m07023|protein Poly polymerase catalytic domain containing protein, expressed
5,7	18301	LOC_Os10g42670.1|11980.m07020|protein xyloglucan endotransglucosylase/hydrolase protein 28precursor
2,2	14712	LOC_Os10g42620.1|11980.m07015|protein NAD dependent epimerase/dehydratase family protein, expressed
2,4	22941	LOC_Os10g42320.1|11980.m06988|protein NMD3 family protein, expressed
3,7	13443	LOC_Os10g41970.1|11980.m06953|protein Methyltransferase, putative, expressed
2,0	9408	LOC_Os10g41970.1|11980.m06953|protein Methyltransferase, putative, expressed
7,3	14555	LOC_Os10g41020.1|11980.m06867|protein oxidoreductase, 2OG-Fe oxygenase family protein, expressed
6,5	21474	LOC_Os10g40780.2|11980.m06845|protein transcriptional regulator, putative, expressed
2,7	12550	LOC_Os10g38060.2|11980.m21798|protein Phospholipase D beta 1, putative, expressed
4,8	4102	LOC_Os10g37830.1|11980.m50429|protein F-box family protein, putative, expressed
2,9	30942	LOC_Os10g37340.1|11980.m06527|protein Cys/Met metabolism PLP-dependent enzyme family protein, expressed
8,3	1099	LOC_Os10g37110.1|11980.m06511|protein transposon protein, putative, unclassified, expressed
2,2	34820	LOC_Os10g36550.2|11980.m50396|protein expressed protein
2,1	11329	LOC_Os10g36470.2|11980.m06457|protein methionine aminopeptidase 1, putative, expressed
3,1	10751	LOC_Os10g35630.2|11980.m06383|protein CBS domain-containing protein, putative, expressed
2,1	702	LOC_Os10g35220.2|11980.m21929|protein KH domain containing protein, expressed

7,5	30588	LOC_Os10g35050.1|11980.m06328|protein Aquaporin TIP3.1, putative, expressed
2,6	4282	LOC_Os10g34614.1|11980.m06284|protein expressed protein
4,3	24308	LOC_Os10g34602.1|11980.m06283|protein csAtPR5, putative, expressed
3,5	6663	LOC_Os10g33420.1|11980.m06170|protein expressed protein
2,5	8516	LOC_Os10g33420.1|11980.m06170|protein expressed protein
2,1	25878	LOC_Os10g33420.1|11980.m06170|protein expressed protein
11,4	23041	LOC_Os10g30450.1|11980.m05907|protein heavy metal-associated domain containing protein, expressed
5,9	22190	LOC_Os10g28200.1|11980.m05702|protein NAD dependent epimerase/dehydratase family protein, expressed
3,3	10133	LOC_Os10g28200.1|11980.m05702|protein NAD dependent epimerase/dehydratase family protein, expressed
11,7	4359	LOC_Os10g26600.1|11980.m05599|protein Soluble inorganic pyrophosphatase, putative, expressed
2,1	37375	LOC_Os10g26560.1|11980.m05596|protein expressed protein
2,4	7	LOC_Os10g20250.1|11980.m05046|protein expressed protein
4,0	31613	LOC_Os10g17770.1|11980.m04861|protein chromdomain-containing protein CRD101, putative, expressed
2,5	4770	LOC_Os10g12130.1|11980.m04527|protein expressed protein
2,2	9317	LOC_Os10g11810.2|11980.m21842|protein mannitol dehydrogenase, putative, expressed
2,9	23974	LOC_Os10g04700.2|11980.m03880|protein F-box domain containing protein, expressed
3,7	32483	LOC_Os10g04620.1|11980.m03874|protein hydrolase, alpha/beta fold family protein, putative, expressed
4,2	27568	LOC_Os09g39870.1|11979.m06924|protein 2-phosphoglycerate kinase, putative, expressed
2,4	36065	LOC_Os09g39462.3|11979.m22225|protein expressed protein
2,6	12483	LOC_Os09g39440.1|11979.m06882|protein Inosine-uridine preferring nucleoside hydrolase family protein, expressed
2,2	23459	LOC_Os09g39440.1|11979.m06882|protein Inosine-uridine preferring nucleoside hydrolase family protein, expressed
4,2	32217	LOC_Os09g39310.1|11979.m06870|protein expressed protein
3,7	4759	LOC_Os09g39190.1|11979.m06858|protein Copine family protein, expressed
2,7	5830	LOC_Os09g36920.1|11979.m06641|protein La domain containing protein, expressed
5,6	1086	LOC_Os09g36800.1|11979.m06629|protein 3-dehydroquinate synthase, putative, expressed
2,1	4087	LOC_Os09g36800.1|11979.m06629|protein 3-dehydroquinate synthase, putative, expressed
2,5	29675	LOC_Os09g34070.2|11979.m22168|protein RNA recognition motif family protein, expressed
2,3	20112	LOC_Os09g33690.1|11979.m06418|protein retrotransposon protein, putative, unclassified, expressed
14,2	15663	LOC_Os09g32840.1|11979.m06370|protein Metallophosphatase, putative, expressed
10,5	11709	LOC_Os09g32840.1|11979.m06370|protein Metallophosphatase, putative, expressed
2,7	11875	LOC_Os09g32810.1|11979.m06367|protein Ribulose-phosphate 3-epimerase, cytoplasmic isoform, putative, expressed
8,4	11298	LOC_Os09g32650.1|11979.m06352|protein leucyl-tRNA synthetase family protein, expressed
5,0	18943	LOC_Os09g32650.1|11979.m06352|protein leucyl-tRNA synthetase family protein, expressed
2,0	501	LOC_Os09g32050.2|11979.m22147|protein Chaperone protein dnaJ 10, putative, expressed
12,6	22129	LOC_Os09g32040.1|11979.m06294|protein No apical meristem protein, expressed
7,6	36980	LOC_Os09g31970.1|11979.m06287|protein 3-hydroxy-3-methylglutaryl-coenzyme A reductase 3, putative, expressed
6,3	18543	LOC_Os09g31486.2|11979.m22144|protein Heat shock 70 kDa protein, mitochondrial precursor, putative, expressed
2,0	25958	LOC_Os09g31486.2|11979.m22144|protein Heat shock 70 kDa protein, mitochondrial precursor, putative, expressed
2,6	392	LOC_Os09g31486.1|11979.m06271|protein Heat shock 70 kDa protein, mitochondrial precursor, putative, expressed
3,6	9763	LOC_Os09g30220.1|11979.m06173|protein NB-ARC domain containing protein, expressed
2,7	7274	LOC_Os09g28810.1|11979.m06032|protein RNA recognition motif family protein, expressed
4,9	11803	LOC_Os09g28019.1|11979.m05952|protein nucleoporin, putative, expressed
4,3	665	LOC_Os09g27210.1|11979.m05873|protein lecithine cholesterol acyltransferase, putative, expressed
17,3	22721	LOC_Os09g27050.1|11979.m05857|protein RSH2, putative, expressed
17,2	26108	LOC_Os09g27050.1|11979.m05857|protein RSH2, putative, expressed
4,7	33886	LOC_Os09g27050.1|11979.m05857|protein RSH2, putative, expressed
56,6	31409	LOC_Os09g26144.1|11979.m05766|protein Receptor family ligand binding region containing protein, expressed
7,2	5300	LOC_Os09g24970.1|11979.m05651|protein expressed protein
4,4	25634	LOC_Os09g24230.1|11979.m05579|protein haloacid dehalogenase-like hydrolase family protein, putative, expressed

3,7	26348	LOC_Os09g23740.1|11979.m05530|protein Phosphatidylinositol-4-phosphate 5-Kinase family protein, expressed
3,3	27649	LOC_Os09g23690.1|11979.m05525|protein UBX domain containing protein, expressed
4,0	37516	LOC_Os09g23660.2|11979.m22234|protein ZIM motif family protein, expressed
2,5	14046	LOC_Os09g23660.2|11979.m22234|protein ZIM motif family protein, expressed
2,4	36366	LOC_Os09g23660.1|11979.m05522|protein ZIM motif family protein, expressed
5,6	36808	LOC_Os09g23560.1|11979.m05513|protein oxidoreductase, zinc-binding dehydrogenase family protein, expressed
2,0	20133	LOC_Os09g23300.2|11979.m05487|protein Integral membrane protein, expressed
2,3	34701	LOC_Os09g22440.1|11979.m05450|protein expressed protein
4,4	3437	LOC_Os09g21110.1|11979.m05318|protein leucyl-tRNA synthetase family protein, expressed
6,2	16532	LOC_Os09g17840.1|11979.m05042|protein Protein transport protein Sec61 alpha subunit isoform 2
3,7	119	LOC_Os09g17840.1|11979.m05042|protein Protein transport protein Sec61 alpha subunit isoform 2
2,9	32118	LOC_Os09g17750.1|11979.m05033|protein oxidoreductase, short chain dehydrogenase/reductase family protein
4,6	18670	LOC_Os09g17070.1|11979.m04967|protein hypothetical protein
3,0	6598	LOC_Os09g15770.1|11979.m04838|protein Eukaryotic translation initiation factor 5, putative, expressed
12,0	30837	LOC_Os09g14670.1|11979.m04730|protein Phosphoenolpyruvate carboxylase 2, putative, expressed
8,1	6876	LOC_Os09g14670.1|11979.m04730|protein Phosphoenolpyruvate carboxylase 2, putative, expressed
2,1	29000	LOC_Os09g14670.1|11979.m04730|protein Phosphoenolpyruvate carboxylase 2, putative, expressed
5,7	14411	LOC_Os09g12560.1|11979.m22209|protein Ferrochelatase II, chloroplast precursor, putative, expressed
4,3	13960	LOC_Os09g12560.1|11979.m22209|protein Ferrochelatase II, chloroplast precursor, putative, expressed
3,7	36661	LOC_Os09g12560.1|11979.m22209|protein Ferrochelatase II, chloroplast precursor, putative, expressed
10,2	20581	LOC_Os09g08072.1|11979.m04076|protein Beta-fructofuranosidase, insoluble isoenzyme 7 precursor
2,2	23078	LOC_Os09g06464.1|11979.m03916|protein CCT motif family protein, expressed
3,6	32422	LOC_Os09g04160.1|11979.m03785|protein expressed protein
2,9	28863	LOC_Os09g03610.2|11979.m21962|protein FCA protein, putative, expressed
5,5	22200	LOC_Os09g02530.1|11979.m03625|protein expressed protein
3,8	6005	LOC_Os09g02270.1|11979.m03600|protein cyclase family protein, putative, expressed
2,8	2908	LOC_Os08g45080.1|11978.m08478|protein expressed protein
4,3	6729	LOC_Os08g44820.3|11978.m26799|protein No apical meristem protein, expressed
5,4	33057	LOC_Os08g44370.2|11978.m26790|protein Inosine-uridine preferring nucleoside hydrolase family protein, expressed
3,6	13294	LOC_Os08g44370.2|11978.m26790|protein Inosine-uridine preferring nucleoside hydrolase family protein, expressed
2,2	33608	LOC_Os08g44370.1|11978.m08410|protein Inosine-uridine preferring nucleoside hydrolase family protein, expressed
13,2	2785	LOC_Os08g44340.1|11978.m08407|protein Monodehydroascorbate reductase, putative, expressed
6,1	657	LOC_Os08g44340.1|11978.m08407|protein Monodehydroascorbate reductase, putative, expressed
5,4	8354	LOC_Os08g44100.1|11978.m08384|protein Endomembrane protein 70 containing protein, expressed
3,4	22423	LOC_Os08g43180.1|11978.m08292|protein expressed protein
2,1	19528	LOC_Os08g41110.1|11978.m08089|protein Chaperone protein dnaJ 10, putative, expressed
2,3	3242	LOC_Os08g41010.7|11978.m26752|protein Zn-finger in Ran binding protein and others containing protein, expressed
3,3	36590	LOC_Os08g40620.1|11978.m08041|protein RabGAP/TBC domain-containing protein, putative, expressed
4,7	31830	LOC_Os08g40590.1|11978.m08038|protein Oxysterol-binding protein, expressed
3,6	33611	LOC_Os08g40530.2|11978.m61978|protein Calcium-transporting ATPase 9, plasma membrane-type, putative, expressed
3,3	4201	LOC_Os08g39980.1|11978.m07978|protein Bromodomain containing protein, expressed
2,8	25568	LOC_Os08g39350.1|11978.m07916|protein Glycerophosphoryl diester phosphodiesterase family protein, expressed
8,2	32081	LOC_Os08g39150.1|11978.m07897|protein expressed protein
2,2	28928	LOC_Os08g39150.1|11978.m07897|protein expressed protein
3,9	27738	LOC_Os08g38570.1|11978.m07841|protein transducin family protein, putative, expressed
4,2	32340	LOC_Os08g37444.5|11978.m62017|protein Signal recognition particle, alpha subunit, N-terminal family protein, expressed
2,8	12603	LOC_Os08g37370.1|11978.m07723|protein Mitochondrial carrier protein, expressed
3,1	5983	LOC_Os08g35480.1|11978.m07537|protein Protein of unknown function containing protein, expressed
6,4	24494	LOC_Os08g33720.1|11978.m07362|protein Malate dehydrogenase, mitochondrial precursor, putative, expressed

2,2	14825	LOC_Os08g31940.1|11978.m07188|protein expressed protein
89,5	21986	LOC_Os08g31170.1|11978.m07115|protein C1-like domain containing protein
4,0	5030	LOC_Os08g30150.1|11978.m07017|protein oxidoreductase, 2OG-Fe oxygenase family protein, expressed
2,6	1913	LOC_Os08g23110.1|11978.m06332|protein tRNA synthetases class I family protein, expressed
5,5	26741	LOC_Os08g19170.1|11978.m05987|protein G-patch domain containing protein, expressed
6,6	12461	LOC_Os08g16480.1|11978.m05770|protein ATPase, AFG1 family protein, expressed
4,2	20963	LOC_Os08g16480.1|11978.m05770|protein ATPase, AFG1 family protein, expressed
3,5	1122	LOC_Os08g16480.1|11978.m05770|protein ATPase, AFG1 family protein, expressed
2,1	16359	LOC_Os08g13080.1|11978.m05435|protein retrotransposon protein, putative, unclassified
5,7	8664	LOC_Os08g12890.1|11978.m05416|protein expressed protein
2,9	24524	LOC_Os08g10480.1|11978.m05181|protein copper chaperone, putative, expressed
2,3	34486	LOC_Os08g10480.1|11978.m05181|protein copper chaperone, putative, expressed
3,2	21037	LOC_Os08g09200.1|11978.m05055|protein Aconitate hydratase, cytoplasmic, putative, expressed
2,8	5837	LOC_Os08g06810.1|11978.m04820|protein hypothetical protein
3,8	25415	LOC_Os08g06140.3|11978.m26586|protein No apical meristem protein, expressed
3,0	2267	LOC_Os08g04130.1|11978.m04557|protein Copine family protein, expressed
2,9	4795	LOC_Os08g01880.1|11978.m04336|protein synaptobrevin-related family protein, putative, expressed
3,3	7781	LOC_Os08g01760.2|11978.m26554|protein oxidoreductase, zinc-binding dehydrogenase family protein, expressed
9,8	34097	LOC_Os08g01620.2|11978.m26551|protein Shwachman-Bodian-Diamond syndrome protein homolog, putative, expressed
2,4	35380	LOC_Os08g01620.2|11978.m26551|protein Shwachman-Bodian-Diamond syndrome protein homolog, putative, expressed
3,3	2378	LOC_Os08g01390.1|11978.m04288|protein Phosphatidylinositol-4-phosphate 5-Kinase family protein, expressed
2,8	25296	LOC_Os07g49520.1|11977.m09160|protein 2-oxoglutarate dehydrogenase, E1 component family protein, expressed
2,0	17159	LOC_Os07g49270.1|11977.m09136|protein AMP deaminase, putative, expressed
4,2	11014	LOC_Os07g49050.1|11977.m09114|protein hypothetical protein
7,4	3253	LOC_Os07g48890.1|11977.m09098|protein Poly polymerase central domain containing protein, expressed
4,8	2154	LOC_Os07g48760.1|11977.m09086|protein CIPK-like protein 1, putative, expressed
2,6	9719	LOC_Os07g47670.1|11977.m08982|protein Hypoxia induced protein conserved region containing protein, expressed
2,3	19699	LOC_Os07g47670.1|11977.m08982|protein Hypoxia induced protein conserved region containing protein, expressed
4,0	28407	LOC_Os07g47350.2|11977.m08952|protein Potassium transporter 7, putative, expressed
2,2	4570	LOC_Os07g47350.2|11977.m08952|protein Potassium transporter 7, putative, expressed
5,2	23453	LOC_Os07g47100.2|11977.m29017|protein sodium/hydrogen exchanger 3 family protein, expressed
5,1	23557	LOC_Os07g46160.1|11977.m08834|protein speckle-type POZ protein, putative, expressed
3,4	11530	LOC_Os07g45070.3|11977.m66697|protein FAT domain-containing protein, putative, expressed
3,7	1012	LOC_Os07g44950.1|11977.m28925|protein bZIP transcription factor family protein, expressed
3,1	27085	LOC_Os07g44950.1|11977.m28925|protein bZIP transcription factor family protein, expressed
7,0	35532	LOC_Os07g44820.1|11977.m08703|protein expressed protein
4,7	2114	LOC_Os07g44820.1|11977.m08703|protein expressed protein
2,1	23615	LOC_Os07g44430.2|11977.m29285|protein peroxiredoxin, putative, expressed
2,9	11494	LOC_Os07g43010.1|11977.m08530|protein expressed protein
2,2	14420	LOC_Os07g42500.1|11977.m08482|protein Senescence-associated, putative, expressed
5,0	32617	LOC_Os07g42370.2|11977.m29260|protein ZIM motif family protein, expressed
2,3	9422	LOC_Os07g42280.1|11977.m08463|protein von Willebrand factor type A domain containing protein, expressed
2,2	9436	LOC_Os07g42250.1|11977.m08460|protein Strictosidine synthase family protein, expressed
2,9	3035	LOC_Os07g41800.1|11977.m08416|protein Peroxisome assembly protein 10, putative, expressed
3,7	17197	LOC_Os07g40930.1|11977.m08333|protein WD-repeat protein 12, putative, expressed
4,6	36812	LOC_Os07g40290.1|11977.m08271|protein indole-3-acetic acid-amido synthetase GH3.1, putative, expressed
5,6	33528	LOC_Os07g39900.1|11977.m08232|protein Interferon-related developmental regulator family protein, expressed
4,1	26026	LOC_Os07g39280.1|11977.m08170|protein expressed protein
3,6	25984	LOC_Os07g39270.2|11977.m28997|protein Geranylgeranyl pyrophosphate synthetase, chloroplast precursor

4,5	19634	LOC_Os07g38120.1|11977.m08061|protein Calcium-dependent protein kinase, isoform AK1, putative, expressed
2,7	18932	LOC_Os07g37890.1|11977.m08038|protein protein phosphatase 2C family protein, putative, expressed
2,5	9991	LOC_Os07g37650.1|11977.m08015|protein GTPase activating protein, putative, expressed
2,5	18900	LOC_Os07g36590.1|11977.m07910|protein KI domain interacting kinase 1, putative, expressed
2,0	22087	LOC_Os07g36420.1|11977.m07893|protein proteasome inhibitor, putative, expressed
2,3	27067	LOC_Os07g34740.1|11977.m07730|protein expressed protein
2,0	18152	LOC_Os07g34740.1|11977.m07730|protein expressed protein
6,8	8177	LOC_Os07g34006.1|11977.m07659|protein Glucose-6-phosphate/phosphate translocator 2, chloroplast precursor, putative
3,0	13438	LOC_Os07g34006.1|11977.m07659|protein Glucose-6-phosphate/phosphate translocator 2, chloroplast precursor, putative
13,8	30120	LOC_Os07g32570.1|11977.m07520|protein 5'-adenylylsulfate reductase 2, chloroplast precursor, putative, expressed
3,9	12292	LOC_Os07g32570.1|11977.m07520|protein 5'-adenylylsulfate reductase 2, chloroplast precursor, putative, expressed
6,9	29591	LOC_Os07g32380.1|11977.m07503|protein Protein phosphatase 2C containing protein, expressed
5,3	13023	LOC_Os07g32380.1|11977.m07503|protein Protein phosphatase 2C containing protein, expressed
3,3	18633	LOC_Os07g32380.1|11977.m07503|protein Protein phosphatase 2C containing protein, expressed
2,9	16823	LOC_Os07g31830.1|11977.m07449|protein expressed protein
2,0	37801	LOC_Os07g31830.1|11977.m07449|protein expressed protein
3,7	25565	LOC_Os07g30300.2|11977.m29178|protein small G protein family protein, putative, expressed
2,1	11968	LOC_Os07g28820.1|11977.m07153|protein Ribonuclease 2-5A family protein, expressed
6,5	18939	LOC_Os07g27790.1|11977.m07053|protein Glutamate-cysteine ligase, chloroplast precursor, putative, expressed
4,1	16513	LOC_Os07g27790.1|11977.m07053|protein Glutamate-cysteine ligase, chloroplast precursor, putative, expressed
2,1	36789	LOC_Os07g27370.1|11977.m07012|protein NB-ARC domain containing protein, expressed
4,1	34448	LOC_Os07g26490.1|11977.m06926|protein expressed protein
2,2	9688	LOC_Os07g23900.1|11977.m06668|protein F-box domain containing protein, expressed
2,8	2337	LOC_Os07g22710.2|11977.m29137|protein CDPK-related protein kinase, putative, expressed
3,5	8819	LOC_Os07g17130.1|11977.m06154|protein Senescence-associated, putative, expressed
2,1	9329	LOC_Os07g12900.1|11977.m05742|protein cadmium-translocating P-type ATPase family protein, expressed
2,0	5853	LOC_Os07g12710.1|11977.m05723|protein GIY-YIG catalytic domain containing protein, expressed
2,8	28301	LOC_Os07g09010.1|11977.m05357|protein nodulin family protein, putative, expressed
2,1	5846	LOC_Os07g09010.1|11977.m05357|protein nodulin family protein, putative, expressed
2,2	16007	LOC_Os07g08970.1|11977.m05353|protein expressed protein
2,8	2941	LOC_Os07g08950.2|11977.m05351|protein D-lactate dehydrogenase 2, mitochondrial precursor, putative, expressed
2,5	34996	LOC_Os07g08950.1|11977.m29086|protein D-lactate dehydrogenase 2, mitochondrial precursor, putative, expressed
3,8	25593	LOC_Os07g07194.2|11977.m66713|protein MAC/Perforin domain containing protein, expressed
9,9	22007	LOC_Os07g07194.1|11977.m05178|protein MAC/Perforin domain containing protein, expressed
4,5	12044	LOC_Os07g05620.2|11977.m29069|protein CIPK-like protein 1, putative, expressed
5,4	14591	LOC_Os07g04210.1|11977.m04890|protein calcineurin-like phosphoesterase family protein, putative, expressed
5,4	16183	LOC_Os07g03240.1|11977.m04795|protein RNA recognition motif family protein, expressed
2,3	12910	LOC_Os07g03240.1|11977.m04795|protein RNA recognition motif family protein, expressed
2,1	15715	LOC_Os07g03100.2|11977.m29050|protein F-box domain containing protein, expressed
4,3	3327	LOC_Os07g02460.1|11977.m04718|protein expressed protein
2,5	12385	LOC_Os07g02300.2|11977.m29043|protein TPR Domain containing protein, expressed
2,5	19001	LOC_Os07g01850.1|11977.m04663|protein NLI interacting factor-like phosphatase family protein, expressed
6,2	14590	LOC_Os06g51390.2|11976.m32211|protein expressed protein
5,7	8529	LOC_Os06g51360.2|11976.m32210|protein LysM domain containing protein, expressed
2,1	29465	LOC_Os06g50180.1|11976.m09542|protein Non-imprinted in Prader-Willi/Angelman syndrome region protein 2homolog
2,6	30264	LOC_Os06g50030.1|11976.m09527|protein CDPK-related protein kinase, putative, expressed
2,3	5828	LOC_Os06g49890.1|11976.m09513|protein mitochondrion protein, putative, expressed
5,1	18377	LOC_Os06g48770.2|11976.m32168|protein beta-lactamase family protein, expressed
2,9	36130	LOC_Os06g48770.2|11976.m32168|protein beta-lactamase family protein, expressed

3,3	9389	LOC_Os06g48350.1|11976.m09361|protein Eukaryotic translation initiation factor 5, putative, expressed
2,4	8488	LOC_Os06g48320.1|11976.m09358|protein pleckstrin homology domain-containing protein, putative, expressed
3,5	16934	LOC_Os06g47860.2|11976.m31919|protein expressed protein
2,6	35757	LOC_Os06g47860.2|11976.m31919|protein expressed protein
2,4	29896	LOC_Os06g46820.1|11976.m09208|protein expressed protein
3,2	33848	LOC_Os06g46240.1|11976.m09150|protein BTB/POZ domain containing protein, expressed
3,0	30244	LOC_Os06g45860.1|11976.m09114|protein molybdenum cofactor sulfurase, putative, expressed
7,6	14929	LOC_Os06g45110.1|11976.m09038|protein expressed protein
3,3	27000	LOC_Os06g43760.1|11976.m08906|protein isoleucyl-tRNA synthetase, putative, expressed
2,1	37665	LOC_Os06g43760.1|11976.m08906|protein isoleucyl-tRNA synthetase, putative, expressed
4,6	28138	LOC_Os06g42490.1|11976.m08780|protein expressed protein
3,4	18484	LOC_Os06g38320.2|11976.m31886|protein expressed protein
9,5	4150	LOC_Os06g37620.1|11976.m08297|protein U-box domain containing protein, expressed
7,3	11365	LOC_Os06g37620.1|11976.m08297|protein U-box domain containing protein, expressed
7,5	28508	LOC_Os06g36920.1|11976.m08227|protein thromboxane-A synthase, putative, expressed
2,2	11949	LOC_Os06g35900.1|11976.m08127|protein BES1/BZR1 homolog protein 4, putative, expressed
2,1	594	LOC_Os06g33710.2|11976.m32093|protein Spermidine synthase 2, putative, expressed
4,6	29405	LOC_Os06g31210.1|11976.m07662|protein methionyl-tRNA synthetase, putative, expressed
2,6	10000	LOC_Os06g27770.1|11976.m07322|protein Isoflavone reductase homolog, putative, expressed
3,7	35223	LOC_Os06g24704.1|11976.m07120|protein Acyl-coenzyme A oxidase 3, peroxisomal precursor, putative, expressed
2,4	27548	LOC_Os06g24704.1|11976.m07120|protein Acyl-coenzyme A oxidase 3, peroxisomal precursor, putative, expressed
4,6	13460	LOC_Os06g23530.1|11976.m07005|protein Pre-mRNA splicing factor ATP-dependent RNA helicase, putative, expressed
3,6	37035	LOC_Os06g23290.4|11976.m32075|protein phosphatidylinositol 3- and 4-kinase family protein, putative, expressed
2,8	19225	LOC_Os06g23290.4|11976.m32075|protein phosphatidylinositol 3- and 4-kinase family protein, putative, expressed
2,9	30180	LOC_Os06g22140.1|11976.m06869|protein Thioredoxin reductase 1, putative, expressed
2,2	4028	LOC_Os06g20390.1|11976.m06696|protein expressed protein
3,0	32540	LOC_Os06g19690.1|11976.m06628|protein expressed protein
7,5	6214	LOC_Os06g19370.2|11976.m32049|protein expressed protein
4,6	12725	LOC_Os06g19010.1|11976.m06564|protein expressed protein
5,9	36524	LOC_Os06g15990.2|11976.m71425|protein Aldehyde dehydrogenase, mitochondrial precursor, putative, expressed
3,1	25117	LOC_Os06g15990.2|11976.m71425|protein Aldehyde dehydrogenase, mitochondrial precursor, putative, expressed
4,4	10349	LOC_Os06g14750.1|11976.m06190|protein Phosphatidylinositol-4-phosphate 5-Kinase family protein, expressed
5,0	28761	LOC_Os06g13220.1|11976.m06037|protein axi 1 gene, putative, expressed
2,2	32699	LOC_Os06g13080.1|11976.m06023|protein U-box domain containing protein, expressed
12,9	17219	LOC_Os06g12390.1|11976.m05956|protein Galactoside-binding lectin family protein, expressed
2,1	10411	LOC_Os06g12390.1|11976.m05956|protein Galactoside-binding lectin family protein, expressed
3,1	1836	LOC_Os06g12100.1|11976.m05927|protein mTERF family protein, expressed
2,5	4090	LOC_Os06g12100.1|11976.m05927|protein mTERF family protein, expressed
2,2	29786	LOC_Os06g12030.3|11976.m32021|protein Domain found in Dishevelled, Egl-10, and Pleckstrin family protein, expressed
3,0	32486	LOC_Os06g10520.1|11976.m05770|protein pantothenate kinase family protein, expressed
2,9	2042	LOC_Os06g10520.1|11976.m05770|protein pantothenate kinase family protein, expressed
10,6	9878	LOC_Os06g10210.2|11976.m05739|protein expressed protein
6,5	26147	LOC_Os06g09450.4|11976.m31855|protein Sucrose synthase 1, putative, expressed
2,9	364	LOC_Os06g08740.2|11976.m31850|protein expressed protein
2,3	20051	LOC_Os06g08400.2|11976.m32223|protein LIM domain-containing protein, putative, expressed
3,3	24606	LOC_Os06g08060.1|11976.m05528|protein oxidoreductase, 2OG-Fe oxygenase family protein, expressed
6,8	17223	LOC_Os06g08032.1|11976.m05526|protein oxidoreductase, 2OG-Fe oxygenase family protein
2,3	31078	LOC_Os06g05700.1|11976.m05296|protein Cysteine synthase, putative, expressed
2,3	34619	LOC_Os06g05240.1|11976.m05253|protein Zinc carboxypeptidase family protein, expressed

2,3	20955	LOC_Os06g04880.2|11976.m31972|protein U-box domain containing protein, expressed
9,2	26376	LOC_Os06g04280.2|11976.m31970|protein 3-phosphoshikimate 1-carboxyvinyltransferase, chloroplast precursor
4,9	15962	LOC_Os06g04280.2|11976.m31970|protein 3-phosphoshikimate 1-carboxyvinyltransferase, chloroplast precursor
3,9	9195	LOC_Os06g02540.1|11976.m04987|protein expressed protein
2,9	4392	LOC_Os06g02240.1|11976.m04958|protein RNA recognition motif, putative, expressed
13,2	1216	LOC_Os06g02144.1|11976.m04949|protein 6-phosphogluconate dehydrogenase, decarboxylating, putative, expressed
4,5	4555	LOC_Os06g02144.1|11976.m04949|protein 6-phosphogluconate dehydrogenase, decarboxylating, putative, expressed
3,9	10856	LOC_Os06g02144.1|11976.m04949|protein 6-phosphogluconate dehydrogenase, decarboxylating, putative, expressed
3,2	2506	LOC_Os06g02144.1|11976.m04949|protein 6-phosphogluconate dehydrogenase, decarboxylating, putative, expressed
2,5	20589	LOC_Os06g02144.1|11976.m04949|protein 6-phosphogluconate dehydrogenase, decarboxylating, putative, expressed
4,6	21594	LOC_Os06g01304.2|11976.m71404|protein Spotted leaf protein 11, putative, expressed
2,1	37497	LOC_Os05g50920.1|11975.m09159|protein amino acid permease, putative, expressed
3,8	12611	LOC_Os05g50710.1|11975.m09138|protein Late embryogenesis abundant protein Lea14-A, putative, expressed
3,6	27907	LOC_Os05g49020.1|11975.m08973|protein hypothetical protein
3,2	26236	LOC_Os05g48930.1|11975.m08964|protein Glutaredoxin-like family protein, expressed
6,7	1134	LOC_Os05g48800.1|11975.m08951|protein fiber protein Fb2, putative, expressed
2,3	27322	LOC_Os05g47630.1|11975.m08835|protein peptidyl-tRNA hydrolase family protein, expressed
2,1	6441	LOC_Os05g45930.1|11975.m08718|protein IQ calmodulin-binding motif family protein, expressed
2,7	29909	LOC_Os05g45350.2|11975.m08660|protein DnaJ domain containing protein, expressed
3,9	21265	LOC_Os05g44922.1|11975.m08617|protein phosphofructokinase family protein, putative, expressed
2,2	16231	LOC_Os05g43770.1|11975.m08503|protein NLI interacting factor-like phosphatase family protein, expressed
3,9	6832	LOC_Os05g42350.1|11975.m08408|protein Sulfite reductase, putative, expressed
2,3	29119	LOC_Os05g41970.1|11975.m08371|protein 19 kDa globulin precursor, putative, expressed
2,3	31929	LOC_Os05g41460.1|11975.m08321|protein Cysteine proteinase inhibitor-II, putative, expressed
3,2	20732	LOC_Os05g41220.1|11975.m08297|protein SNF1-related protein kinase regulatory beta subunit 1, putative, expressed
3,2	14461	LOC_Os05g41100.1|11975.m08286|protein Krr1 family protein, expressed
3,6	8639	LOC_Os05g40220.1|11975.m08198|protein retrotransposon protein, putative, unclassified, expressed
4,5	34873	LOC_Os05g39770.1|11975.m08155|protein Alanine-glyoxylate aminotransferase 2 homolog 3, mitochondrial precursor
2,3	35140	LOC_Os05g39770.1|11975.m08155|protein Alanine-glyoxylate aminotransferase 2 homolog 3, mitochondrial precursor
4,8	36863	LOC_Os05g39730.1|11975.m08151|protein Protein of unknown function, DUF614 containing protein, expressed
8,1	31717	LOC_Os05g38940.1|11975.m08073|protein expressed protein
3,5	2899	LOC_Os05g38150.2|11975.m27606|protein Delta 1-pyrroline-5-carboxylate synthetase, putative, expressed
5,3	23896	LOC_Os05g38040.1|11975.m27862|protein expressed protein
5,4	28898	LOC_Os05g37910.2|11975.m27858|protein phosphatidic acid phosphatase, putative, expressed
7,2	25836	LOC_Os05g37910.1|11975.m07970|protein phosphatidic acid phosphatase, putative, expressed
2,2	25210	LOC_Os05g37660.1|11975.m07945|protein AER, putative, expressed
2,1	29547	LOC_Os05g37410.1|11975.m07921|protein HhH-GPD superfamily base excision DNA repair protein, expressed
2,2	23310	LOC_Os05g37140.2|11975.m28058|protein Ferredoxin-6, chloroplast precursor, putative, expressed
2,6	19601	LOC_Os05g35650.1|11975.m07795|protein POT family protein, expressed
4,0	8370	LOC_Os05g32630.1|11975.m07496|protein Mitochondrial carrier protein, expressed
4,2	3096	LOC_Os05g31140.1|11975.m07348|protein Lichenase II precursor, putative, expressed
2,8	6399	LOC_Os05g30970.1|11975.m07332|protein Copine family protein, expressed
3,6	3133	LOC_Os05g29030.1|11975.m07189|protein protein phosphatase 2C, putative, expressed
2,7	179	LOC_Os05g28300.1|11975.m07116|protein expressed protein
2,4	15567	LOC_Os05g28300.1|11975.m07116|protein expressed protein
4,2	11242	LOC_Os05g27930.3|11975.m28049|protein AP2 domain containing protein, expressed
2,9	31529	LOC_Os05g27930.3|11975.m28049|protein AP2 domain containing protein, expressed
4,5	23835	LOC_Os05g27930.1|11975.m28050|protein AP2 domain containing protein, expressed
2,9	14904	LOC_Os05g25210.1|11975.m06814|protein expressed protein

5,0	17558	LOC_Os05g23600.2|11975.m27570|protein Ribophorin I family protein, expressed
3,3	29618	LOC_Os05g10810.1|11975.m05551|protein MBOAT family protein, expressed
7,2	34866	LOC_Os05g09440.1|11975.m05457|protein NADP-dependent malic enzyme, chloroplast precursor, putative, expressed
2,9	28968	LOC_Os05g08570.1|11975.m27719|protein AUX/IAA family protein, expressed
2,0	31631	LOC_Os05g08420.1|11975.m05359|protein expressed protein
3,2	28059	LOC_Os05g07700.4|11975.m28037|protein 60S ribosomal protein L10-3, putative, expressed
2,5	11160	LOC_Os05g07700.1|11975.m27462|protein 60S ribosomal protein L10-3, putative, expressed
2,4	30745	LOC_Os05g07090.1|11975.m05235|protein Acyl-coenzyme A oxidase 4, peroxisomal, putative, expressed
2,6	12209	LOC_Os05g06500.1|11975.m05177|protein universal stress protein family protein, expressed
2,2	10746	LOC_Os05g05940.1|11975.m05121|protein Rubber elongation factor protein containing protein, expressed
3,6	36452	LOC_Os05g05500.1|11975.m05077|protein F-box domain containing protein, expressed
2,1	25162	LOC_Os05g05354.1|11975.m05062|protein expressed protein
2,4	18025	LOC_Os05g05310.2|11975.m27699|protein Fibronectin type III domain containing protein, expressed
3,2	3753	LOC_Os05g04960.1|11975.m05025|protein phosphoglycerate mutase family protein, expressed
4,9	2024	LOC_Os05g03780.1|11975.m04912|protein cation diffusion facilitator family transporter containing protein, expressed
2,9	18272	LOC_Os05g03780.1|11975.m04912|protein cation diffusion facilitator family transporter containing protein, expressed
3,0	4568	LOC_Os05g03490.1|11975.m04883|protein expressed protein
2,5	12606	LOC_Os05g02990.2|11975.m64177|protein expressed protein
3,6	25223	LOC_Os05g02400.1|11975.m04776|protein RNA recognition motif family protein, expressed
2,3	18821	LOC_Os05g01490.1|11975.m04687|protein GTP-binding protein YPTM1, putative, expressed
2,5	26180	LOC_Os05g01250.1|11975.m04666|protein SNF7 family protein, expressed
2,5	36334	LOC_Os05g01240.4|11975.m28021|protein AML6, putative, expressed
7,3	28245	LOC_Os04g59550.3|11974.m35540|protein glucose-6-phosphate/phosphate translocator, putative, expressed
2,2	27833	LOC_Os04g59550.3|11974.m35540|protein glucose-6-phosphate/phosphate translocator, putative, expressed
2,1	17811	LOC_Os04g59550.3|11974.m35540|protein glucose-6-phosphate/phosphate translocator, putative, expressed
3,2	24962	LOC_Os04g59494.4|11974.m35538|protein importin beta-2 subunit family protein, putative, expressed
2,2	474	LOC_Os04g59140.1|11974.m10791|protein expressed protein
2,1	7720	LOC_Os04g58570.1|11974.m10739|protein C2 domain protein At1g63220, putative, expressed
4,4	1263	LOC_Os04g58390.1|11974.m10721|protein allantoinase, putative, expressed
4,6	1219	LOC_Os04g58090.1|11974.m10691|protein Harpin-induced protein 1 containing protein, expressed
3,6	1352	LOC_Os04g57810.2|11974.m35514|protein expressed protein
3,0	19928	LOC_Os04g57550.1|11974.m10640|protein amine oxidase, flavin-containing family protein, expressed
2,7	13617	LOC_Os04g57550.1|11974.m10640|protein amine oxidase, flavin-containing family protein, expressed
5,7	15385	LOC_Os04g56920.1|11974.m10579|protein Beta-fructofuranosidase, insoluble isoenzyme 6 precursor, putative, expressed
2,5	31003	LOC_Os04g55850.2|11974.m35598|protein S1/P1 Nuclease family protein, expressed
4,4	29357	LOC_Os04g55710.1|11974.m10462|protein transposon protein, putative, unclassified, expressed
3,0	24968	LOC_Os04g55520.1|11974.m10443|protein AP2 domain containing protein, expressed
3,8	16320	LOC_Os04g55410.1|11974.m10433|protein Glycerol kinase, putative, expressed
2,4	16223	LOC_Os04g54759.1|11974.m10366|protein expressed protein
4,1	31475	LOC_Os04g53810.1|11974.m10273|protein NAD dependent epimerase/dehydratase family protein, expressed
2,7	15082	LOC_Os04g53700.1|11974.m10263|protein MYND finger family protein, expressed
2,6	11855	LOC_Os04g52710.2|11974.m35459|protein Imidazoleglycerol-phosphate dehydratase, putative, expressed
4,0	22610	LOC_Os04g52540.1|11974.m10147|protein Piwi domain containing protein, expressed
2,6	23	LOC_Os04g52540.1|11974.m10147|protein Piwi domain containing protein, expressed
3,3	5482	LOC_Os04g52450.1|11974.m35066|protein aminotransferase, class III family protein, expressed
2,7	33347	LOC_Os04g52450.1|11974.m35066|protein aminotransferase, class III family protein, expressed
2,5	4204	LOC_Os04g52390.1|11974.m10133|protein Potassium transporter 11, putative, expressed
8,2	21623	LOC_Os04g52000.1|11974.m10095|protein Protein phosphatase 2C containing protein, expressed
7,9	1988	LOC_Os04g52000.1|11974.m10095|protein Protein phosphatase 2C containing protein, expressed

7,7	26699	LOC_Os04g52000.1|11974.m10095|protein Protein phosphatase 2C containing protein, expressed
2,4	27826	LOC_Os04g51090.1|11974.m10006|protein expressed protein
2,6	13779	LOC_Os04g50720.1|11974.m09969|protein RWD domain containing protein, expressed
4,7	28371	LOC_Os04g50120.1|11974.m09944|protein expressed protein
5,4	7519	LOC_Os04g50060.1|11974.m09938|protein GRAS family transcription factor containing protein, expressed
3,2	25492	LOC_Os04g50060.1|11974.m09938|protein GRAS family transcription factor containing protein, expressed
5,4	23901	LOC_Os04g49510.1|11974.m09884|protein Calcium-dependent protein kinase, isoform 11, putative, expressed
2,1	9319	LOC_Os04g48130.1|11974.m09751|protein membrane protein, putative, expressed
7,2	10549	LOC_Os04g44670.1|11974.m09426|protein AP2 domain containing protein
4,2	15435	LOC_Os04g44510.1|11974.m09410|protein GRAM domain-containing protein, putative, expressed
4,1	8748	LOC_Os04g43690.2|11974.m35383|protein expressed protein
5,3	33684	LOC_Os04g43650.2|11974.m35382|protein aminotransferase, classes I and II family protein, expressed
4,0	5027	LOC_Os04g43650.1|11974.m09324|protein aminotransferase, classes I and II family protein, expressed
3,8	27533	LOC_Os04g43650.1|11974.m09324|protein aminotransferase, classes I and II family protein, expressed
2,2	30441	LOC_Os04g43650.1|11974.m09324|protein aminotransferase, classes I and II family protein, expressed
2,3	31923	LOC_Os04g42930.1|11974.m09258|protein Glutaredoxin, putative, expressed
3,2	7957	LOC_Os04g41850.1|11974.m09159|protein RWP-RK domain containing protein, expressed
3,8	9407	LOC_Os04g40880.2|11974.m35347|protein Nucleotide-binding protein 1, putative, expressed
2,3	17226	LOC_Os04g40540.2|11974.m78963|protein Protein-L-isoaspartate O-methyltransferase, putative, expressed
3,6	34052	LOC_Os04g40520.1|11974.m09033|protein Raffinose synthase or seed imbibition protein Sip1 containing protein, expressed
2,2	31687	LOC_Os04g39420.1|11974.m08931|protein Pyrophosphate-fructose 6-phosphate 1-phosphotransferase, putative, expressed
3,5	3555	LOC_Os04g39270.2|11974.m35153|protein Indole-3-glycerol phosphate synthase, chloroplast precursor, putative, expressed
2,5	29167	LOC_Os04g39210.1|11974.m08910|protein Agmatine deiminase, putative, expressed
11,3	16944	LOC_Os04g38950.1|11974.m08884|protein Anthranilate synthase component II, putative, expressed
2,1	13247	LOC_Os04g38680.1|11974.m08857|protein Transmembrane amino acid transporter protein, expressed
5,8	15025	LOC_Os04g38540.1|11974.m35326|protein Aldose 1-epimerase family protein, expressed
2,1	35057	LOC_Os04g37904.1|11974.m08779|protein Protein phosphatase 2C containing protein, expressed
5,6	5795	LOC_Os04g37760.1|11974.m08765|protein expressed protein
2,9	33132	LOC_Os04g36630.1|11974.m08703|protein expressed protein
2,0	5436	LOC_Os04g35840.1|11974.m08673|protein T-complex protein 11 containing protein, expressed
2,8	33949	LOC_Os04g35170.1|11974.m08607|protein expressed protein
2,1	27702	LOC_Os04g35060.1|11974.m08597|protein nicotinate phosphoribosyltransferase, putative, expressed
3,7	34296	LOC_Os04g34940.1|11974.m08587|protein expressed protein
2,5	17081	LOC_Os04g32970.1|11974.m08394|protein OTU-like cysteine protease family protein, putative, expressed
2,3	16243	LOC_Os04g32970.1|11974.m08394|protein OTU-like cysteine protease family protein, putative, expressed
4,8	25862	LOC_Os04g32920.3|11974.m35132|protein Potassium transporter 1, putative, expressed
3,9	4400	LOC_Os04g32920.3|11974.m35132|protein Potassium transporter 1, putative, expressed
4,6	16522	LOC_Os04g31030.1|11974.m08207|protein Nitrate-induced NOI protein, expressed
2,5	32391	LOC_Os04g30890.1|11974.m08193|protein gonidia forming protein GlsA, putative, expressed
3,0	1716	LOC_Os04g24140.1|11974.m07548|protein ribose 5-phosphate isomerase, putative, expressed
2,4	36380	LOC_Os04g10400.1|11974.m06292|protein Electron transfer flavoprotein beta-subunit, putative, expressed
2,3	27876	LOC_Os04g10350.1|11974.m06287|protein oxidoreductase, 2OG-Fe oxygenase family protein, expressed
3,2	17581	LOC_Os04g08740.1|11974.m06161|protein GAF domain containing protein, expressed
6,2	25326	LOC_Os04g08350.1|11974.m06124|protein Cysteine synthase, chloroplast precursor, putative, expressed
2,4	27181	LOC_Os04g02910.3|11974.m35240|protein membrane related protein CP5, putative, expressed
18,1	21939	LOC_Os04g01310.1|11974.m05451|protein D-mannose binding lectin family protein, expressed
6,5	33574	LOC_Os04g01230.2|11974.m35229|protein phosphoglycerate mutase, putative, expressed
3,4	10712	LOC_Os03g63490.1|11973.m11217|protein Pantoate-beta-alanine ligase, putative, expressed
4,5	8850	LOC_Os03g62030.3|11973.m35510|protein expressed protein

4,1	24509	LOC_Os03g61990.4|11973.m35508|protein RNA recognition motif family protein, expressed
2,2	34604	LOC_Os03g61960.1|11973.m11075|protein Ferredoxin-3, chloroplast precursor, putative, expressed
2,5	34928	LOC_Os03g61810.1|11973.m11062|protein Guanine deaminase, putative, expressed
3,0	34060	LOC_Os03g60400.1|11973.m10930|protein 40S ribosomal protein S23, putative, expressed
3,1	18872	LOC_Os03g59470.1|11973.m10845|protein 5-azacytidine resistance protein, putative, expressed
2,7	18961	LOC_Os03g59470.1|11973.m10845|protein 5-azacytidine resistance protein, putative, expressed
2,4	22479	LOC_Os03g59180.2|11973.m10818|protein expressed protein
3,7	2850	LOC_Os03g59170.1|11973.m34717|protein adenosine 5'-phosphosulfate reductase 8, putative, expressed
3,6	12907	LOC_Os03g59080.1|11973.m10809|protein acyl-activating enzyme 18, putative, expressed
2,3	10797	LOC_Os03g58470.1|11973.m10753|protein linker histone H1 and H5 family protein, expressed
2,3	12654	LOC_Os03g58150.1|11973.m10727|protein Uncharacterized protein family UPF0005 containing protein, expressed
4,8	21288	LOC_Os03g58040.3|11973.m35454|protein Glutamate dehydrogenase, putative, expressed
3,9	25904	LOC_Os03g57430.1|11973.m10661|protein expressed protein
2,8	27710	LOC_Os03g57400.1|11973.m10658|protein hypothetical protein
4,1	23122	LOC_Os03g57340.1|11973.m10652|protein DnaJ protein homolog, putative, expressed
3,8	8894	LOC_Os03g57340.1|11973.m10652|protein DnaJ protein homolog, putative, expressed
3,2	14828	LOC_Os03g57340.1|11973.m10652|protein DnaJ protein homolog, putative, expressed
4,4	25975	LOC_Os03g57210.1|11973.m10640|protein expressed protein
3,7	17366	LOC_Os03g57050.1|11973.m10625|protein expressed protein
3,7	24388	LOC_Os03g56420.2|11973.m35584|protein dehydrogenase, putative, expressed
2,1	32126	LOC_Os03g56410.2|11973.m10550|protein expressed protein
3,2	2929	LOC_Os03g56250.1|11973.m10534|protein wound and phytochrome signaling involved receptor like kinase
4,7	19560	LOC_Os03g55704.1|11973.m10482|protein expressed protein
2,3	16741	LOC_Os03g55640.1|11973.m10476|protein signal peptidase I family protein, expressed
3,8	11611	LOC_Os03g53770.1|11973.m10346|protein RNA recognition motif family protein, expressed
20,4	6110	LOC_Os03g53230.1|11973.m10296|protein Bifunctional 3'-phosphoadenosine 5'-phosphosulfate synthethase
12,1	12215	LOC_Os03g53230.1|11973.m10296|protein Bifunctional 3'-phosphoadenosine 5'-phosphosulfate synthethase
4,2	6468	LOC_Os03g52470.1|11973.m10223|protein retrotransposon, putative, centromere-specific, expressed
4,9	19829	LOC_Os03g51430.1|11973.m10122|protein CRAL/TRIO domain containing protein, expressed
2,8	23792	LOC_Os03g51080.1|11973.m10089|protein Glutamate decarboxylase, putative, expressed
2,1	8640	LOC_Os03g51000.2|11973.m10082|protein 3,4-dihydroxy-2-butanone kinase, putative, expressed
4,4	34358	LOC_Os03g50490.1|11973.m10040|protein Glutamine synthetase root isozyme 2, putative, expressed
3,2	20158	LOC_Os03g50490.1|11973.m10040|protein Glutamine synthetase root isozyme 2, putative, expressed
10,0	24761	LOC_Os03g50450.1|11973.m10037|protein atypical receptor-like kinase MARK, putative, expressed
7,2	31570	LOC_Os03g50450.1|11973.m10037|protein atypical receptor-like kinase MARK, putative, expressed
4,6	6339	LOC_Os03g50280.1|11973.m10021|protein expressed protein
2,5	15588	LOC_Os03g49960.3|11973.m35385|protein expressed protein
2,2	9720	LOC_Os03g49770.2|11973.m09975|protein Oxysterol-binding protein, expressed
3,7	6253	LOC_Os03g49620.6|11973.m35380|protein BRASSINOSTEROID INSENSITIVE 1-associated receptor kinase 1 precursor
2,6	4653	LOC_Os03g49480.1|11973.m09947|protein GNS1/SUR4 family protein, expressed
2,2	8326	LOC_Os03g48660.1|11973.m09873|protein expressed protein
2,3	36269	LOC_Os03g45770.1|11973.m09602|protein S3 self-incompatibility locus-linked pollen 3.15 protein, putative, expressed
2,6	4027	LOC_Os03g44800.1|11973.m09515|protein expressed protein
4,9	7663	LOC_Os03g44780.1|11973.m09514|protein elongation factor Tu family protein, putative, expressed
2,7	33921	LOC_Os03g44530.1|11973.m09491|protein expressed protein
2,2	12213	LOC_Os03g44530.1|11973.m09491|protein expressed protein
3,8	18219	LOC_Os03g43720.5|11973.m35324|protein major facilitator superfamily protein, expressed
3,2	17578	LOC_Os03g43720.5|11973.m35324|protein major facilitator superfamily protein, expressed
2,1	316	LOC_Os03g43720.5|11973.m35324|protein major facilitator superfamily protein, expressed

2,9	1167	LOC_Os03g43340.1|11973.m09381|protein hypothetical protein
2,4	26841	LOC_Os03g43340.1|11973.m09381|protein hypothetical protein
3,5	8638	LOC_Os03g42760.1|11973.m09328|protein IBR domain containing protein, expressed
2,0	26849	LOC_Os03g42760.1|11973.m09328|protein IBR domain containing protein, expressed
3,3	19700	LOC_Os03g42320.1|11973.m09290|protein SEC1-family transport protein SLY1, putative, expressed
3,7	26615	LOC_Os03g42110.1|11973.m09269|protein N-acetyl-gamma-glutamyl-phosphate reductase, chloroplast precursor
2,6	5508	LOC_Os03g40670.1|11973.m09142|protein Glycerophosphoryl diester phosphodiesterase family protein, expressed
2,1	17458	LOC_Os03g40194.1|11973.m09103|protein DSBA-like thioredoxin domain containing protein, expressed
3,6	6939	LOC_Os03g39129.1|11973.m09003|protein expressed protein
2,3	30727	LOC_Os03g34300.2|11973.m35614|protein TMS membrane family protein, putative, expressed
5,6	12548	LOC_Os03g25750.1|11973.m07922|protein expressed protein
2,8	32169	LOC_Os03g25750.1|11973.m07922|protein expressed protein
3,8	26760	LOC_Os03g23010.2|11973.m07699|protein expressed protein
2,2	21854	LOC_Os03g23010.2|11973.m07699|protein expressed protein
2,4	15039	LOC_Os03g22810.2|11973.m35223|protein Superoxide dismutase 1, putative, expressed
2,9	6261	LOC_Os03g21080.1|11973.m07515|protein Guanine nucleotide exchange factor, putative, expressed
6,3	18822	LOC_Os03g21030.1|11973.m07510|protein GRAB2 protein, putative, expressed
4,3	9166	LOC_Os03g21000.1|11973.m07507|protein Thioredoxin-like 1, putative, expressed
2,7	29947	LOC_Os03g20790.1|11973.m07486|protein ETHYLENE-INSENSITIVE3-like 1 protein, putative, expressed
2,2	21019	LOC_Os03g20670.1|11973.m07474|protein phagocytosis and cell motility protein ELMO1, putative, expressed
2,2	2543	LOC_Os03g20380.6|11973.m35603|protein CIPK-like protein 1, putative, expressed
3,5	30771	LOC_Os03g20120.1|11973.m07424|protein galactinol synthase 3, putative, expressed
2,7	33565	LOC_Os03g19580.2|11973.m35183|protein expressed protein
2,9	32034	LOC_Os03g19400.1|11973.m07357|protein expressed protein
4,3	553	LOC_Os03g19260.1|11973.m07343|protein expressed protein
5,7	21834	LOC_Os03g19250.1|11973.m07342|protein AMP-binding enzyme family protein, expressed
5,2	30234	LOC_Os03g19040.1|11973.m07322|protein expressed protein
9,2	11387	LOC_Os03g18220.1|11973.m07247|protein Pyruvate decarboxylase isozyme 2, putative, expressed
5,0	9851	LOC_Os03g18220.1|11973.m07247|protein Pyruvate decarboxylase isozyme 2, putative, expressed
2,7	11898	LOC_Os03g18220.1|11973.m07247|protein Pyruvate decarboxylase isozyme 2, putative, expressed
3,2	5450	LOC_Os03g17950.1|11973.m07220|protein expressed protein
5,1	27279	LOC_Os03g17470.1|11973.m07174|protein IN2-1 protein, putative, expressed
3,0	27204	LOC_Os03g17120.1|11973.m07143|protein Arginine biosynthesis bifunctional protein argJ 1, putative, expressed
3,9	1338	LOC_Os03g16860.1|11973.m07118|protein Heat shock cognate 70 kDa protein, putative, expressed
10,4	27598	LOC_Os03g16700.1|11973.m07103|protein initiator-binding protein, putative, expressed
4,5	22869	LOC_Os03g16700.1|11973.m07103|protein initiator-binding protein, putative, expressed
3,9	36314	LOC_Os03g16690.1|11973.m07102|protein Oxysterol-binding protein, expressed
4,8	36472	LOC_Os03g14800.4|11973.m35124|protein aminotransferase, classes I and II family protein, expressed
2,1	14434	LOC_Os03g13840.2|11973.m34815|protein Senescence-associated protein, expressed
4,5	23116	LOC_Os03g13810.1|11973.m06825|protein expressed protein
5,2	35152	LOC_Os03g13590.1|11973.m06804|protein tropomyosin, putative, expressed
2,9	36408	LOC_Os03g13590.1|11973.m06804|protein tropomyosin, putative, expressed
2,4	8195	LOC_Os03g12910.1|11973.m06738|protein Squalene monooxygenase, putative, expressed
2,5	16477	LOC_Os03g12370.1|11973.m06687|protein HSF-type DNA-binding domain containing protein, expressed
8,5	33505	LOC_Os03g11590.3|11973.m34806|protein integral membrane family protein, putative, expressed
5,7	26122	LOC_Os03g10640.1|11973.m06516|protein Calcium-transporting ATPase 2, plasma membrane-type, putative, expressed
2,2	29914	LOC_Os03g10190.1|11973.m06472|protein Seryl-tRNA synthetase, putative, expressed
2,9	21753	LOC_Os03g09100.1|11973.m06416|protein anther ethylene-upregulated protein ER1, putative, expressed
6,0	16975	LOC_Os03g08910.1|11973.m06397|protein MatE family protein, expressed

3,1	25734	LOC_Os03g08700.1|11973.m06377|protein expressed protein
5,9	21755	LOC_Os03g08050.3|11973.m78808|protein Elongation factor 1-alpha, putative, expressed
5,7	24868	LOC_Os03g08050.3|11973.m78808|protein Elongation factor 1-alpha, putative, expressed
2,4	28432	LOC_Os03g07990.1|11973.m06308|protein GCN5-related N-acetyltransferase, putative, expressed
2,1	4589	LOC_Os03g06580.1|11973.m35029|protein expressed protein
3,2	16997	LOC_Os03g03510.2|11973.m34994|protein CIPK-like protein 1, putative, expressed
4,5	7812	LOC_Os03g03510.1|11973.m05884|protein CIPK-like protein 1, putative, expressed
6,8	21461	LOC_Os03g03450.1|11973.m05878|protein Anthranilate phosphoribosyltransferase, chloroplast precursor
7,3	35251	LOC_Os03g03034.2|11973.m78836|protein oxidoreductase, 2OG-Fe oxygenase family protein, expressed
3,1	19305	LOC_Os03g02840.1|11973.m05823|protein Remorin, C-terminal region family protein, expressed
5,0	25847	LOC_Os03g02514.2|11973.m35588|protein hydrolase, alpha/beta fold family protein, expressed
2,4	3978	LOC_Os03g02440.1|11973.m05786|protein expressed protein
2,0	9954	LOC_Os02g58640.1|11972.m10884|protein Tumour susceptibility gene 101 protein containing protein, expressed
7,4	123	LOC_Os02g58260.1|11972.m10847|protein metallo-beta-lactamase family protein, putative, expressed
2,1	9794	LOC_Os02g58220.1|11972.m10843|protein OB-fold nucleic acid binding domain containing protein, expressed
7,4	22285	LOC_Os02g57910.1|11972.m10812|protein F-box domain containing protein, expressed
3,0	10515	LOC_Os02g57650.1|11972.m10786|protein No apical meristem protein, expressed
2,1	23437	LOC_Os02g57260.1|11972.m10748|protein 3-ketoacyl-CoA thiolase 2, peroxisomal precursor, putative, expressed
10,3	17815	LOC_Os02g56900.1|11972.m10712|protein expressed protein
9,7	18705	LOC_Os02g56850.2|11972.m33473|protein Glutathione reductase, cytosolic, putative, expressed
3,7	14673	LOC_Os02g56850.2|11972.m33473|protein Glutathione reductase, cytosolic, putative, expressed
2,1	3014	LOC_Os02g56840.1|11972.m10706|protein F-box domain containing protein, expressed
5,4	10053	LOC_Os02g55970.1|11972.m10621|protein Clathrin assembly protein, putative, expressed
4,9	25076	LOC_Os02g55560.1|11972.m10582|protein DNA-binding protein phosphatase 2C, putative, expressed
2,5	35501	LOC_Os02g55470.1|11972.m10573|protein N-acetylglucosaminyltransferase II family protein, expressed
2,1	37445	LOC_Os02g54990.2|11972.m10526|protein TMS membrane family protein, putative, expressed
3,6	4982	LOC_Os02g54090.1|11972.m77855|protein expressed protein
6,4	9324	LOC_Os02g53700.2|11972.m33463|protein DENN domain containing protein, expressed
2,1	29543	LOC_Os02g53700.2|11972.m33463|protein DENN domain containing protein, expressed
4,8	25760	LOC_Os02g53490.1|11972.m10378|protein Metal tolerance protein C3, putative, expressed
6,5	24862	LOC_Os02g52560.1|11972.m10287|protein Xyloglucan fucosyltransferase family protein, expressed
5,3	35541	LOC_Os02g51410.1|11972.m10175|protein shikimate kinase, chloroplast precursor, putative, expressed
2,1	36271	LOC_Os02g50970.2|11972.m33450|protein Protein kinase domain containing protein, expressed
3,1	20144	LOC_Os02g50570.1|11972.m10091|protein transposon protein, putative, CACTA, En/Spm sub-class, expressed
2,2	375	LOC_Os02g50240.1|11972.m10058|protein Glutamine synthetase root isozyme 3, putative, expressed
5,0	2059	LOC_Os02g48790.3|11972.m33446|protein AML1, putative, expressed
4,4	28500	LOC_Os02g48560.2|11972.m33445|protein Omega-6 fatty acid desaturase, endoplasmic reticulum isozyme 2
2,5	9524	LOC_Os02g48190.1|11972.m09853|protein expressed protein
3,5	10652	LOC_Os02g48110.1|11972.m09845|protein dnaK protein, expressed
5,9	12049	LOC_Os02g47670.2|11972.m33442|protein U-box domain containing protein, expressed
3,1	6416	LOC_Os02g47670.2|11972.m33442|protein U-box domain containing protein, expressed
2,7	3052	LOC_Os02g47110.1|11972.m77840|protein ADP-ribosylation factor, putative, expressed
3,2	3783	LOC_Os02g46962.2|11972.m34031|protein VHS domain-containing protein, putative, expressed
4,0	24709	LOC_Os02g46830.1|11972.m09720|protein hypothetical protein
3,3	13022	LOC_Os02g44940.1|11972.m09534|protein Rapid ALkalinization Factor family protein, expressed
3,8	19272	LOC_Os02g44500.1|11972.m09490|protein phospholipid hydroperoxide glutathione peroxidase, putative, expressed
3,4	27671	LOC_Os02g44500.1|11972.m09490|protein phospholipid hydroperoxide glutathione peroxidase, putative, expressed
2,7	19684	LOC_Os02g43010.2|11972.m33796|protein Vacuolar processing enzyme, beta-isozyme precursor, putative, expressed
4,1	27379	LOC_Os02g42640.1|11972.m09299|protein expressed protein

2,1	32451	LOC_Os02g42350.1|11972.m09270|protein Nitrilase 4, putative, expressed
3,7	3977	LOC_Os02g42330.1|11972.m09268|protein Nitrilase 4, putative, expressed
2,8	2122	LOC_Os02g40700.1|11972.m09156|protein enzyme of the cupin superfamily, putative, expressed
2,0	9999	LOC_Os02g39920.1|11972.m09078|protein AT hook motif family protein, expressed
3,2	8060	LOC_Os02g39790.1|11972.m09065|protein S-adenosylmethionine decarboxylase proenzyme, putative, expressed
2,3	32538	LOC_Os02g39790.1|11972.m09065|protein S-adenosylmethionine decarboxylase proenzyme, putative, expressed
2,3	10309	LOC_Os02g39790.1|11972.m09065|protein S-adenosylmethionine decarboxylase proenzyme, putative, expressed
4,4	23781	LOC_Os02g39400.1|11972.m09028|protein isochorismatase family protein, expressed
2,2	19058	LOC_Os02g39160.1|11972.m09004|protein 4-hydroxy-3-methylbut-2-en-1-yl diphosphate synthase family protein
2,8	15893	LOC_Os02g38840.2|11972.m77828|protein Glucose-6-phosphate 1-dehydrogenase, cytoplasmic isoform, putative
2,1	13035	LOC_Os02g38300.1|11972.m08918|protein SNF7 family protein, expressed
2,4	15791	LOC_Os02g38140.1|11972.m08902|protein beta 1,4 N-acetylglucosaminyltransferase, putative, expressed
2,6	31920	LOC_Os02g38050.6|11972.m33744|protein PB1 domain containing protein, expressed
4,6	10936	LOC_Os02g37870.1|11972.m08875|protein Brix domain containing protein 2, putative, expressed
2,5	22270	LOC_Os02g35870.1|11972.m08675|protein Avr9 elicitor response protein, putative, expressed
2,2	3777	LOC_Os02g35190.2|11972.m33720|protein Chloride channel protein CLC-c, putative, expressed
2,6	1255	LOC_Os02g35130.1|11972.m08601|protein AIG1 family protein, expressed
2,3	20670	LOC_Os02g33080.2|11972.m33708|protein hydrolase, carbon-nitrogen family protein, expressed
3,7	7372	LOC_Os02g32110.1|11972.m08304|protein exostosin family protein, putative, expressed
6,7	33655	LOC_Os02g31910.2|11972.m33698|protein Potassium transporter 1, putative, expressed
2,2	13003	LOC_Os02g31100.1|11972.m08253|protein LITAF-domain-containing protein, putative, expressed
2,3	5813	LOC_Os02g27030.1|11972.m07847|protein Cysteine proteinase 1 precursor, putative, expressed
2,5	21744	LOC_Os02g25580.2|11972.m33674|protein SNARE-interacting protein KEULE, putative, expressed
2,5	4056	LOC_Os02g15880.2|11972.m33634|protein expressed protein
5,5	19461	LOC_Os02g13960.1|11972.m06642|protein U-box domain containing protein, expressed
8,9	10064	LOC_Os02g11740.1|11972.m06423|protein Plastidic ATP/ADP-transporter, putative, expressed
3,6	11368	LOC_Os02g11640.1|11972.m06413|protein Flavonol-3-O-glycoside-7-O-glucosyltransferase 1, putative, expressed
2,6	13740	LOC_Os02g11050.1|11972.m06403|protein 26S protease regulatory subunit 8, putative, expressed
10,2	26395	LOC_Os02g10880.1|11972.m06386|protein Indole-3-acetate beta-glucosyltransferase, putative, expressed
3,6	6207	LOC_Os02g10290.1|11972.m06327|protein Copper-transporting ATPase 3, putative, expressed
12,1	28943	LOC_Os02g09490.1|11972.m06246|protein Cinnamyl-alcohol dehydrogenase, putative, expressed
4,5	7756	LOC_Os02g09130.1|11972.m06210|protein ankryin repeat S-palmitoyl transferase, putative, expressed
9,4	12809	LOC_Os02g08100.1|11972.m06156|protein 4-coumarate-CoA ligase 1, putative, expressed
4,0	27594	LOC_Os02g07760.1|11972.m06122|protein Succinate semialdehyde dehydrogenase, mitochondrial precursor
3,0	35751	LOC_Os02g07720.1|11972.m06118|protein Choline-phosphate cytidylyltransferase B, putative, expressed
3,1	8906	LOC_Os02g07630.1|11972.m06110|protein Copper-transporting ATPase RAN1, putative, expressed
11,0	28798	LOC_Os02g07040.1|11972.m06051|protein Rhodanese-like domain containing protein, expressed
2,2	34963	LOC_Os02g05870.2|11972.m33560|protein expressed protein
2,4	30338	LOC_Os02g05710.1|11972.m05919|protein expressed protein
5,4	1126	LOC_Os02g05330.1|11972.m05881|protein Eukaryotic initiation factor 4A, putative, expressed
2,1	19702	LOC_Os02g04850.1|11972.m05833|protein expressed protein
5,2	32114	LOC_Os02g04170.2|11972.m33538|protein L-aspartate oxidase family protein, expressed
2,4	10288	LOC_Os02g04170.2|11972.m33538|protein L-aspartate oxidase family protein, expressed
2,2	4772	LOC_Os02g04020.1|11972.m05751|protein phosphatidylinositol transfer-like protein III, putative, expressed
5,5	27355	LOC_Os02g03280.2|11972.m33327|protein Bax inhibitor-1, putative, expressed
2,7	27869	LOC_Os02g03280.2|11972.m33327|protein Bax inhibitor-1, putative, expressed
9,0	37431	LOC_Os02g03260.2|11972.m33530|protein homoaconitate hydratase family protein, expressed
4,1	34747	LOC_Os02g03260.2|11972.m33530|protein homoaconitate hydratase family protein, expressed
2,6	13380	LOC_Os02g02860.1|11972.m05636|protein Glutamyl-tRNA synthetase, putative, expressed

31,7	27228	LOC_Os02g01590.1|11972.m05509|protein Beta-fructofuranosidase 1 precursor, putative, expressed
3,4	17206	LOC_Os02g01590.1|11972.m05509|protein Beta-fructofuranosidase 1 precursor, putative, expressed
2,1	13694	LOC_Os02g01590.1|11972.m05509|protein Beta-fructofuranosidase 1 precursor, putative, expressed
2,2	6212	LOC_Os02g01240.1|11972.m05474|protein Ankyrin-1, putative, expressed
2,1	24406	LOC_Os01g73500.1|11971.m13378|protein expressed protein
4,4	15685	LOC_Os01g73120.1|11971.m13341|protein membrane protein, putative, expressed
2,3	7065	LOC_Os01g72240.1|11971.m13259|protein Uridine 5'-monophosphate synthase, putative, expressed
3,5	1624	LOC_Os01g71760.1|11971.m13215|protein amino acid permease family protein, putative
5,8	8037	LOC_Os01g71270.1|11971.m13170|protein Eukaryotic peptide chain release factor subunit 1-2, putative, expressed
2,1	34119	LOC_Os01g70780.1|11971.m13123|protein SVP1-like protein 2, putative, expressed
2,2	8417	LOC_Os01g70170.1|11971.m13065|protein Transaldolase 2, putative, expressed
4,9	31648	LOC_Os01g68770.1|11971.m12979|protein Selenium-binding protein, putative, expressed
2,2	1638	LOC_Os01g68770.1|11971.m12979|protein Selenium-binding protein, putative, expressed
3,6	34812	LOC_Os01g68320.5|11971.m43342|protein ATP-dependent RNA helicase DBP2, putative, expressed
2,1	16397	LOC_Os01g67290.1|11971.m12832|protein cyclin, putative, expressed
8,4	114	LOC_Os01g67054.2|11971.m43324|protein Calreticulin precursor, putative, expressed
2,9	21630	LOC_Os01g67054.1|11971.m12809|protein Calreticulin precursor, putative, expressed
3,6	21054	LOC_Os01g66940.1|11971.m12799|protein Fructokinase, putative, expressed
2,6	22317	LOC_Os01g66690.1|11971.m12776|protein tetratricopeptide repeat, putative, expressed
2,1	21989	LOC_Os01g66350.1|11971.m12743|protein expressed protein
2,7	15423	LOC_Os01g65310.1|11971.m12644|protein Non-imprinted in Prader-Willi/Angelman syndrome region protein 2homolog
3,7	27640	LOC_Os01g63854.1|11971.m12500|protein expressed protein
2,8	14686	LOC_Os01g63854.1|11971.m12500|protein expressed protein
2,2	34641	LOC_Os01g63820.1|11971.m12497|protein Double-stranded RNA binding motif family protein, expressed
7,9	31489	LOC_Os01g63210.1|11971.m12438|protein SOUL heme-binding family protein, putative, expressed
5,0	30196	LOC_Os01g63210.1|11971.m12438|protein SOUL heme-binding family protein, putative, expressed
2,4	25302	LOC_Os01g62900.1|11971.m12410|protein Delta 1-pyrroline-5-carboxylate synthetase, putative, expressed
2,1	27522	LOC_Os01g62850.1|11971.m12405|protein integral membrane protein, putative, expressed
3,2	28714	LOC_Os01g61970.1|11971.m12320|protein Lung seven transmembrane receptor family protein, expressed
2,3	24262	LOC_Os01g61500.1|11971.m12276|protein BCL-2 binding anthanogene-1, putative, expressed
6,8	26492	LOC_Os01g61460.1|11971.m12272|protein expressed protein
2,0	35629	LOC_Os01g61410.1|11971.m43249|protein Rotenone-insensitive NADH-ubiquinone oxidoreductase, mitochondrial precursor
4,9	12116	LOC_Os01g61044.2|11971.m12231|protein Transmembrane amino acid transporter protein, expressed
4,5	2723	LOC_Os01g59660.2|11971.m42739|protein Transcription factor GAMYB, putative, expressed
3,3	35499	LOC_Os01g58790.1|11971.m12021|protein 4-diphosphocytidyl-2-C-methyl-D-erythritol kinase, chloroplast precursor
3,7	13967	LOC_Os01g58420.1|11971.m11985|protein AP2 domain containing protein, expressed
2,2	25044	LOC_Os01g58380.1|11971.m11981|protein 3-hydroxybutyryl-CoA dehydrogenase, putative, expressed
3,8	8313	LOC_Os01g58114.2|11971.m11956|protein expressed protein
3,3	28641	LOC_Os01g57110.2|11971.m42732|protein SNF2 domain-containing protein, putative, expressed
2,8	29493	LOC_Os01g56560.1|11971.m11813|protein aromatic-rich family protein, putative, expressed
2,6	6955	LOC_Os01g56470.1|11971.m11803|protein Mal d 1-associated protein, putative, expressed
6,8	13155	LOC_Os01g56450.1|11971.m11801|protein expressed protein
3,8	21551	LOC_Os01g55940.1|11971.m11750|protein indole-3-acetic acid-amido synthetase GH3.1, putative, expressed
2,9	37203	LOC_Os01g55350.1|11971.m11694|protein Phosphoenolpyruvate carboxylase, housekeeping isozyme, putative, expressed
2,6	8909	LOC_Os01g54870.1|11971.m11648|protein 60S ribosomal protein L18a, putative, expressed
2,9	36783	LOC_Os01g54860.1|11971.m11647|protein 3-hydroxyisobutyryl-coenzyme A hydrolase, putative, expressed
2,6	36398	LOC_Os01g54860.1|11971.m11647|protein 3-hydroxyisobutyryl-coenzyme A hydrolase, putative, expressed
4,1	11176	LOC_Os01g53880.5|11971.m43183|protein AUX/IAA family protein, expressed
3,1	33890	LOC_Os01g53810.1|11971.m11545|protein Transferrin receptor-like dimerisation domain containing protein, expressed

4,2	31182	LOC_Os01g53730.1|11971.m11539|protein expressed protein
3,6	12275	LOC_Os01g53730.1|11971.m11539|protein expressed protein
10,2	1596	LOC_Os01g51250.1|11971.m11306|protein Mitochondrial carrier protein, expressed
6,0	1486	LOC_Os01g49830.1|11971.m11168|protein DNA-binding protein RAV1, putative, expressed
5,1	4451	LOC_Os01g48190.3|11971.m43136|protein Drought induced 19 protein containing protein, expressed
7,4	86	LOC_Os01g48190.2|11971.m97533|protein Drought induced 19 protein containing protein, expressed
3,7	9477	LOC_Os01g48190.2|11971.m97533|protein Drought induced 19 protein containing protein, expressed
5,4	10545	LOC_Os01g48130.1|11971.m11005|protein no apical meristem, putative, expressed
2,6	2458	LOC_Os01g48130.1|11971.m11005|protein no apical meristem, putative, expressed
5,2	15704	LOC_Os01g48000.1|11971.m10992|protein D-mannose binding lectin family protein, expressed
2,1	33205	LOC_Os01g46710.1|11971.m10868|protein transposon protein, putative, CACTA, En/Spm sub-class, expressed
10,5	33610	LOC_Os01g45910.1|11971.m10791|protein Plastidic ATP/ADP-transporter, putative, expressed
2,2	27713	LOC_Os01g45660.1|11971.m10767|protein hypothetical protein
5,2	35810	LOC_Os01g42870.1|11971.m10551|protein Transferase family protein, expressed
3,7	6190	LOC_Os01g40420.1|11971.m97524|protein CBS domain containing protein, expressed
2,6	18358	LOC_Os01g40420.1|11971.m97524|protein CBS domain containing protein, expressed
4,1	4235	LOC_Os01g38970.1|11971.m10177|protein Carbamoyl-phosphate synthase large chain, putative, expressed
8,2	33062	LOC_Os01g36950.3|11971.m43051|protein expressed protein
3,3	9498	LOC_Os01g36950.2|11971.m09991|protein expressed protein
3,2	933	LOC_Os01g36950.2|11971.m09991|protein expressed protein
2,3	8822	LOC_Os01g36950.2|11971.m09991|protein expressed protein
6,4	7358	LOC_Os01g35040.1|11971.m09806|protein PRLI-interacting factor K, putative, expressed
5,8	18796	LOC_Os01g35040.1|11971.m09806|protein PRLI-interacting factor K, putative, expressed
4,6	2950	LOC_Os01g34480.1|11971.m09753|protein NAD dependent epimerase/dehydratase family protein, expressed
5,0	26144	LOC_Os01g33000.1|11971.m09617|protein expressed protein
4,9	3022	LOC_Os01g33000.1|11971.m09617|protein expressed protein
2,8	34623	LOC_Os01g32770.1|11971.m09596|protein expressed protein
2,8	588	LOC_Os01g32130.1|11971.m09539|protein expressed protein
3,5	30159	LOC_Os01g27730.1|11971.m09221|protein expressed protein
2,4	13017	LOC_Os01g23640.2|11971.m42998|protein Mov34/MPN/PAD-1 family protein, expressed
4,7	27752	LOC_Os01g22900.2|11971.m42995|protein beta-fructofuranosidase, putative, expressed
2,3	21809	LOC_Os01g22010.3|11971.m42990|protein S-adenosylmethionine synthetase 2, putative, expressed
2,7	34380	LOC_Os01g18850.3|11971.m42976|protein SBP domain containing protein, expressed
2,5	19120	LOC_Os01g18100.1|11971.m08352|protein rhomboid family protein, putative, expressed
3,7	16198	LOC_Os01g16750.1|11971.m08271|protein Flavin-binding monooxygenase-like family protein
18,8	8244	LOC_Os01g15640.1|11971.m08162|protein No apical meristem protein, expressed
2,3	19998	LOC_Os01g14860.4|11971.m43459|protein Glycogen synthase kinase-3 homolog MsK-3, putative, expressed
2,1	4905	LOC_Os01g14860.4|11971.m43459|protein Glycogen synthase kinase-3 homolog MsK-3, putative, expressed
2,5	21929	LOC_Os01g13210.2|11971.m42933|protein DREPP plasma membrane polypeptide family protein, expressed
2,9	11306	LOC_Os01g12890.1|11971.m07902|protein expressed protein
2,7	105	LOC_Os01g11920.1|11971.m07808|protein retrotransposon protein, putative, unclassified
4,6	13430	LOC_Os01g10680.2|11971.m42619|protein expressed protein
3,2	24467	LOC_Os01g10680.2|11971.m42619|protein expressed protein
4,0	11051	LOC_Os01g10610.1|11971.m07682|protein BES1/BZR1 homolog protein 4, putative, expressed
2,4	30855	LOC_Os01g10250.2|11971.m42618|protein hydrolase, alpha/beta fold family protein, expressed
3,9	12454	LOC_Os01g09670.1|11971.m07591|protein Pollen-specific protein SF21, putative, expressed
2,6	6355	LOC_Os01g09420.1|11971.m07567|protein expressed protein
2,4	13665	LOC_Os01g09320.1|11971.m07557|protein NADP-dependent malic enzyme, chloroplast precursor, putative, expressed
3,9	19327	LOC_Os01g09300.3|11971.m42896|protein oxidoreductase, 2OG-Fe oxygenase family protein, putative, expressed

	4,9	7172	LOC_Os01g09300.2|11971.m07555|protein oxidoreductase, 2OG-Fe oxygenase family protein, putative, expressed
	4,5	33662	LOC_Os01g09010.1|11971.m07526|protein Transferase family protein, expressed
	3,0	3983	LOC_Os01g09010.1|11971.m07526|protein Transferase family protein, expressed
	2,4	19070	LOC_Os01g09000.1|11971.m07525|protein Glutaminyl-tRNA synthetase, putative, expressed
	2,2	18727	LOC_Os01g09000.1|11971.m07525|protein Glutaminyl-tRNA synthetase, putative, expressed
	2,8	15706	LOC_Os01g08270.3|11971.m97484|protein aminotransferase class I and II family protein, putative, expressed
	6,8	8261	LOC_Os01g08270.2|11971.m42886|protein aminotransferase class I and II family protein, putative, expressed
	3,7	25891	LOC_Os01g07530.1|11971.m07379|protein Raffinose synthase or seed imbibition protein Sip1 containing protein, expressed
	2,8	33753	LOC_Os01g07360.1|11971.m07362|protein CENP-E like kinetochore protein, putative, expressed
	4,0	19714	LOC_Os01g06660.1|11971.m07296|protein Pyruvate decarboxylase isozyme 1, putative, expressed
	4,8	19078	LOC_Os01g06460.1|11971.m07276|protein SAT5, putative, expressed
	2,0	34166	LOC_Os01g05540.1|11971.m07185|protein expressed protein
	3,3	9209	LOC_Os01g04350.1|11971.m07070|protein Hsp20/alpha crystallin family protein, expressed
	3,7	20566	LOC_Os01g04010.1|11971.m07037|protein expressed protein
	2,1	1139	LOC_Os01g03050.1|11971.m06944|protein Oxidoreductase NAD-binding domain containing protein, expressed
	2,2	23232	LOC_Os01g02200.1|11971.m06862|protein armadillo/beta-catenin repeat family protein, putative, expressed
**Unknown**	1766,5	23060	
	23,3	12522	
	16,9	14234	
	15,5	9372	
	13,8	5303	
	13,1	33267	
	12,0	8069	
	11,9	6873	
	11,4	25252	
	11,3	24888	
	10,3	23050	
	9,9	2126	
	9,4	22272	
	8,9	23174	
	8,9	37766	
	8,2	8740	
	8,2	2963	
	8,1	28047	
	7,7	1866	
	7,6	27791	
	7,5	21310	
	7,3	21875	
	7,0	31311	
	6,9	4172	
	6,9	37243	
	6,9	17923	
	6,5	28579	
	6,4	11527	
	6,3	24320	
	6,2	25722	
	5,6	27042	
	5,4	7319	
	5,4	19737	

5,2	14135
5,1	10453
5,0	25685
4,9	23185
4,8	13943
4,7	11462
4,7	34505
4,6	7790
4,6	36070
4,4	35656
4,3	18715
4,3	19263
4,3	2801
4,3	12978
4,3	5988
4,2	19339
4,2	23365
4,1	651
4,0	18247
4,0	6900
3,9	16185
3,9	7385
3,9	7490
3,9	34246
3,9	26412
3,8	18307
3,8	23710
3,8	22335
3,8	31885
3,8	21284
3,7	15973
3,7	13577
3,7	15146
3,6	33953
3,6	2706
3,5	22521
3,5	23950
3,5	14598
3,5	33944
3,5	1566
3,4	2606
3,4	2704
3,4	23584
3,3	29041
3,2	11265
3,2	14068
3,2	5901
3,1	1314
3,1	17343

3,0	17559
3,0	7876
3,0	17423
3,0	34811
2,9	6377
2,9	14782
2,9	8848
2,9	9966
2,9	8855
2,9	20775
2,8	16515
2,8	5916
2,8	17672
2,7	25139
2,7	19798
2,7	31651
2,7	16262
2,7	7807
2,7	21014
2,7	26186
2,7	28983
2,6	7346
2,6	34891
2,6	7842
2,6	11315
2,6	1806
2,6	37325
2,6	14928
2,6	1901
2,6	6645
2,5	34171
2,5	2447
2,5	36796
2,5	25482
2,5	25151
2,5	35154
2,5	14478
2,5	10949
2,5	11202
2,5	34804
2,4	17870
2,4	30112
2,4	32854
2,4	8459
2,4	30750
2,4	29612
2,4	30057
2,4	32954
2,4	15569

Gene classes	Fold Change	Probe ID	Annotation
	2,4	24093	
	2,4	36437	
	2,3	19289	
	2,3	32151	
	2,3	28780	
	2,3	23236	
	2,3	34599	
	2,3	16475	
	2,3	35032	
	2,3	21079	
	2,3	1200	
	2,3	2490	
	2,3	4281	
	2,2	32084	
	2,2	35638	
	2,2	26530	
	2,2	8654	
	2,2	35883	
	2,2	24808	
	2,2	15338	
	2,2	14318	
	2,2	27111	
	2,2	33858	
	2,2	6812	
	2,2	28698	
	2,1	27307	
	2,1	16711	
	2,1	14754	
	2,1	25073	
	2,1	23803	
	2,1	7013	
	2,1	19214	
	2,1	12135	
	2,1	27534	
	2,0	21790	
	2,0	17947	
	2,0	13954	
	2,0	1664	
	2,0	31614	
	2,0	2286	
	2,0	21962	
	2,0	37641	
	2,0	2133	
	2,0	19311	

**Nobeokabouzu 7 dai specific up-regulated genes**			
**Gene classes**	**Fold Change**	**Probe ID**	**Annotation**
**JA and ET related genes**			
Lipoxygenase	2,1	1076	LOC_Os03g49380.3|11973.m35371|protein Lipoxygenase 3, putative, expressed
Lipoxygenase	2,1	27579	LOC_Os03g49380.3|11973.m35371|protein Lipoxygenase 3, putative, expressed

Lipoxygenase	2,0	8762	LOC_Os03g49380.3|11973.m35371|protein Lipoxygenase 3, putative, expressed
Lipid metabolism	2,2	33349	LOC_Os02g17390.1|11972.m06982|protein Peroxisomal fatty acid beta-oxidation multifunctional protein, putative, expressed
Lipid metabolism	14,2	7118	LOC_Os01g62010.1|11971.m12324|protein Monoglyceride lipase, putative, expressed
**GDSL-lipase**			
	2,6	1380	LOC_Os06g06250.1|11976.m05351|protein GDSL-like Lipase/Acylhydrolase family protein, expressed
	2,4	30626	LOC_Os05g44200.1|11975.m08546|protein GDSL-like Lipase/Acylhydrolase family protein, expressed
**Proteolysis**			
	2,3	4740	LOC_Os03g27590.1|11973.m08086|protein Serine carboxypeptidase family protein, expressed
**Secondary metabolism & detoxification**			
Cytochrome P450s	2,4	20587	LOC_Os09g38620.1|11979.m06803|protein NADPH-cytochrome P450 reductase, putative, expressed
Cytochrome P450s	2,5	988	LOC_Os03g55800.1|11973.m10491|protein Cytochrome P450 74A1, chloroplast precursor, putative, expressed
	2,8	8322	LOC_Os03g37490.1|11973.m08858|protein MATE efflux family protein, expressed
ABC transporter	2,5	32845	LOC_Os04g52900.1|11974.m10183|protein ABC transporter, putative, expressed
Glutathione S-transferase			
	2,7	17633	LOC_Os12g34380.1|11982.m07232|protein Glutathione synthetase, chloroplast precursor, putative, expressed
	2,3	28817	LOC_Os07g44290.1|11977.m08650|protein CBL-interacting serine/threonine-protein kinase 1, putative, expressed
	5,2	33465	LOC_Os07g17010.1|11977.m06142|protein Chalcone and stilbene synthases, N-terminal domain containing protein, expressed
	2,4	35594	LOC_Os05g05620.1|11975.m05089|protein glutathione S-transferase GSTF1, putative, expressed
	2,1	13158	LOC_Os03g43440.1|11973.m09390|protein CBL-interacting serine/threonine-protein kinase 11, putative, expressed
**Transcription & signallig**			
	3,9	32104	LOC_Os08g04630.1|11978.m04606|protein EF hand family protein, expressed
	2,6	9402	LOC_Os02g08500.1|11972.m06196|protein myb-like DNA-binding domain, SHAQKYF class family protein, expressed
	2,1	22002	LOC_Os11g17380.1|11981.m05821|protein Protein kinase domain containing protein
	2,5	3489	LOC_Os07g35690.2|11977.m07825|protein Protein kinase domain containing protein, expressed
	2,6	12554	LOC_Os07g35260.1|11977.m07782|protein Protein kinase domain containing protein, expressed
	2,1	24026	LOC_Os04g44910.1|11974.m35044|protein lectin protein kinase, putative, expressed
	2,4	26265	LOC_Os02g12670.1|11972.m06514|protein protein kinase family protein, putative, expressed
	2,8	17779	LOC_Os05g03760.1|11975.m04910|protein Zinc finger C-x8-C-x5-C-x3-H type family protein, expressed
	2,5	11870	LOC_Os03g08920.1|11973.m06398|protein Zinc finger, C3HC4 type family protein, expressed
**Un categorized**			
	2,8	30524	LOC_Os12g24540.1|11982.m06285|protein PHD-finger family protein, expressed
	2,3	32380	LOC_Os12g04440.2|11982.m26829|protein 2-isopropylmalate synthase B, putative, expressed
	2,1	4588	LOC_Os11g25060.1|11981.m06430|protein oxidoreductase, 2OG-Fe oxygenase family protein
	2,1	26776	LOC_Os11g07440.1|11981.m04940|protein Neutral/alkaline invertase, putative, expressed
	3,1	26266	LOC_Os10g43060.1|11980.m22001|protein expressed protein
	2,7	20841	LOC_Os10g42620.1|11980.m07015|protein NAD dependent epimerase/dehydratase family protein, expressed
	2,0	22646	LOC_Os10g35150.1|11980.m06338|protein expressed protein
	2,4	36612	LOC_Os10g32820.1|11980.m06118|protein 60S ribosomal protein L21, putative, expressed
	2,3	36435	LOC_Os10g32680.1|11980.m06104|protein Uncharacterized conserved protein, putative, expressed
	2,2	7080	LOC_Os10g32080.1|11980.m06056|protein Exostosin family protein
	2,0	20368	LOC_Os09g39560.1|11979.m06894|protein Genetic modifier, putative, expressed
	3,0	26486	LOC_Os09g37800.1|11979.m06727|protein D-mannose binding lectin family protein
	2,2	11290	LOC_Os07g44960.3|11977.m29402|protein nodulation protein, putative, expressed
	2,4	26272	LOC_Os07g35050.1|11977.m07761|protein F-box domain containing protein, expressed
	2,1	1801	LOC_Os07g34589.6|11977.m29388|protein Protein translation factor SUI1 homolog, putative, expressed
	4,1	27532	LOC_Os07g08430.1|11977.m05299|protein Indole-3-glycerol phosphate lyase, chloroplast precursor, putative, expressed
	2,3	4011	LOC_Os07g01020.1|11977.m04583|protein pyridoxin biosynthesis protein ER1, putative, expressed
	2,1	25489	LOC_Os07g01020.1|11977.m04583|protein pyridoxin biosynthesis protein ER1, putative, expressed
	2,5	15864	LOC_Os06g46440.1|11976.m09170|protein expressed protein

	2,5	844	LOC_Os06g08032.1|11976.m05526|protein oxidoreductase, 2OG-Fe oxygenase family protein
	2,7	21022	LOC_Os06g05120.1|11976.m05241|protein hypothetical protein
	2,7	11188	LOC_Os06g05120.1|11976.m05241|protein hypothetical protein
	2,6	27560	LOC_Os05g49770.1|11975.m09047|protein CTP synthase, putative, expressed
	3,3	3012	LOC_Os05g46460.5|11975.m27943|protein hydrolase, alpha/beta fold family protein, putative, expressed
	2,1	23099	LOC_Os05g38150.2|11975.m27606|protein Delta 1-pyrroline-5-carboxylate synthetase, putative, expressed
	2,2	5564	LOC_Os05g25640.1|11975.m06857|protein Trans-cinnamate 4-monooxygenase, putative, expressed
	2,8	24651	LOC_Os05g05840.1|11975.m05111|protein Histidyl-tRNA synthetase, putative, expressed
	2,2	31031	LOC_Os05g03780.1|11975.m04912|protein cation diffusion facilitator family transporter containing protein, expressed
	2,1	28199	LOC_Os05g01030.1|11975.m04643|protein Phospholipid-transporting ATPase 10, putative, expressed
	5,7	11522	LOC_Os04g57200.1|11974.m35503|protein heavy metal-associated domain containing protein, expressed
	2,0	34566	LOC_Os04g55650.2|11974.m78923|protein Low-temperature-induced cysteine proteinase precursor, putative, expressed
	3,3	8549	LOC_Os04g48390.1|11974.m09775|protein SPX domain-containing protein, putative, expressed
	2,5	37508	LOC_Os03g62388.1|11973.m11115|protein hypothetical protein
	4,6	29797	LOC_Os03g50870.1|11973.m10072|protein expressed protein
	2,7	3338	LOC_Os03g43720.4|11973.m35326|protein major facilitator superfamily protein, expressed
	2,0	14362	LOC_Os03g41419.1|11973.m09208|protein Serpin family protein, expressed
	2,4	28622	LOC_Os03g25750.1|11973.m07922|protein expressed protein
	2,4	9242	LOC_Os03g20310.4|11973.m35194|protein Transcription factor HBP-1b, putative, expressed
	2,1	26324	LOC_Os03g20020.1|11973.m07415|protein beta-fructofuranosidase, putative, expressed
	3,2	27373	LOC_Os03g13840.3|11973.m34814|protein Senescence-associated protein, expressed
	2,3	36385	LOC_Os03g06200.1|11973.m06140|protein Phosphoserine aminotransferase, chloroplast precursor, putative, expressed
	2,1	8685	LOC_Os03g04000.1|11973.m05932|protein AMP-binding enzyme family protein, expressed
	2,3	22642	LOC_Os02g58150.2|11972.m77864|protein expressed protein
	2,1	34115	LOC_Os02g55970.1|11972.m10621|protein Clathrin assembly protein, putative, expressed
	2,1	1312	LOC_Os02g55030.1|11972.m10530|protein hydrolase, NUDIX family protein, expressed
	2,2	297	LOC_Os02g48790.3|11972.m33446|protein AML1, putative, expressed
	2,1	6959	LOC_Os02g31030.1|11972.m08246|protein glycerophosphoryl diester phosphodiesterase family protein, putative, expressed
	2,9	1287	LOC_Os02g08540.1|11972.m06200|protein expressed protein
	2,2	33604	LOC_Os02g07880.1|11972.m06134|protein expressed protein
	2,4	14850	LOC_Os02g02120.1|11972.m05562|protein wall-associated kinase 3, putative, expressed
	2,2	8379	LOC_Os01g72690.2|11971.m42804|protein ATP-NAD kinase family protein, putative, expressed
	2,5	568	LOC_Os01g70380.1|11971.m13086|protein Serine palmitoyltransferase 2, putative, expressed
	2,1	3615	LOC_Os01g65410.1|11971.m12653|protein Serine hydroxymethyltransferase, mitochondrial precursor, putative, expressed
	4,1	34628	LOC_Os01g62900.1|11971.m12410|protein Delta 1-pyrroline-5-carboxylate synthetase, putative, expressed
	2,5	21587	LOC_Os01g61990.1|11971.m12322|protein ankyrin repeat family protein, putative, expressed
	2,3	14398	LOC_Os01g61460.1|11971.m12272|protein expressed protein
	2,4	21635	LOC_Os01g57720.1|11971.m11921|protein expressed protein
	3,5	7509	LOC_Os01g48960.1|11971.m11085|protein Glutamate synthase, chloroplast precursor, putative, expressed
	2,5	32432	LOC_Os01g48960.1|11971.m11085|protein Glutamate synthase, chloroplast precursor, putative, expressed
	2,2	8879	LOC_Os01g40630.1|11971.m10334|protein Lysine Decarboxylase, putative, expressed
	2,3	15213	LOC_Os01g36070.1|11971.m09904|protein MtN3/saliva family protein, expressed
	2,1	30613	LOC_Os01g15640.1|11971.m08162|protein No apical meristem protein, expressed
	2,0	28789	LOC_Os01g11240.1|11971.m07743|protein expressed protein
	2,7	15948	LOC_Os01g09570.1|11971.m07582|protein Phosphofructokinase family protein, expressed
**Unknown**	90,0	1615	
	17,0	15503	
	4,5	13498	
	3,7	23498	

3,6	4368
3,2	9366
2,9	24076
2,7	22370
2,5	30798
2,3	2617
2,2	21055
2,1	34618
2,1	14823
2,1	12314
2,1	2918
2,0	1896
2,0	27805
